# Synthesis and Biological Evaluation of Novel Cinnamic Acid-Based Antimicrobials

**DOI:** 10.3390/ph15020228

**Published:** 2022-02-15

**Authors:** Marina Mingoia, Carmela Conte, Annalisa Di Rienzo, Marilisa Pia Dimmito, Lorella Marinucci, Gloria Magi, Hasan Turkez, Maria Concetta Cufaro, Piero Del Boccio, Antonio Di Stefano, Ivana Cacciatore

**Affiliations:** 1 Department of Biomedical Sciences and Public Health, Medical School, Polytechnic University of Marche, 60121 Ancona, Italy; m.mingoia@univpm.it (M.M.); g.magi@univpm.it (G.M.); 2 Department of Pharmaceutical Sciences, University of Perugia, Via Fabretti, 48, 06123 Perugia, Italy; carmela.conte@unipg.it; 3 Department of Pharmacy, University “G. d’Annunzio” of Chieti-Pescara, Via dei Vestini 31, 66100 Chieti Scalo, Italy; annalisa.dirienzo@unich.it (A.D.R.); marilisa.dimmito@unich.it (M.P.D.); maria.cufaro@unich.it (M.C.C.); piero.delboccio@unich.it (P.D.B.); antonio.distefano@unich.it (A.D.S.); 4 Department of Medicine and Surgery, University of Perugia, S. Andrea Delle Fratte, 06156 Perugia, Italy; lorella.marinucci@unipg.it; 5 Department of Medical Biology, Faculty of Medicine, Ataturk University, 25240 Erzurum, Turkey; hasanturkez@gmail.com; 6 Center for Advanced Studies and Technology (CAST), University “G. d’Annunzio” of Chieti-Pescara, Via dei Vestini 31, 66100 Chieti Scalo, Italy

**Keywords:** antimicrobial, carvacrol, cinnamic acid, skin infection, wound healing

## Abstract

The main antimicrobial resistance (AMR) nosocomial strains (ESKAPE pathogens such as *Enterococcus faecium*, *Staphylococcus aureus*, *Klebsiella pneumoniae*, *Acinetobacter baumannii*, *Pseudomonas aeruginosa*, and *Enterobacter* spp.) are the most widespread bacteria in cutaneous infections. In this work we report the synthesis, in silico skin permeability prediction, antimicrobial, antibiofilm, and wound healing properties of novel cinnamic acid-based antimicrobials (**DM1–11**) as novel antibacterial drugs for the treatment of ESKAPE-related skin infections. Antimicrobial and wound healing scratch assays were performed to evaluate the antibacterial properties of **DM1–11**. In silico skin permeability capabilities of **DM1–11** were evaluated using Swiss-ADME online database. Cytotoxicity assays were performed on keratinocytes and fibroblasts. **DM2**, bearing a catechol group on the aromatic ring of the cinnamic portion of the molecule, possesses a significant antibacterial activity against *S. aureus* (MIC range 16–64 mg/L) and contrasts the biofilm-mediated *S. epidermidis* infection at low concentrations. Wound healing assays showed that wound closure in 48 h was observed in **DM2**-treated keratinocytes with a better healing pattern at all the used concentrations (0.1, 1.0, and 10 µM). A potential good skin permeation for **DM2**, that could guarantee its effectiveness at the target site, was also observed. Cytotoxicity studies revealed that **DM2** may be a safe compound for topical use. Taking together all these data confirm that **DM2** could represent a safe wound-healing topical agent for the treatment of skin wound infections caused by two of main Gram-positive bacteria belonging to ESKAPE microorganisms.

## 1. Introduction

Antimicrobial resistance (AMR) is one of the most important medical challenges that scientists must deal with. As reported by the World Health Organization (WHO), antibiotics are becoming progressively ineffective as drug resistance extends worldwide leading to infections that are difficult or impossible to manage [1]. Several reasons are responsible for AMR such as an inappropriate use of antibiotics, microbial behavior, gene transfer, or the addition of antibiotics to agricultural feed [2]. Bacteria develop several mechanisms of AMR as drug inactivation through production of enzymes and/or biofilms, limitation of drug uptake, mutation /alteration of the drug target, and use of efflux pump [3]. Consequently, some infections that are difficult to control lead to disease spreading with increased probability of fatality and enhance economic and social costs. 

Bacteria responsible for AMR have been defined by the Infectious Disease Society of America and classed as ESKAPE pathogens: *Enterococcus faecium*, *Staphylococcus aureus*, *Klebsiella pneumoniae*, *Acinetobacter baumannii*, *Pseudomonas aeruginosa*, and *Enterobacter* spp. [4]. ESKAPE pathogens that belong to both Gram-positive and -negative bacteria are mainly responsible for nosocomial infections such as central line-associated bloodstream infections, catheter-associated urinary tract infections, surgical wound infections, and ventilator-associated pneumonia [5]. About 15% of all hospitalized patients suffer from these infections, as reported by WHO [6]. Moreover, ESKAPE pathogens have also been found in cutaneous lesions rendering the chronic wounds difficult to manage [7]. The production of virulence factors by ESKAPE microorganisms generates a slowdown of the wound healing process in both skin acute and chronic wounds. *E. faecium* has been considered to be mainly responsible for skin and soft tissue infections (SSTIs), as reported by Weintrob et al. [8]; *S. aureus* has been implicated in the progression of diabetic ulcers to osteomyelitis [9]. However, *Enterobacter* spp. are also involved in SSTIs [10]. In addition, *K. pneumoniae* has been one of the four main microorganisms isolated from contaminated wounds in hospitalized burn patients similar to *A. baumannii* [11,12]. *P. aeruginosa* causes local and systemic infections especially in immunosuppressed patients; notably it is involved in pulmonary complications in cystic fibrosis-affected patients [13].

To date, the treatment of ESKAPE-related skin infections is an important challenge since these microorganisms, through the acquisition of resistance and virulence determinants, are very difficult to eradicate. Promising trends in combating AMR utilize traditional and natural antimicrobials, plant and microbial derivatives, and/or nanomaterials [14,15,16]. Small molecules such as natural cinnamic acid, its derivatives, or hybrid molecules exhibit antibacterial, antifungal, and anti-inflammatory properties [17,18]. Cinnamic acid can exist as both *cis*- and *trans*-forms in nature, even though the *trans*-form is predominant because it is more stable than the *cis*-form. It is one of such privileged multitarget structures endowed with low toxicity. Its antimicrobial activity is weak against both Gram-positive and -negative bacteria showing MIC values higher than 5 mM except for *Mycobacterium tuberculosis* (MIC = 250–675 µM) [19]. However, as reported by the literature data, bacteria are more susceptible to synthetic cinnamic acids, esters, amides, while fungi are more sensitive to the action exerted by cinnamic aldehydes [20]. Moreover, several cinnamic acid derivatives, specifically those with the phenolic groups, have been classed as antioxidants with strong free radical scavenging properties, as part a study reported by Ferro et al. [21]. To date, there are not many reports about the use of cinnamic acids and/or its derivatives in skin infections. It has been described that the topical administration of cinnamaldehyde, at sub-inhibitory concentrations, significantly lowered the bacterial load in the wounds in *P. aeruginosa*-infected mice. The wound healing properties of cinnamaldehyde were related to its anti-inflammatory effect in reducing IL-17, vascular endothelial growth factor (VEGF), and nitric oxide in the wound beds.

**Figure 1:**
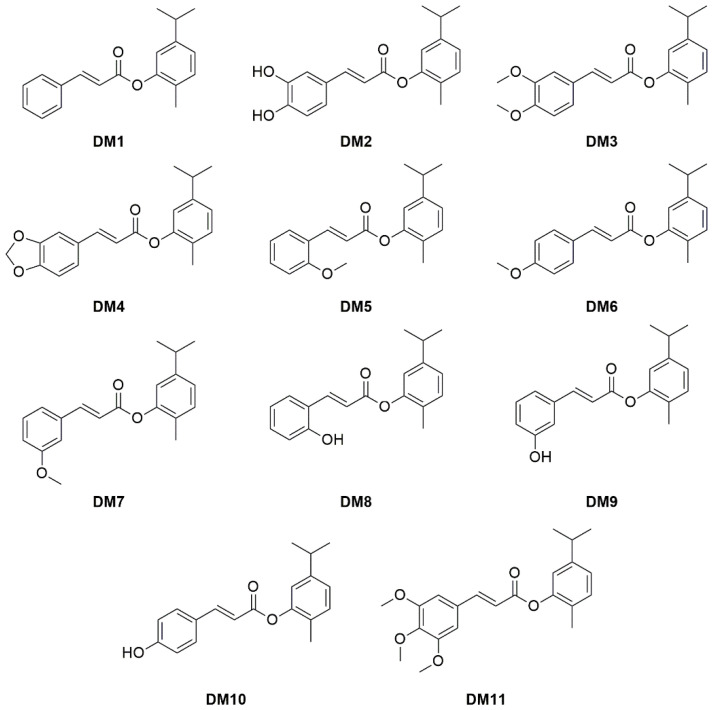
Chemical structures of cinnamic acid-based antimicrobials (**DM1–11**).

Considering the chemical structure of cinnamic acid, the presence of both benzene ring and a carboxylic group make the molecule a privileged scaffold to modify and get synthetic derivatives. Synthetic compounds combining two pharmaceutical entities in one molecule may be a successful strategy since these molecules could be more medically effective than their individual components for the treatment of complex skin infections. In this study, the conjugation of several hydroxy- and phenyl-substituted derivatives of *trans*-cinnamic acids with an antimicrobial pharmacophore like carvacrol (**DM1–11**) was investigated to select the best candidate to treat cutaneous infections caused by ESKAPE pathogens (Figure 1). Notably, carvacrol was chosen as the phytochemical component to conjugate to the series of cinnamic acids helpful by virtue of its wide natural antimicrobial, antioxidant, and wound healing properties [22]. It is a good antibacterial agent against both Gram-positive and -negative bacteria exerting its antimicrobial activity by disrupting bacterial membrane, bacterial lysis, and leading to leakage of intracellular contents.

The antimicrobial capabilities of the **DM1–11** compounds were evaluated against a wide panel of representative ESKPE pathogens, while antibiofilm activity was investigated against *S. aureus* ATCC 43300, *S. epidermidis* ATCC 35984, and *S. agalactiae* 343676, some of the most recurrent bacteria in wounds infections. The healing activity of **DM1–11** was assessed using an in vitro scratch wound assay on keratinocytes by determining the wound closure rate. As we conceived a potential topical administration of **DM1–11**, their cytotoxicity was also evaluated both on human fibroblasts and keratinocytes cell lines. 

## 2. Results

### 2.1. Chemistry

**Scheme 1:**
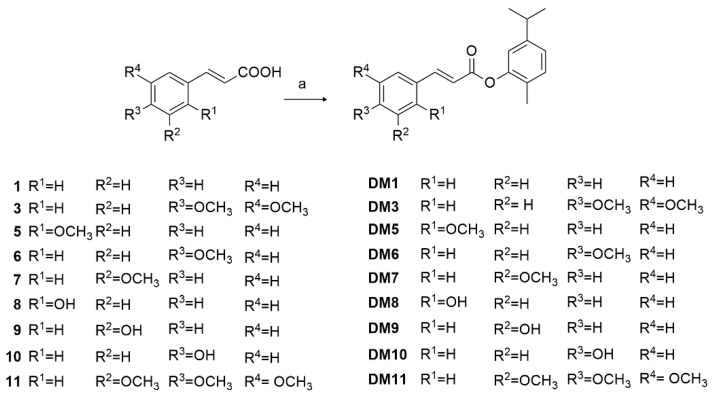
Reagents and conditions for **DM1**, **DM3–7**, and **DM11**: (**a**) Carvacrol, dry DCM, BOP, TEA at 0 °C, and thenat rt for 24 h. For **DM8–10**: (**a**) Carvacrol, dry DMF, EDC·HCl, and DMAP at 0 °C for 24 h.

**Scheme 2:**
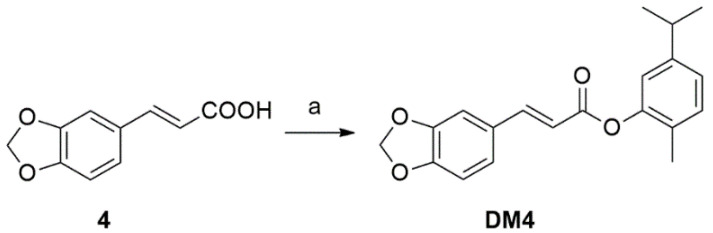
Reagents and conditions for **DM4**: (**a**) Carvacrol, dry DCM, BOP, TEA, at 0 °C, and then at rt for 24 h.

**Scheme 3:**
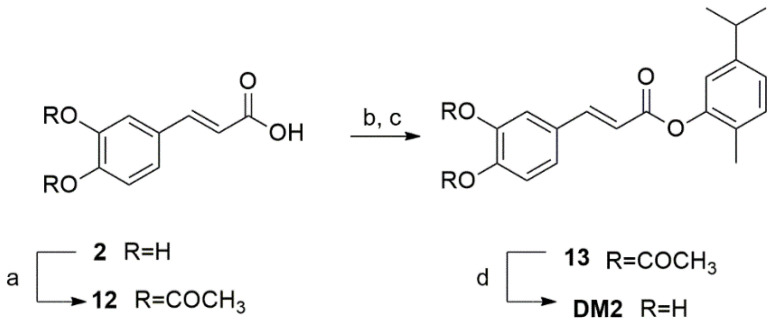
Reagents and conditions for **DM2**: (**a**) 1M NaOH, acetic anhydride at 0 °C for 30 min; (**b**) SOCl_2_, DMF at reflux for 4 h; (**c**) carvacrol, DCM, and pyridine at rt overnight; (**d**) guanidinium hydrochloride, TEA, DCM, 2 h, rt.

The synthetic routes of **DM1–11** (Figure 1) are illustrated in Scheme 1, Scheme 2 and Scheme 3. In short, compounds **DM1**, **DM3**, **DM5–7**, and **DM11** were obtained by esterification between the suitable *trans*-mono- or di-methoxy-phenyl-substituted derivative of cinnamic acids (**1**, **3**, **5–7**, or **11**), commercially available, in the presence of benzotriazol-1-yloxytris(dimethylamino)phosphonium hexafluorophosphate (BOP) and triethylamine (TEA) (Scheme 1). **DM4** was prepared following the same synthetic procedure above reported, but as the starting material *trans*-3,4-(methylendioxy)-cinnamic acid (**4**) was chosen for the reaction with carvacrol (Scheme 2). **DM8–10** were synthesized through conjugation among the appropriate *trans*-mono-hydroxy-phenyl-substituted derivative of cinnamic acids (**8–10**), commercially available, and carvacrol in the presence of 1-ethyl-3-(3-dimethylaminopropyl)carbodiimide hydrochloride (EDC·HCl), a water-soluble carbodiimide, and 4-dimethylaminopyridine (DMAP) (Scheme 1). 

Coupling reagents like carbodiimides, on the one hand, and phosphonium and aminium salts, on the other hand, can be useful to form an ester or amide linkage [23]. In our synthetic strategy, the use of carbodiimide-mediated coupling reagent (synthesis of **DM8–10**), such as EDC·HCl, required the addition of DMAP as base, because DMF, as the coupling medium, slowed down the preactivation process of the carboxylic acid residue of cinnamic acid derivatives (Scheme 1). In addition, benzotriazole-based chemistry was employed for the synthesis of *trans*-hydroxy-cinnamic antimicrobials **DM1**, **DM3–7**, and **DM11** to increase the yields as compared with carbodiimide-based reactions (from 20% to >50%).

A different synthetic strategy was selected for the preparation of **DM2** (Scheme 3). The presence of a catechol group on *trans*-3,4-dihydroxy-cinnamic acid (**2**) required a preliminary protection due to its reactivity and instability. Easy protection of catechol moiety of **2** was achieved using acetic anhydride in NaOH 1N at 0 °C for 30 min. As coupling reagents did not afford **13** in good yields (<30%), the activation of the carboxyl group of the *trans*-3,4-diacetyl-cinnamic derivative **2** with SOCl_2_ was necessary. Subsequent esterification of the *trans*-3,4-diacetyl-cynnamoil chloride with carvacrol in pyridine afforded the desired precursor **13** in good yield (79%). Deacetylation of the precursor **13**, performed using guanidium hydrochloride in TEA, afforded **DM2** (yield 59%). The structures of all synthetized compounds were confirmed by NMR techniques (NMR spectra are reported in the Supporting Information). The ^1^H-NMR spectra showed two proton signals in 6.2–6.6 ppm (1H, d) and 7.4–7.8 ppm (1H, d), confirming a trans-alkene group with coupling constant of 16 Hz. Two signals at 118–120 and 148–150 ppm showed sp^2^ carbon signals, with different chemical environments in the ^13^C-NMR spectra.

### 2.2. In Silico Skin Permeability Prediction Analysis

Topical antimicrobials offer an alternative in the therapy of moderate cutaneous infections. The limited incidence of toxicity reduced adverse effects, and resistance as compared with systemic antibiotics, make them valuable candidates in the wound healing process, treatment of localized acute and chronic infections, and in burns [24]. 

Estimations of **DM1–11** skin permeation were performed by using the SwissADME program. The skin permeation coefficient (*Kp*) of drugs in the stratum corneum is often used for these in silico assessment. Quantitatively, the Kp describes the rate of chemical permeation through the outermost layer of the epidermal skin. In silico parameters report that when Log *Kp* values are within the range from −8.0 to −1.0, the compounds showed good in silico physicochemical properties for skin permeability [25]. Prediction of skin permeation (Log *Kp*) indicated that **DM1–11**, displaying estimated Log *Kp* values between −4.2 and −5.01, could be easily absorbable by skin layers (Table 1). Considering their potential skin permeation and as many ESKAPE pathogens are common antibiotic-resistant bacteria colonizing skin wounds, the **DM1–11** compounds were tested as antimicrobials.

### 2.3. Antimicrobial and Antibiofilm Properties of ***DM1–11***

The antimicrobial activity of **DM1–11** (expressed as minimum inhibitory concentration (MIC)) was assayed against a wide panel of ESKAPE pathogens (Table 2). **DM1**, **DM3–7**, and **DM9–11** showed MIC values higher than 256 mg/L. **DM2** and **DM8** showed the best activity as compared with carvacrol, towards all Gram-positive species except for *S. agalactiae*. Nevertheless, **DM2** exhibited a slight antimicrobial activity against *A. baumannii*. The other antimicrobials showed no activity. *S. aureus* was found to be the most susceptible species (MIC range, 16–64 mg/L; MIC_50_, 32 mg/L; MIC_90_ 64 mg/L), followed by *S. epidermidis* and *Enterococcus* spp. To note, within the genus *Enterococcus*, **DM8** was more effective against *E. faecium* (MIC_50_, 32 mg/L) than *E. faecalis* (MIC_50_, 256 mg/L). It is important to highlight that the direct precursors of **DM2** and **DM8**, i.e., caffeic acid and o-coumaric acid, respectively, showed MIC values against Gram-positive species higher than 512 mg/L, suggesting that the conjugation to carvacrol was important to ameliorate the antimicrobial activity (data not shown).

Analysing the antimicrobial activity results of **DM1–11**, the following structure–activity relationships can be drawn:

1. The presence of one, two, or three substituents on the ring structure of DM derivatives influenced the antimicrobial activity. The results showed that the introduction of one, two, or three methoxy groups, enhanced the lipophilicity of the molecule (Table 1), and negatively affected the antimicrobial activity.
2. One or two hydroxyl groups on the ring structure of **DM8** and **DM2**, respectively, enhanced the hydrophilia, ameliorating the interaction only with the cell wall of Gram-positive pathogens. The uptake of **DM2** and **DM8** into Gram-positive bacteria (*S. aureus* and *epidermidis*) was more efficient than into Gram-negative bacteria due to the differences in the cell walls. It is well known that the polar nature of the outer membrane of Gram-negative bacteria results in limited passive permeability of hydrophobic drugs. Moreover, the presence of promiscuous efflux pumps can hinder the entry of antimicrobials.
3. **DM2**, having the lowest Log *P* (equal to 3.83) as compared with other DM derivatives, was found to be the most potent antimicrobial agent against *S. aureus* and *epidermidis*, which indicated that the presence of two withdrawing substituents on m- and p-position of the phenyl nucleus are important for the interaction with the bacterial cell wall.
4. Despite **DM8-10** having the same values as Log *P*, the position of the hydroxyl group in orto-, meta-, and para-position on the benzene ring of these molecules influenced the antimicrobial activity. The introduction of -OH moiety in meta- and para- in **DM9** and **DM10**, respectively, caused a drastic loss of activity as compared with **DM8**, which had the OH- in orto;
5. **DM8** was the most active compound against *E. faecium* (MIC_50%_ = 32 mg/L), suggesting that the hydroxyl group in o-position, as compared with **DM1** that avoided substituents on the phenyl nucleus, is important for the interaction with the enterococcal cell wall.

**Figure 2:**
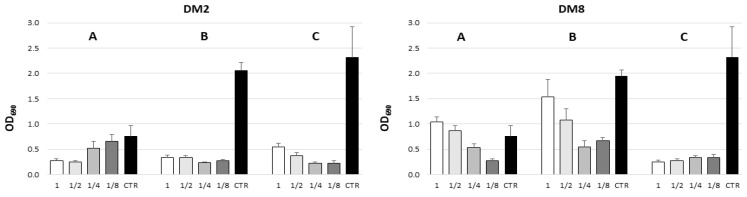
**DM2** and **DM8** effects on biofilm formation produced by: (**A**) *S. aureus* ATCC 43300; (**B**) *S. epidermidis* ATCC 35984; (**C**) *S. agalactiae* 343676. The biofilm forming ability was assessed in the presence of different concentrations of **DM2** and **DM8** (1, 1/2, 1/4, and 1/8 × MIC). CTR, untreated control. Values are expressed as mean OD_690_ ± standard deviation (SD).

In the present study, the most active cinnamic acid-based antimicrobials were also investigated as potential agents for preventing bacterial biofilm formation on infected wounds [26]. The effects of **DM2** and **DM8** were tested against two well-known biofilm producers, i.e., reference strains *S. aureus* ATCC 43300 and *S. epidermidis* ATCC 35984 (RP62A) (Figure 2). The antibiofilm activities of **DM2** and **DM8** were also assayed against *S. agalactiae* 343676, even if the compounds did not show antimicrobial activity against this species (MIC_50_ > 512 mg/L, Table 2). As the clinical isolate 343676 turned out to be a strong biofilm producer, it was included as a study model to evaluate the **DM2**/**DM8** effects on biofilm formation. The strain has been isolated from blood of a preterm infant and subsequently characterized for the virulence and adherence factors to host cells. 

Hence, **DM2** and **DM8** at their sub-MIC were evaluated against *S. aureus* ATCC 43300, *S. epidermidis* ATCC 35984 (RP62A), and *S. agalactiae* 343676 biofilm formations. The ability of **DM2** and **DM8** to reduce the biofilm formation at 24 h was examined. The results showed that **DM2** and **DM8**, at the same concentrations, caused different behaviours in inhibiting the formation of biofilm in the tested Gram-positive bacteria. The results were not encouraging that were obtained for both compounds against biofilm produced by *S. aureus,* while **DM2** was more effective than **DM8** in the treatment of biofilm caused by *S. epidermidis* ATCC 35984. The most important virulence factor of *S. epidermidis* is its aptitude to form biofilms, enabling it to adhere to a surface establishing a mucoid layer on polymer surfaces. Staphylococcal biofilm is often difficult to eradicate since it is also the source of several obstinate infections. Treatment with **DM2** resulted in significant inhibition of *S. epidermidis* biofilm formation even at low concentrations (1 to 1/8 × MIC). Considering that the single precursors of **DM2** and **DM8** showed antibiofilm activity at MIC values higher than ½ MIC (data not shown), their conjugation to carvacrol resulted in a promising strategy.

Moreover, both DM derivatives were highly effective against *S. agalactiae* 343676 biofilms, with a significant reduction in biomass, from 5 to 10 times as compared with the control (average DO_690_, 2.3). *S. agalactiae* is a Gram-positive bacterium that, albeit to a lesser extent than other species, is often present in ulcerative skin lesions [27]. Remarkably, **DM8** was the most effective in preventing the biofilm formation at all sub-MIC concentrations (up to 1/8 MIC). In *S. agalactiae*, as reported for many bacterial species, biofilm formation is pivotal to promoting colonization of the host tissues. The capacity to form biofilm is affected by environmental conditions and on the expression of specific virulence and adherence factors, such as bacterial capsule, and pili. The good activity of **DM2,** and even more so **DM8,** on biofilm formation of *S. agalactiae* could be due to a similar mechanism as that previously observed for *P. aeruginosa*, where cinnamic acid derivatives seemed to act as competitive inhibitors for the natural ligands interfering with the quorum sensing activation circuits for biofilm production [28]. Since biofilm formation plays a central role in the phenotype switch from commensal to pathogen, these results are of interest and deserve further study. 

### 2.4. Evaluation of the Wound Healing Effect of ***DM2***

ESKAPE microorganisms are the most widespread bacteria in skin infections [29]. Notably, infected cutaneous wounds such as ulcers or surgical wounds may be infested by biofilm-forming pathogens [30]. The management of wounds infected by bacteria is often difficult, and commercially available topical agents are frequently inadequate to solve the problem. Wound healing is the typical result of tissue injury that ends in the repair of damaged tissue to its normal state. In wound infection, Gram-positive bacteria, such as *Staphylococci*, are the first skin-colonizing bacteria since their optimum pH for growth is about 7. Afterwards, bacteria that are both Gram-positive and Gram-negative, such as *P. aeruginosa* and *E. faecalis*, can also be found because the pH of the wound environment changes and becomes wider. In chronic wounds, where the pH is higher than 8, anaerobes such as *Peptostreptococci* spread [31].

**Figure 3:**
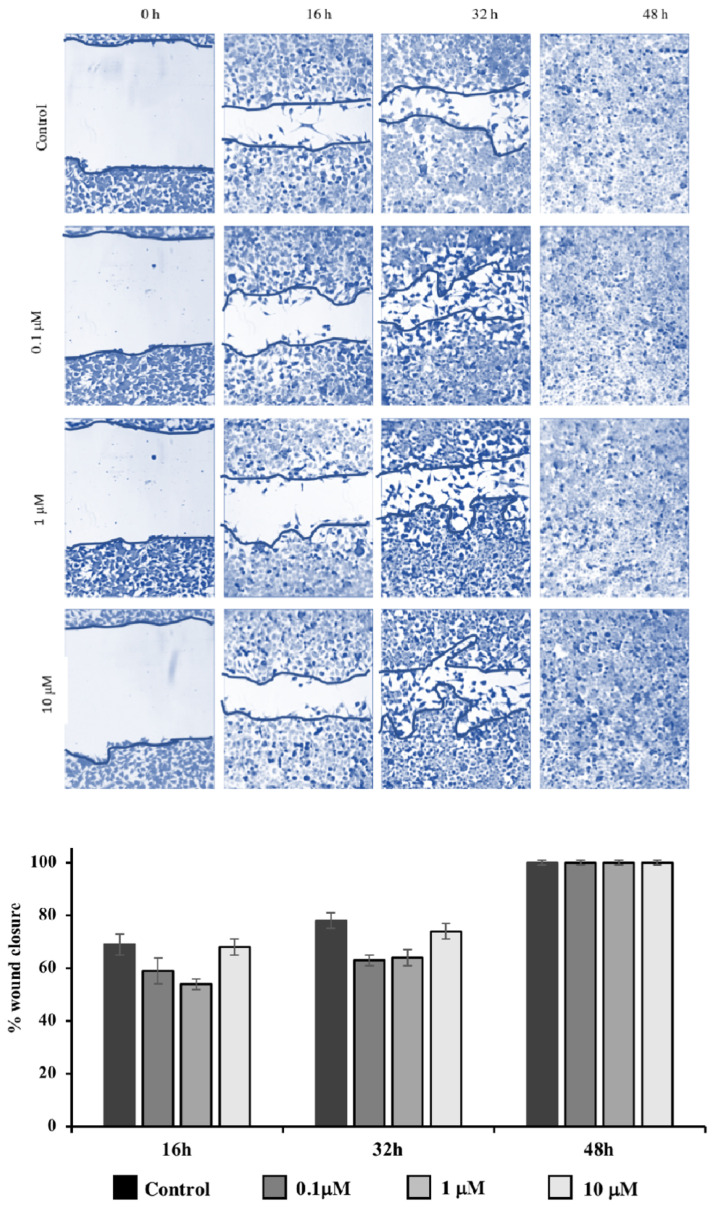
Effect of **DM2** (0.1, 1, and 10 µM) on human keratinocyte migration in the wound healing migration assay. (**Up**) Representative phase-contrast images of the wounds were taken at 0, 16, 32, and 48 h (200× magnification). **(Down**) Quantification of the percentage of closed wound area calculated by tracing the border of the wound using ImageJ software. Data represent the mean ± SD of three independent experiments.

In this study, we examined the antimicrobial effects of **DM2** on wound healing as wounds are often infected by bacteria (Figure 3). As *S. epidermidis* is often associated with the growth of biofilms that develop especially in skin chronic wounds, the influence of **DM2** exposition on wound healing was evaluated using an in vitro scratch wound assay. Wound closure was investigated using keratinocytes treated with **DM2** at 16, 32, and 48 h. The data obtained was transformed from scratch width in (pixel) to wound area in (%) (Figure 3). Measurement of the scratch width after 16 and 32 h reveals that **DM2** at the concentrations of 0.1 and 1 µM induced a slower cell migration than those observed at 10 µM. In fact, the migration velocity at 10 µM of **DM2** was comparable to untreated cells. Interestingly, complete wound closure in 48 h was observed in **DM2**-treated keratinocytes with a better healing pattern at all the concentrations used (0.1, 1.0, and 10 µM) (Figure 3). 

The wound microenvironment plays an important role in the wound healing process. The presence of biofilm-forming bacteria, humidity, pH, production of reactive oxygen species, expression of proinflammatory cytokines and metalloproteinases, and degradation of growth factors can develop a vicious inflammatory circle that further impairs healing [32]. Notably, common skin-colonizing microorganisms may be responsible for skin and wound infections [33]. The data in the literature report that 60% of chronic wounds are colonized by biofilm-forming bacteria as compared with 6% of acute wounds [34]. The role of biofilm has been confirmed in all phases of wound healing [35]. *S. aureus*, *S. epidermidis*, and *P. aeruginosa* are the most common pathogens responsible for the formation of biofilm in chronic wounds [36]. In this context, antimicrobial and antibiofilm activity of **DM2** at low concentrations could prevent wound infection development, resulting in wound healing acceleration and enhancement of rate of wound closure. In addition, the free catechol group of **DM2**, as compared with other DM derivatives, could contribute to a reduction in reactive oxygen species due to its well-known radical scavenging activity [37]. However, further studies are needed to understand the involvement of **DM2** in the healing pathway. 

## 3. Cytotoxicity Studies

**Figure 4:**
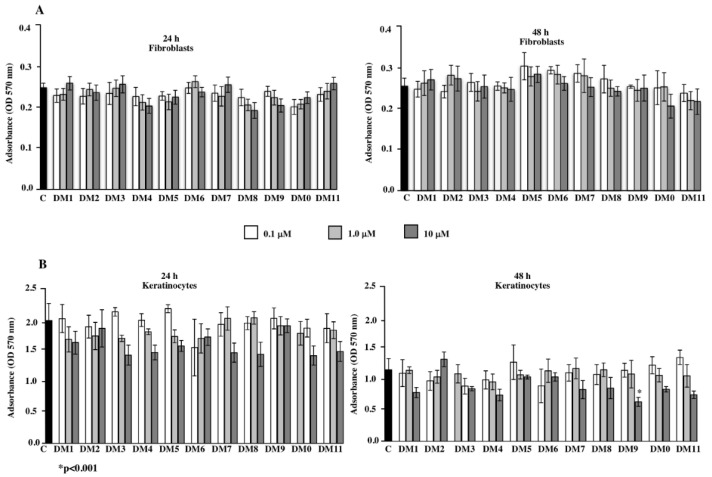
Effects of **DM1–11** (0.1, 1.0, and 10 µM) on human fibroblasts (panel **A**) and keratinocytes (panel **B**) using MTT assay. The results for the **DM1–11** compounds are expressed as optical density as compared with the control. The values represent the mean ± SD of three independent experiments performed in eightfold for each sample. Differences vs. control, * *p* < 0.001.

As cinnamic acid-based antimicrobials have been designed as potential antimicrobial for topic application, their cytotoxicity has been determined on both human fibroblasts and keratinocytes cell lines. The results presented in Figure 4 show that almost all **DM1–11** did not affect fibroblasts viability after 24 h at the tested concentrations (0.1, 1.0, and 10 µM). Slight but not significant decreases in cell viability were observed for **DM4** and **DM8**,**9** at 10 µM (*p* > 0.05). As compared with controls, no significant changes in cell number were observed when fibroblasts were treated with **DM1–11** for 48 h (Figure 4, Panel A). 

The MTT test performed on keratinocytes showed that none of 11 compounds produced a significant modification in cell number after 24 h of treatment at any tested concentration (*p* > 0.05). However, after 48 h of treatment with **DM9** (10 µM) a significant decrease in cell viability was observed (−46%) (*p* < 0.001) (Figure 4, Panel B). In particular, the most promising DM derivative, **DM2**, did not show cytotoxicity in cellular lines at all tested concentrations.

## 4. Materials and Methods

All reagents were provided by Sigma-Aldrich Co. (St. Louis, MO, USA). Chromatographic columns were performed on silica gel using column chromatography (Merck 60, 230–400 mesh ASTM silica gel), and co. NMR spectra were recorded with a Varian VXR-300 spectrometer (Varian Medical Systems, Inc., Paolo Alto, CA, USA). Microanalysis (C, H, N) was performed on a Carlo Erba instrument model E1110. Analyses indicated by the symbols of the elements or functions were within ±0.4% of the theoretical values. The liquid chromatograph system was an Agilent 1260 Infinity II HPLC (Agilent, Santa Clara, CA, USA) consisting of a 1260 Infinity II Quaternary Pump (model G7111A), 1260 Infinity II auto-sampler (model G7129A), a 1260 Infinity II Multicolumn Thermostat (model G7116A), and a 1260 Infinity II Diode Array Detector (model G7115A). Data were acquired and integrated using a software Agilent OpenLAB CDS LC ChemStation. The separation was performed using a Poroshell 120 EC-C18 (150 × 4.6 mm i.d., particle size 4 μm, Agilent, Santa Clara, USA), maintained at 20 °C. Samples were run using a mixture of water (A), acetonitrile (B), enriched with trifluoroacetic acid (0.1% *v*/*v*). The flow rate was 0.8 mL/min. The UV-detector was set at a length of 254 nm. The chemical structures of **DM1–11** compounds were confirmed by ^1^H- and ^13^C-NMR (NMR spectra are reported in the Supporting Information). All compounds are >95% pure by HPLC analysis (HPLC chromatograms are reported in the Supporting Information).

For the high-resolution mass spectrometry analysis, the **DM1–11** compounds were dissolved in ACN/H_2_O 80/20 with 0.1% of formic acid at 10 μg/mL and injected into the mass spectrometer through a syringe pump at a flow rate of 5 μL/min. The mass spectrometer used was a Thermo Fischer Orbitrap FusionTM TribridTM operating in MS scan in the *m*/*z* range from 80 to 500, equipped with the Orbitrap as detector type at 240,000 of mass resolution (FWHM). Except for the **DM2**, all compounds were acquired in positive ion mode.

### 4.1. Chemistry

#### 4.1.1. General Procedure for the Synthesis of **DM1**, **DM3–7**, and **DM11**

Cinnamic acid or its derivate (1 eq) was dissolved in dry DCM (2 mL) prior to the addition of carvacrol (1.1 eq), BOP (1.2 eq), and TEA (4 eq) at 0 °C [38]. Then, the mixture was stirred for 24 h at room temperature. After the evaporating of the solvent, ethyl acetate (EtOAc) was added and the solution was washed with 10% citric acid, 10% NaHCO_3_ brine, and dried over anhydrous sodium sulphate. Filtered solution was concentrated under pressure and purified on silica gel with CHCl_3_ as the eluent. The final compounds (**DM1**, **DM3–7**, and **DM11**) were obtained as oils.

**5-isopropyl-2-methylphenyl-cinnamate** (**DM1**). Yield: 55%; R_f_ = 0.91, CHCl_3_; ^1^H NMR (300 MHz, CDCl_3_) δ: 1.27 (6 H, d, *J* = 6.9 Hz), 2.20 (3H, s), 2.92 (1H, m), 6.71 (1H, d, *J* = 15.9 Hz), 6.97 (1H, s), 7.07 (1H, d, *J* = 7.5 Hz), 7.19 (1H, d, *J* = 7.5 Hz), 7,45 (3H, m), 7.61 (2H, m), 7.89 (1H, d, *J* = 15.9 Hz); ^13^C NMR (75 MHz, CDCl_3_) δ: 15.89 (CH_3_), 23.96 (2 × *C*H_3_), 33.60 (CH), 117.23 (CH), 119.84 (CH), 124.19 (CH), 127.38 (2 × CH), 128.31 (2 × CH), 129.01 (CH), 130.69 (CH), 130.92 (CH), 134.21 (C), 146.46 (C), 148.10 (CH), 149.32 (C), 165.24 (CO). Calcd for C_19_H_20_O_2_: C, 81.40; H, 7.19; O, 11.41. Found: C, 81.38; H, 7.16; O, 11.46. HR-MS (ESI) *m*/*z*: [M + H]^+^ = 281.1529.

**(E)-5-isopropyl-2-methylphenyl 3-(3,4-dimethoxyphenyl) acrylate** (**DM3**). Yield: 85%; R_f_ = 0.67, CHCl_3_; ^1^H NMR (300 MHz, CDCl_3_) δ:1.26 (6H, d, *J* = 7.2 Hz), 2.18 (3H, s), 2.89 (1H, m), 3.94 (6H, s), 6.56 (1H, d, *J* = 18 Hz), 6.91 (2H, m), 7.04 (1H, m), 7.16 (3H, m), 7.81 (1H, d, *J* = 18 Hz). ^13^C NMR (75 MHz, d_6_-DMSO) δ: 15.88 (CH_3_), 24.26 (2 × CH_3_), 33.29 (CH), 55.99 (CH_3_), 56.05 (CH_3_), 110.84 (CH), 111.88 (CH), 114.77 (CH), 120.27 (CH), 124.01 (CH), 124.23 (CH), 127.10 (C), 127.53 (C), 129.41 (CH), 147.10 (C), 148.09 (CH), 149.42 (C), 149.6 (C), 151.74 (C), 165.46 (CO). Calcd for C_21_H_24_O_4_: C, 74.09; H, 7.11; O, 18.80. Found: C, 74.06; H, 7.09; O, 18.85. HR-MS (ESI) *m*/*z*: [M + H]^+^ = 341.1739.

**(E)-5-isopropyl-2-methylphenyl 3-(benzo[d]** [1,3] **dioxol-5-yl) acrylate** (**DM4**). Yield: 52%; R_f_ = 0.77, CHCl_3_; ^1^H NMR (300 MHz, CDCl_3_) δ:1.26 (6H, d, *J* = 7.2 Hz), 2.18 (3H, s), 2.90 (1H, m), 6.04 (2H, s), 6.52 (1H, d, *J* = 15.9 Hz), 6.86 (1H, d, *J* = 7.5 Hz), 6.94 (1H, s), 7.06–7.26 (4H, m), 7.77 (1H, d, *J* = 15.6 Hz). ^13^C NMR (75 MHz, CDCl_3_) δ: 15.88 (CH_3_), 24.24 (2 × CH_3_), 33.31 (CH), 102.15 (CH_2_), 107.30 (CH), 108.99 (CH), 115.18 (CH), 120.25 (CH), 124.28 (CH), 126.06 (CH), 127.48 (C), 128.76 (C), 131.16 (CH), 146.76 (C), 148.11 (CH), 148.56 (C), 149.59 (C), 150.16 (C), 165.34 (CO). Calcd for C_20_H_20_O_4_: C, 74.06; H, 6.21; O, 19.73. Found: C, 74.04; H, 6.19; O, 19.77. HR-MS (ESI) *m*/*z*: [M + H]^+^ = 325.1429.

**(E)-5-isopropyl-2-methylphenyl 3-(2-methoxyphenyl) acrylate** (**DM5**). Yield: 60%; R_f_ = 0.70, CHCl_3_; ^1^H NMR (300 MHz, d_6_-DMSO) δ: 1.11 (6H, d, *J* = 6.9 Hz), 2.03 (3H, s), 2.70 (1H, m), 3.83 (3H, s), 6.50 (1H, d, *J* = 15.5 Hz), 6.60 (2H, m), 6.89–7.07 (4H, m), 7.64 (1H, d), 7.81 (1H, d, *J* = 15.4 Hz). ^13^C NMR (75 MHz, d_6_-DMSO) δ: 15.34 (CH_3_), 23.33 (2 × CH_3_), 33.21 (CH), 56.25 (CH_3_), 114.23 (CH), 115.51 (CH), 118.52 (CH), 120.91 (CH), 122.84 (CH), 125.71 (CH), 127.80 (C), 128.93 (C), 129.12 (CH), 135.24 (CH), 141.41 (*C*H), 147.47 (C), 150.00 (C), 159.23 (C), 164.35 (CO). Calcd for C_20_H_22_O_3_: C, 77.39; H, 7.14; O, 15.46. Found: C, 77.37; H, 7.13; O, 15.50. HR-MS (ESI) *m*/*z*: [M + H]^+^ = 311.1637.

**(E)-4-isopropyl-2-methylphenyl 3-(4-methoxyphenyl) acrylate** (**DM6**). Yield: 58%; R_f_ = 0.69, CHCl_3_; ^1^H NMR (300 MHz, CDCl_3_) δ: 1.33 (6H, d, *J* = 6.9 Hz), 2.26 (3H, s), 2.95 (1H, m), 3.86 (3H, s), 6.64 (1H, d, *J* = 15.9 Hz), 6.99 (2H, d, *J* = 6.9 Hz), 7.04 (1H, s), 7.11 (1H, d, *J* = 7.5 Hz), 7.26 (1H, m), 7.57 (2H, d, *J* = 9.0 Hz), 7.90 (1H, d, *J* = 15.6 Hz). ^13^C NMR (75 MHz, d_6_-DMSO) δ: 15.89 (CH_3_), 24.25 (2 × *C*H_3_), 33.31 (CH), 55.81 (CH_3_), 114.61 (2 × CH), 114.90 (CH), 120.28 (CH), 124.25 (CH), 126.93 (C), 127.51 (C), 131.01 (CH), 131.16 (2 × CH), 146.70 (C), 148.11 (CH), 149.62 (C), 161.93 (C), 165.40 (CO). Calcd for C_20_H_22_O_3_: C, 77.39; H, 7.14; O, 15.46. Found: C, 77.38; H, 7.12; O, 15.50. HR-MS (ESI) *m*/*z*: [M + H]^+^ = 311.1637.

**(E)-5-isopropyl-2-methylphenyl 3-(3-methoxyphenyl) acrylate** (**DM7**). Yield: 62%; R_f_ = 0.71, CHCl_3_; ^1^H NMR (300 MHz, d_6_-DMSO) δ: 1.11 (6H, d, *J* = 6.9 Hz), 2.03 (3H, s), 2.70 (1H, m), 3.34 (3H, s), 6.50 (1H, d, *J* = 15.8 Hz), 6.60 (2H, m), 6.89 (2H, m), 7.23–7.30 (2H, m), 7.52 (1H, d, *J* = 15.7 Hz). ^13^C NMR (75 MHz, d_6_-DMSO) δ: 16.05 (CH_3_), 24.44 (2 × CH_3_), 33.49 (CH), 55.64 (CH_3_), 112.91 (CH), 113.31 (CH), 116.69 (CH), 117.08 (CH), 119.97 (CH), 121.23 (CH), 121.38 (C), 130.35 (C), 130.73 (CH), 136.09 (C), 144.34 (CH), 147.41 (C), 155.60 (C), 168.04 (CO). Calcd for C_20_H_22_O_3_: C, 77.39; H, 7.14; O, 15.46. Found: C, 77.36; H, 7.15; O, 15.49. HR-MS (ESI) *m*/*z*: [M + H]^+^ = 311.1637.

**(E)-5-isopropyl-2-methylphenyl 3-(3,4,5-trimethoxyphenyl) acrylate** (**DM11**). Yield: 78%; R_f_ = 0.71, CHCl_3_; ^1^H NMR (300 MHz, CDCl_3_) δ:1.26 (6H, d, *J* = 7.2 Hz), 2.18 (3H, s), 2.90 (1H, m), 3.91 (9H, s), 6.60 (1H, d, *J* = 15.9 Hz), 6.83 (2H, s), 6.94 (1H, s), 7.06 (1H, d, *J* = 7.8 Hz), 7.19 (1H, d, *J* = 7.5 Hz), 7.78 (1H, d, *J* = 15.9 Hz). ^13^C NMR (75 MHz, CDCl_3_) δ: 15.85 (CH_3_), 23.91 (2 × *C*H_3_), 33.57 (CH), 56.18 (2 × *C*H_3_), 61.00 (CH_3_), 105.44 (2 × CH), 116.44 (CH), 119.81 (CH), 124.16 (CH), 127.33 (C), 129.68 (C), 130.90 (CH), 140.42 (C), 146.37 (C), 148.08 (CH), 149.30 (C), 153.50 (2 × C), 165.18 (CO). Calcd for C_22_H_26_O_5_: C, 71.33; H, 7.07; O, 21.60. Found: C, 71.31; H, 7.05; O, 21.64. HR-MS (ESI) *m*/*z*: [M + H]^+^ = 371.1848.

#### 4.1.2. General Procedure for the Synthesis of **DM8–10**

To a solution of the suitable cinnamic acid derivative (1 eq) in dry DMF (5 mL), carvacrol (1 eq), EDC^.^HCl (1 eq), and DMAP (0.10 eq) were added. The mixture was stirred overnight at 0 °C, and the solvent was removed under vacuum. Then, the crude residue was dissolved in CHCl_3_ and washed with 10% citric acid, 10% NaHCO_3_, water and brine and dried over anhydrous sodium sulphate. The compound was filtered and purified on silica gel using CHCl_3_ as eluent affording **DM8–10** as oils.

**(E)-5-isopropyl-2-methylphenyl 3-(2-hydroxyphenyl) acrylate** (**DM8**). Yield: 51%; R_f_ = 0.30, CHCl_3_; ^1^H NMR (300 MHz, CDCl_3_) δ: 1.26 (6H, d, *J* = 8.1 Hz), 2.18 (3H, s), 2.89 (1H, m), 6.75- 6.97 (4H, m), 7.05 (1H, d, *J* = 7.8 Hz), 7.17–7.27 (3H, m), 7.54 (1H, d, *J* = 16.5 Hz), 8.16 (1H, d, *J* = 16.5 Hz). ^13^C NMR (75 MHz, CDCl_3_) δ:15.89 (CH_3_), 23.93 (2 × *C*H_3_), 33.58 (CH), 116.41 (CH), 117.70 (CH), 119.86 (CH), 121.00 (CH), 121.47 (C), 124.14 (CH), 127.42 (CH), 129.59 (CH), 130.87 (CH), 131.77 (CH), 142.04 (CH), 148.06 (C), 149.35 (C), 155.27 (C), 166.27 (CO). Calcd for C_19_H_20_O_3_: C, 77.00; H, 6.80; O, 16.20. Found: C, 76.98; H, 6.79; O, 16.23. HR-MS (ESI) *m*/*z*: [M + H]^+^ = 297.1482.

**(E)-5-isopropyl-2-methylphenyl 3-(3-hydroxyphenyl) acrylate** (**DM9**). Yield: 32%; R_f_ = 0.16, CHCl_3_; ^1^H NMR (300 MHz, CDCl_3_) δ: 1.25 (6H, d, *J* = 6.9 Hz), 2.18 (3H, s), 2.89 (1H, m), 6.05 (1H, br d), 6.56–7.46 (8H, m), 7.85 (1H, d, *J* = 15.6 Hz). ^13^C NMR (75 MHz, CDCl_3_) δ:15.85 (CH_3_), 23.94 (2 × *C*H_3_), 33.58 (CH), 114.70 (CH), 116.97 (CH), 117.95 (CH), 118.19 (CH), 121.18 (CH), 123.90 (CH), 127.30 (C), 130.12 (CH), 130.17 (CH), 135.57 (C), 146.10 (C), 147.07 (CH), 151.18 (C), 156.39 (C), 165.33 (CO). Calcd for C_19_H_20_O_3_: C, 77.00; H, 6.80; O, 16.20. Found: C, 76.99; H, 6.78; O, 16.23. HR-MS (ESI) *m*/*z*: [M + H]^+^ = 297.1484.

**(E)-5-isopropyl-2-methylphenyl 3-(4-hydroxyphenyl) acrylate** (**DM10**). Yield: 33%; R_f_ = 0.21, CHCl_3_; ^1^H NMR (300 MHz, CDCl_3_) δ: 0.91 (3H, d, *J* = 6.9 Hz), 1.23 (3H, d, *J* = 6.6 Hz), 2.33 (3H, s), 2.93 (1H, m), 4.50 (1H, br t), 6.69 (3H, d, *J* = 8.7 Hz), 6.87 (4H, d, *J* = 8.1 Hz), 7.05 (1H, br d), 7.17 (1H, d, *J* = 16.0 Hz). ^13^C NMR (75 MHz, CDCl_3_) δ: 15.81 (CH_3_), 23.21 (CH_3_), 24.26 (CH_3_), 36.68 (CH), 115.84 (CH), 121.31 (2 × *C*H), 122.16 (CH), 123.84 (CH), 128.25 (CH), 130.43 (2 × CH), 132.66 (C), 135.4 (C), 144.83 (CH), 146.10 (C), 150.12 (C), 154.76 (C), 167.96 (CO). Calcd for C_19_H_20_O_3_: C, 77.00; H, 6.80; O, 16.20. Found: C, 76.98; H, 6.78; O, 16.24. HR-MS (ESI) *m*/*z*: [M + H]^+^ = 297.1482.

#### 4.1.3. Synthesis of **DM2**

To the solution of caffeic acid (1 eq) in 15 mL of 1M NaOH, 2 mL of acetic anhydride was added, and the reaction mixture was stirred for 30 min at 0 °C. The impure solid was filtered using vacuum filtration, and then washed with ice-cold distilled water. The crude residue was dissolved in 15 mL of boiling ethanol to recrystallized. Diacetyl caffeic acid (**12**) was dissolved in DMF (2 mL) and 5 mL of SOCl_2_ was added to the solution. The mixture reaction was heated at reflux for 4 h. The residue was dissolved in 4 mL of dry DCM and pyridine (1 mL) and carvacrol (1.2 eq) was slowly added. The mixture reaction was stirred overnight at room temperature. The diacetyl-caffeoyl derivative **13** was dissolved in dry CH_2_Cl_2_ (2 mL), and then MeOH (4 mL) was added. Guanidinium hydrochloride (3.25 eq) and TEA (9.75 eq) were added to the resulting solution, and after 2 h, the reaction mixture was evaporated. After washed with AcOEt/H_2_O, the organic layers were dried and concentrated to give pure **DM2** as oil.

**Diacetyl caffeic acid (12).** Yield: 65%; ^1^H NMR (300 MHz, d_6_-DMSO) δ: 2.26 (6H, d, *J* = 7.2 Hz), 3.34 (3H, s), 6.54 (1H, d, *J* = 15.9 Hz), 7.30 (1H, d, *J* = 15.8 Hz), 7.52- 7.65 (3H, m). 

**(E)-4-(3-(5-isopropyl-2-methylphenoxy)-3-oxoprop-1-en-1-yl)-1,2-phenylene diacetate** (**13)**. Yield: 79%; R_f_ = 0.39, CHCl_3_; ^1^H NMR (300 MHz, CDCl_3_) δ: 1.26 (6H, d, *J* = 7.2 Hz), 2.17 (3H, s), 2.33 (6H, d, *J* = 3.0 Hz), 2.90 (1H, m), 6.64 (1H, d, *J* = 15.9 Hz), 6.94 (1H, s), 7.03 (1H, d, *J* = 7.8 Hz), 7.06 (1H, d, *J* = 7.5 Hz), 7.25 (1H, d, *J* = 8.1 Hz), 7.44 (2H, t), 7.79 (1H, d, *J* = 15.9 Hz). ^13^C NMR (75 MHz, CDCl_3_) δ: 15.85 (CH_3_), 20.71 (2 × CH_3_), 23.94 (2 × CH_3_), 33.58 (CH), 118.39 (CH), 119.76 (CH), 122.94 (CH), 124.07 (CH), 124.23 (CH), 126.67 (C), 127.28 (C), 130.92 (CH), 133.05 (CH), 142.47 (C), 143.76 (CH), 144.45 (C), 148.11 (CH), 149.19 (C), 164.83 (C), 168.02 (C), 168.13 (CO). Calcd for C_23_H_24_O_6_: C, 69.68; H, 6.10; O, 24.22. Found: C, 69.66; H, 6.08; O, 24.26.

**(E)-5-isopropyl-2-methylphenyl 3-(3,4-dihydroxyphenyl) acrylate** (**DM2**). Yield: 59%; R_f_ = 0.21, CHCl_3_/CH_3_OH (9:1); ^1^H NMR (300 MHz, d_6_-DMSO) δ: 1.12 (6H, d, *J* = 6.9 Hz), 2.03 (3H, s), 2.68 (1H, m), 3.30 (2H, br), 6.14 (1H, d, *J* = 15.9 Hz), 6.51 (2H, d, *J* = 7.5 Hz), 6.60 (1H, s), 6.73 (1H, d, *J* = 8.1 Hz), 6.89–7.0 (2H, m), 7.39 (1H, d, *J* = 15.9 Hz). ^13^C NMR (75 MHz, d_6_-DMSO) δ: 16.05 (CH_3_), 24.4 (2 × *C*H_3_), 33.5 (CH), 112.9 (CH), 115.0 (CH), 115.5 (CH), 116.2 (CH), 117.1 (CH), 121.4 (CH), 121.6 (CH), 126.1 (C), 130.7 (CH), 145.0 (C), 145.98 (C), 147.4 (C), 148.6 (CH), 155.6 (C), 168.4 (CO). Calcd for C_19_H_20_O_4_: C, 73.06; H, 6.45; O, 20.49. Found: C, 73.04; H, 6.43; O, 20.53. HR-MS (ESI) *m*/*z*: [M − H]^+^ = 311.1648.

### 4.2. In Silico Skin Permeability Prediction Analysis

The skin permeability prediction analysis of the **DM1–11** compounds were determined with the aid of SwissADME, an online ADME prediction server at the address http://www.swissadme.ch/, (accessed on 23 August 2021). After the insertion of the chemical structure into the database, the program automatically calculated the Log *Kp*, a prediction of drug skin permeation.

### 4.3. Bacterial Strains

A collection of 76 Gram-positive and -negative clinical strains was used (the number of strains for each species is reported in Table 2). It included the following reference American Type Culture Collection (ATCC) strains: *Staphylococcus aureus* ATCC 29213 and ATCC 43300, *Staphylococcus epidermidis* ATCC 35984 (RP62A), *Enterococcus faecalis* ATCC 29212, *Streptococcus agalactiae* ATCC BAA-611 (2603V/R), *Escherichia coli* ATCC 25922 and ATCC 35218, *Klebsiella pneumoniae* ATCC 700603, *Pseudomonas aeruginosa* ATCC 27853, and *Acinetobacter baumannii* ATCC 19606. 

### 4.4. Antimicrobial Activity

Stored aliquots of the **DM1–11** compounds were dissolved in DMSO just before use. Carvacrol was dissolved in 95% ethanol and stored at −20 ° C. The MIC values of **DM1–11** and carvacrol towards all strains were determined by standard broth microdilution method, according to CLSI guidelines. All MICs were performed using cation-adjusted Mueller–Hinton broth (CAMHB) for non-fastidious organisms, while CAMHB supplemented with lysed horse blood (2.5% to 5% *v*/*v*) (CAMHB-LHB) was used for *Streptococcus* spp. Suitable positive and negative growth controls were included in all experiments, including CAMHB_DMSO_ and CAMHB_EtOH_ to check the viability of the cultures in different solvents. Microplates were incubated at 37 °C for 24 h. The MIC was defined as the lowest compound concentration that yielded no visible microorganism growth. All experiments were performed in triplicate.

### 4.5. Antibiofilm Activity

The biofilm formation assay was performed, as previously described [39], using three well-known biofilm-producing reference strains (*S. aureus* ATCC 43300, *S. epidermidis* ATCC 35984, and *P. aeruginosa* ATCC 27853) and one *S. agalactiae* hypervirulent clinical strain. Overnight cultures grown in Tryptic Soy Broth (Oxoid, Basingstoke, UK) supplemented with 1% glucose (TSB-G) were harvested by centrifugation and adjusted to an OD_650_ of 0.1. Then, 96-well polystyrene flat bottom microtiter plates (Falcon, Becton Dickinson Labware) were inoculated with 0.2 mL aliquots of the adjusted bacterial suspension and incubated at 37 °C for 24 h. To estimate biomass, unattached cells were gently aspirated and discarded, and adherent cells were washed three times in phosphate-buffered saline (PBS), dried for 1 h at 60 °C, and stained with Hucker’s crystal violet (CV) solution. After washing twice with PBS, wells were inoculated with 95% ethanol to dissolve the bound dye by shaking for 10 min. Absorbance was measured at 690 nm using a Multiscan Ascent apparatus (Thermo Scientific, Waltham, MA, USA). The optical density (OD) cut-off (ODc) was defined as three standard deviations above the mean OD of the negative control [40]. The biofilm forming ability was assessed in the presence of different concentrations of **DM2** and **DM8** (1, 1/2, 1/4, and 1/8 × MIC). Briefly, overnight bacterial suspensions were prepared to yield a final inoculum of ∼2 × 10^8^ CFU/mL. Then, 0.1 mL aliquots of adjusted bacterial suspensions were transferred to each well containing 0.1 mL of TSB-G with different concentrations of substances and the microplates were incubated at 37 °C for 24 h. After incubation, biomass was estimated as described above (CV assay). The assay was performed in triplicate. The results are shown as means ± standard deviations (SDs) of two independent experiments.

### 4.6. Cell Cultures

Human fibroblasts CRL-2522™ (ATCC, American Type Culture Collection) and human keratinocytes BSCL143 (I.Z.S.L.E.R., Istituto Zooprofilattico Sperimentale della Lombardia e dell’Emilia Romagna, Brescia, Italy) were used in this study. The cell lines were both cultured in a complete medium, namely Dulbecco’s modified Eagle’s medium (DMEM, EuroClone, Pero, MI, Italy), supplemented with 10% and 7% fetal bovine serum (FBS, EuroClone, Pero, MI, Italy), respectively, 25 μg/mL amphotericin B as antimycotic, penicillin (100 U/mL), and streptomycin (100 mg/mL) and incubated at 37 °C in a humidified 5% CO_2_ atmosphere. Upon 80% confluence, cells were detached with trypsin 0.25% in EDTA (Gibco, Paisley, UK). After 10 min of trypsinization, complete medium was added to inactivate trypsin and cells were centrifugated for 10 min at 800× *g*. Cell pellets were re-seeded in apposite plates and used for cytotoxic and scratch tests. Except for **DM2** that was dissolved in sterile Dulbecco’s phosphate buffered saline (PBS), **DM1** and **DM3–11** compounds were dissolved in DMSO and stored at −20 °C in the dark. The mixtures were diluted in DMEM just before use. All the solutions were filtered on 0.22 µm (EuroClone, Pero, MI, Italy). 

### 4.7. Wound Scratch Assay

To investigate the effect of **DM2** on keratinocyte migration, cells were plated into 6-well flat bottom microtiter plates (Thermo Fisher Scientific, Waltham, MA, USA), cultured in DMEM supplemented with 7% FBS and incubated at 37 °C in a humidified 5% CO_2_ atmosphere. At about 90% confluence, medium was removed, and the adherent cell layer was scratched using a sterile P-200 pipette tip, as described elsewhere [41]. Cellular debris was gently removed with PBS washing. Then, monolayers were exposed to different concentrations of **DM2** (0.1, 1.0, and 10 µM) and cells were grown at 37 °C in humidified 5%, CO_2_ atmosphere. Control cells received only fresh DMEM. At 0, 16, 32, and 48 h after treatment, the monolayers were washed twice with PBS, fixed with 4% paraformaldehyde at 4 °C for 30 min, and rinsed with PBS. Cells were stained with 1% crystal violet solution for 10 min at room temperature. Then, cells were washed three times in PBS, and the images were captured at 0, 16, 32, and 48 h using a conventional phase-contrast microscope (Olympus, Tokyo, Japan). The wound closure areas were calculated by ImageJ. Photographs at 200× magnification provided migration and morphology profiles.

### 4.8. MTT Viability Assay

The MTT assay was applied to evaluate the effects of **DM1–11** in proliferation and viability of human fibroblasts and keratinocytes cell lines. Cells were seeded at the concentration of 1 × 10^4^ cells/well in 96-well plates (Euroclone, Pero, MI, Italy) with 200 µL of DMEM and incubated at 37 °C in a humidified 5%, CO_2_ atmosphere. After 24 h, cells were treated with different concentrations of **DM1–11** (0.1, 1.0, and 10 µM) for 24 h and 48 h. The untreated cells (control) received only fresh medium or fresh medium plus DMSO (0.01% as final concentration). Cell viability was performed by 3-(4,5-dimethylthiazol-2-yl)-2,5-diphenyl-tetrazolium bromide (MTT) assay (Sigma Chemical Co., St. Louis, MO, USA), as previously described with some modification [42]. At the end of the treatment period, the supernatant was removed, and monolayer was washed by PBS. Then, 10 µL of MTT reagent (stock solution 5 mg/mL) were added in fresh medium (100 µL/well) and plates were incubated at 37 °C for 4 h. The formazan crystals were dissolved in 100 μL DMSO and the absorbance of each well was evaluated spectrophotometrically at 570 and 620 nm using an automatic microplate spectrophotometer reader (Biorad model 680 XR, CA, USA). The amount of color produced was directly proportional to the number of viable cells. Data represent the means ± SDs of three independent experiments each performed in sextuplicate. 

### 4.9. Statistical Analysis

The figures report the means ± SDs (standard deviations) of three independent experiments performed in sextuplicate for each compound. One-way analysis of variance (ANOVA) was performed using GraphPad Prism 5.01 software (Prism, CA, USA). *p*-values of <0.05 were statistically significant.

## 5. Conclusions

The treatment of ESKAPE-related skin wound infections is a huge challenge. The development of novel antimicrobial drugs that can contrast and/or eradicate the biofilm formed by these bacteria in infected wounds is urgently needed. Notably, targeting biofilm formation would be one such valuable strategy to counteract the biofilm-mediated problems encountered in clinical settings. In the search for new pharmacologically active compounds against antimicrobial resistance (AMR) due to ESKAPE pathogens, cinnamic acid-based antimicrobials are valuable and promising compounds with great potential for development into drugs. In this study, we investigated the antimicrobial, antibiofilm, and wound healing properties of novel cinnamic acid-based antimicrobials. Our findings collectively demonstrated that **DM2**, thanks to its antimicrobial properties against Gram-positive bacteria and antibiofilm activity against *S. epidermidis*, could be proposed as a safe wound healing topical agent for the treatment of skin wound infections. However, close investigations into the study on the mechanism involved in biofilm inhibition are currently being pursue in our laboratory. Further in vivo studies are required to understand the involvement of **DM2,** especially during the wound healing process.

## Figures and Tables

**Table 1:** In silico skin permeability values of **DM1–11**.

Compound	MW ^a^	Log *P* ^b^	Log *Kp* ^c^
**DM1**	280.26	4.60	−4.28
**DM2**	312.36	3.83	−5.01
**DM3**	340.41	4.56	−4.69
**DM4**	324.37	4.38	−4.69
**DM5**	310.39	4.61	−4.49
**DM6**	310.39	4.62	−4.49
**DM7**	310.39	4.61	−4.49
**DM8**	296.36	4.20	−4.64
**DM9**	296.36	4.22	−4.64
**DM10**	296.36	4.21	−4.64
**DM11**	370.44	4.57	−4.90

^a^ MW, molecular weight; ^b^ Log *P*, log of octanol to water partition coefficient; ^c^ Log *Kp*, log of skin permeation coefficient (cm/h).

**Table 2:** Antibacterial activities of selected cinnamic acid-based antimicrobials against a wide panel of pathogens.

Strain (n) ^a^	MIC (mg/L) ^b^	DM2	DM8	DM9	CAR
*S. aureus* (6)	range	16–64	16–512	256–>512	128–256
	50%	32	64	>512	256
	90%	64	256	>512	256
*S. epidermidis* (6)	range	64–128	256	>512	256
	50%	128	>256	>512	256
	90%	128	>256	>512	256
*E. faecalis* (10)	range	64–512	32–>512	512–>512	128–512
	50%	256	256	>512	256
	90%	512	>512	>512	256
*E. faecium* (10)	range	32–512	16–512	512->512	256
	50%	128	32	>512	256
	90%	256	512	>512	256
*S. pyogenes* (6)	range	128–>512	512–>512	>512	64–256
	50%	512	>512	>512	128
	90%	512	>512	>512	256
*S. agalactiae* (10)	range	512–>512	512–>512	512–>512	256–512
	50%	>512	>512	>512	256
	90%	>512	>512	>512	512
*E. coli* (6)	range	>512	>512	>512	256
	50%	>512	>512	>512	256
	90%	>512	>512	>512	256
*K. pneumoniae* (6)	range	512–>512	>512	>512	512
	50%	>512	>512	>512	512
	90%	>512	>512	>512	512
*P. aeruginosa* (6)	range	512–>512	>512	>512	512–>512
	50%	>512	>512	>512	>512
	90%	>512	>512	>512	>512
*A. baumannii* (10)	range	256–>512	>512	>512	64–256
	50%	512	>512	>512	128
	90%	>512	>512	>512	256
*Enterobacter* spp. (6)	range	512	>512	>512	512
	50%	512	>512	>512	512
	90%	512	>512	>512	512

**^a^** Including ATCC reference strains (n. 12); **^b^** 50% and 90%, MICs at which 50% and 90% of isolates are inhibited, respectively.

## Data Availability

Data is contained in the article and Supplementary Materials.

## Supplementary Materials

The following supporting information can be downloaded at: https://www.mdpi.com/article/10.3390/ph15020228/s1, HPLC chromatograms and ^1^H-, ^13^C-NMR, and HR-MS spectra of **DM1–11**.
