# Supplementary material for: Synthesis and Biological Evaluation of Novel Cinnamic Acid-Based Antimicrobials
Source: Pharmaceuticals (Basel). 2022 Feb 15;15(2):228. doi: 10.3390/ph15020228 (PMC8878811; doi:10.3390/ph15020228)
Supplement: Supplementary file 1 [file pharmaceuticals-15-00228-s001.zip › pharmaceuticals-1586244-supplementary.pdf]

Synthesis and biological evaluation of novel cinnamic acid-based antimicrobials

Marina Mingoia[1], Carmela Conte[2], Annalisa Di Rienzo[3], Marilisa Pia Dimmito[3], Lorella Marinucci[4], Gloria Magi[1], Hasan Turkez[5], Maria Concetta Cufaro[3,6], Piero Del Boccio[3,6], Antonio Di Stefano[3], and Ivana Cacciatore,[3,*]

1 Department of Biomedical Sciences and Public Health, Polytechnic University of Marche, Medical School, 60121 Ancona, Italy
2 Department of Pharmaceutical Sciences, University of Perugia, Via Fabretti, 48, 06123 Perugia, Italy
3 Department of Pharmacy, University "G. d'Annunzio" of Chieti-Pescara, via dei Vestini 31, 66100 Chieti Scalo, Italy
4 Department of Medicine and Surgery, University of Perugia, S. Andrea Delle Fratte, 06156 Perugia, Italy
5 Faculty of Science, Basic Medical Sciences, Medical Biology Department, Ataturk University 25240 Yakutiye, Erzurum, Turkey
6 Center for Advanced Studies and Technology (CAST), University "G. d'Annunzio" of Chieti-Pescara, via dei Vestini 31, 66100 Chieti Scalo, Italy

* Correspondence: ivana.cacciatore@unich.it; Tel.: +39 871 355 44 75

Supplementary material

HPLC CHROMATOGRAMS OF DM1-11

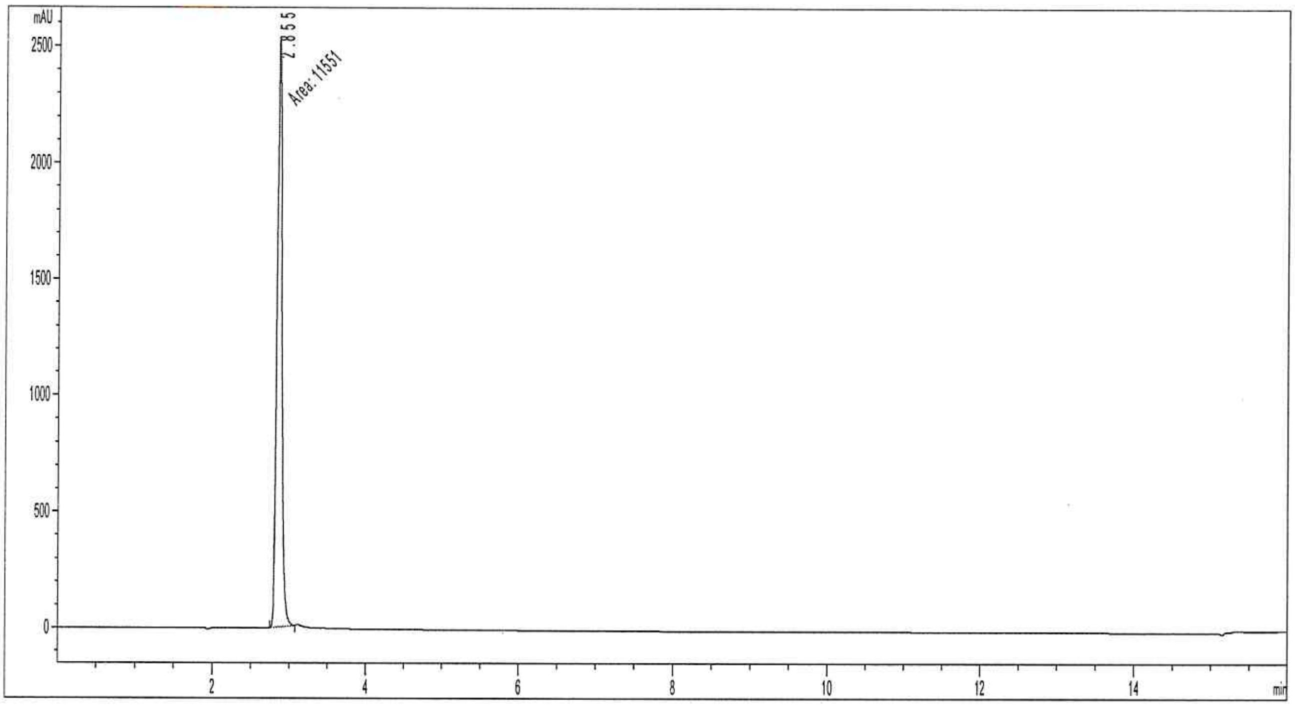

Compound	Retention time	Area%
DM1	2.855	100

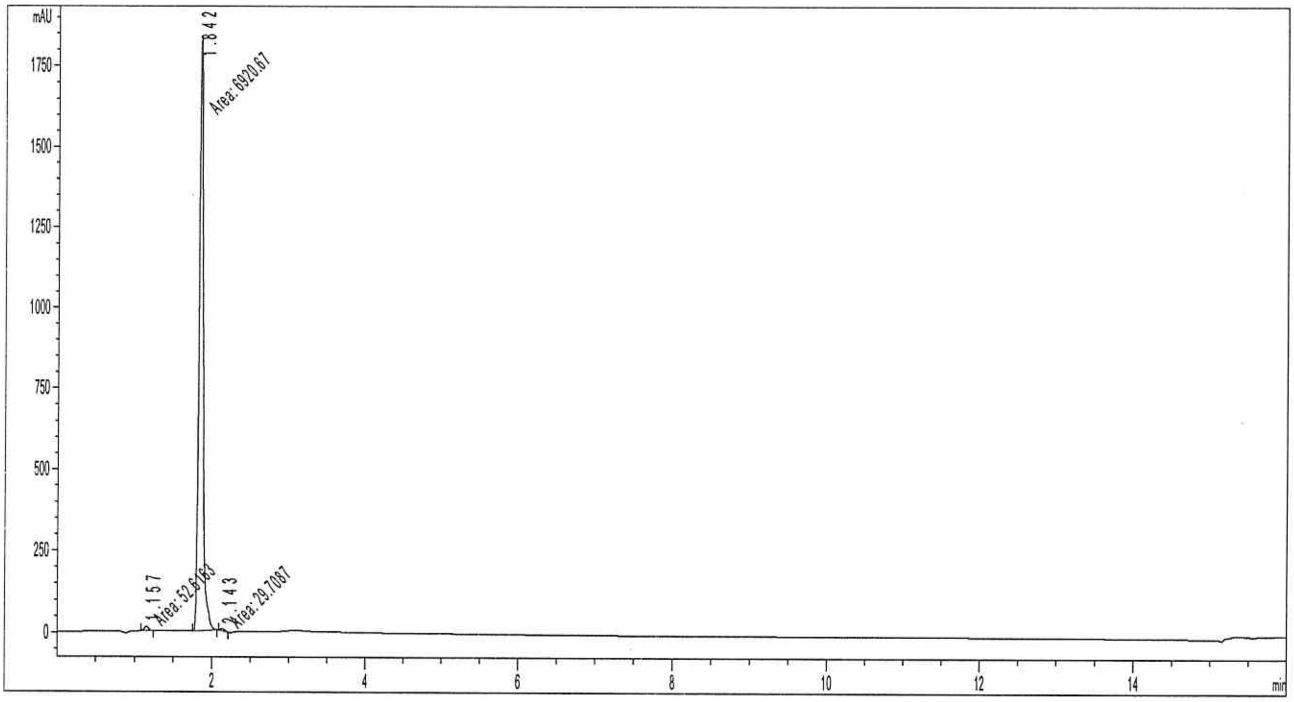

Compound	Retention time	Area%
	1.157	0.751
DM2	1.842	98.824
	2.143	0.424

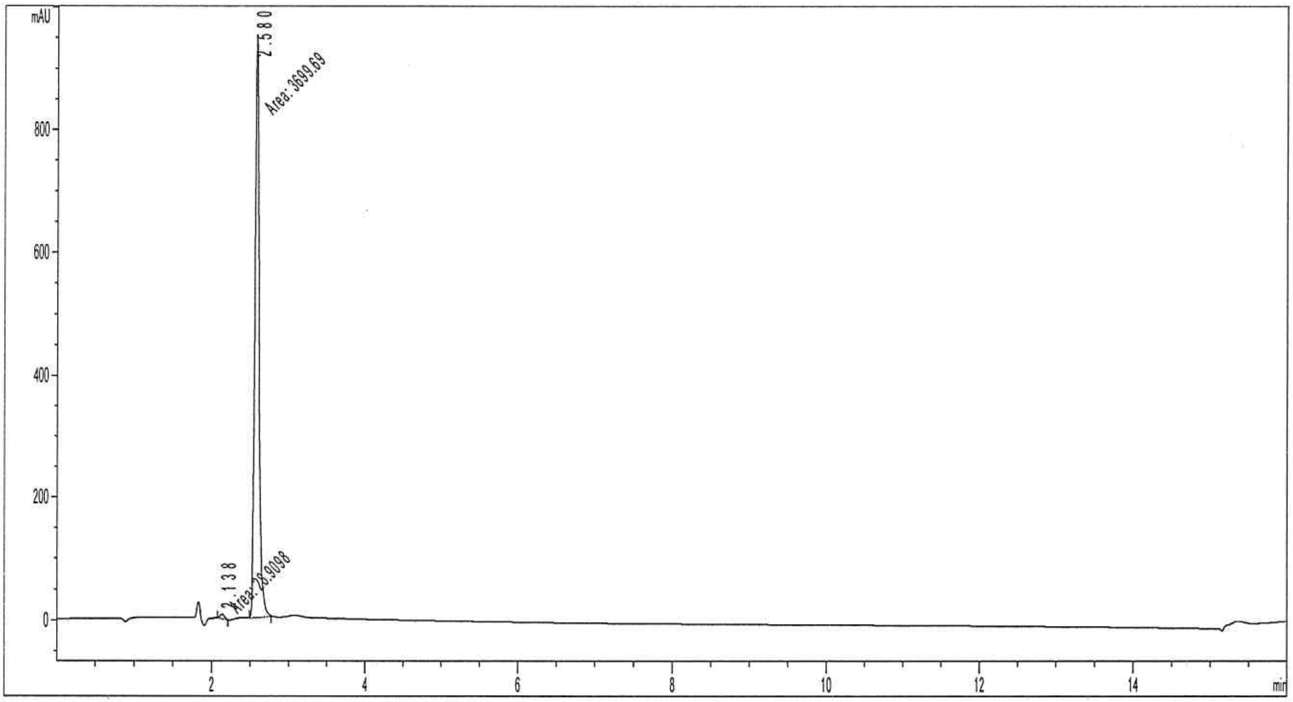

Compound	Retention time	Area%
	2.138	0.775
DM3	2.58	99.225

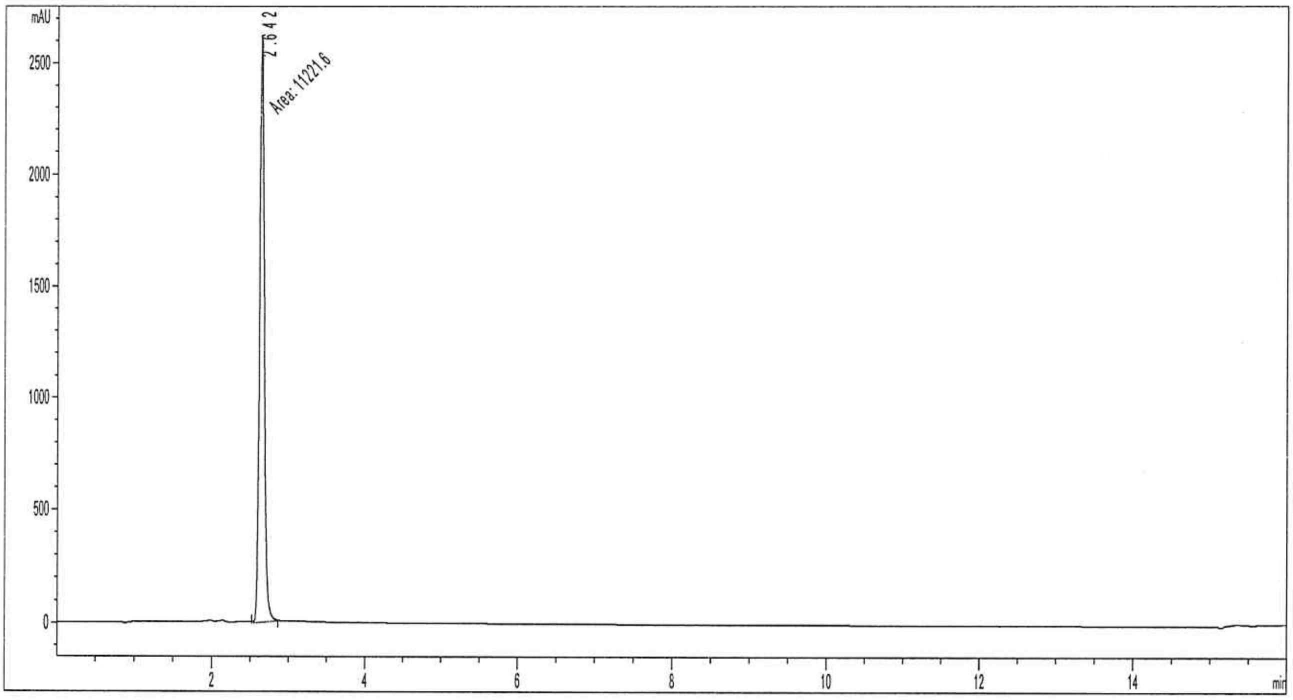

Compound	Retention time	Area%
DM4	2.642	100

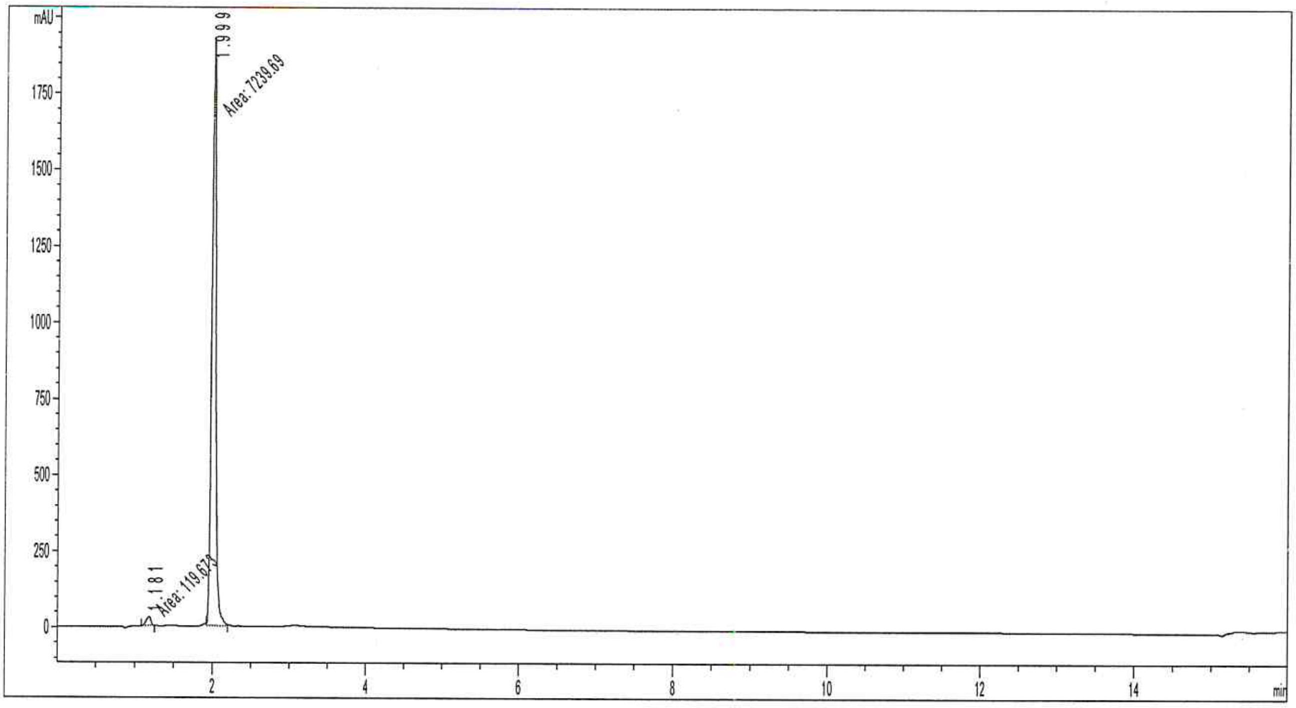

Compound	Retention time	Area%
	1.181	1.626
**DM5**	1.999	98.374

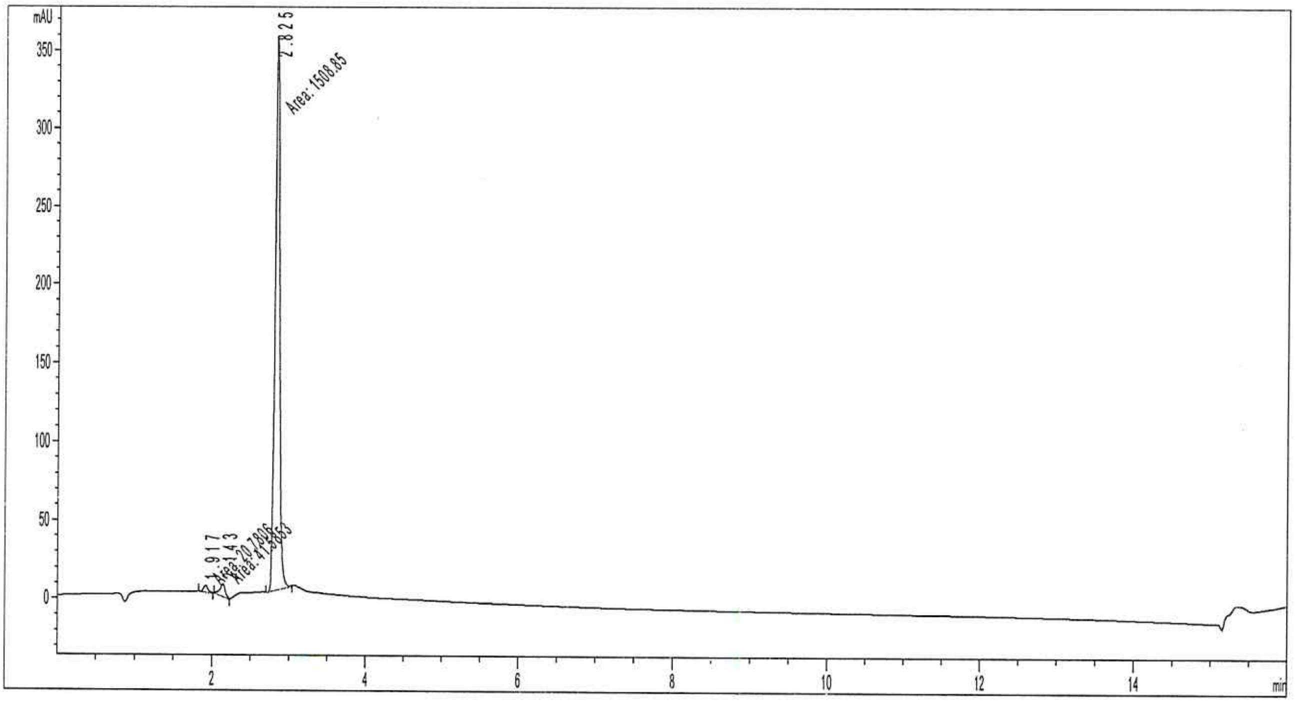

Compound	Retention time	Area%
	1.917	1.323
	2.143	2.647
**DM6**	2.825	96.031

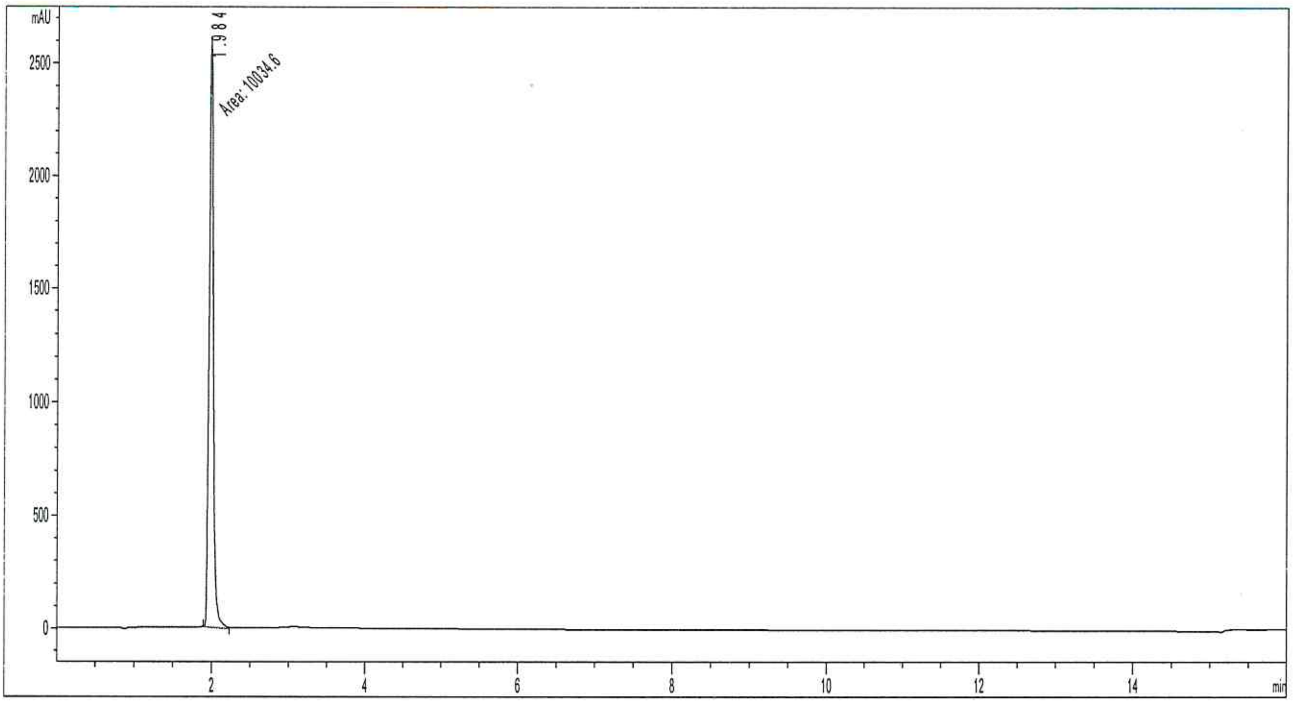

Compound	Retention time	Area%
DM7	1.984	100

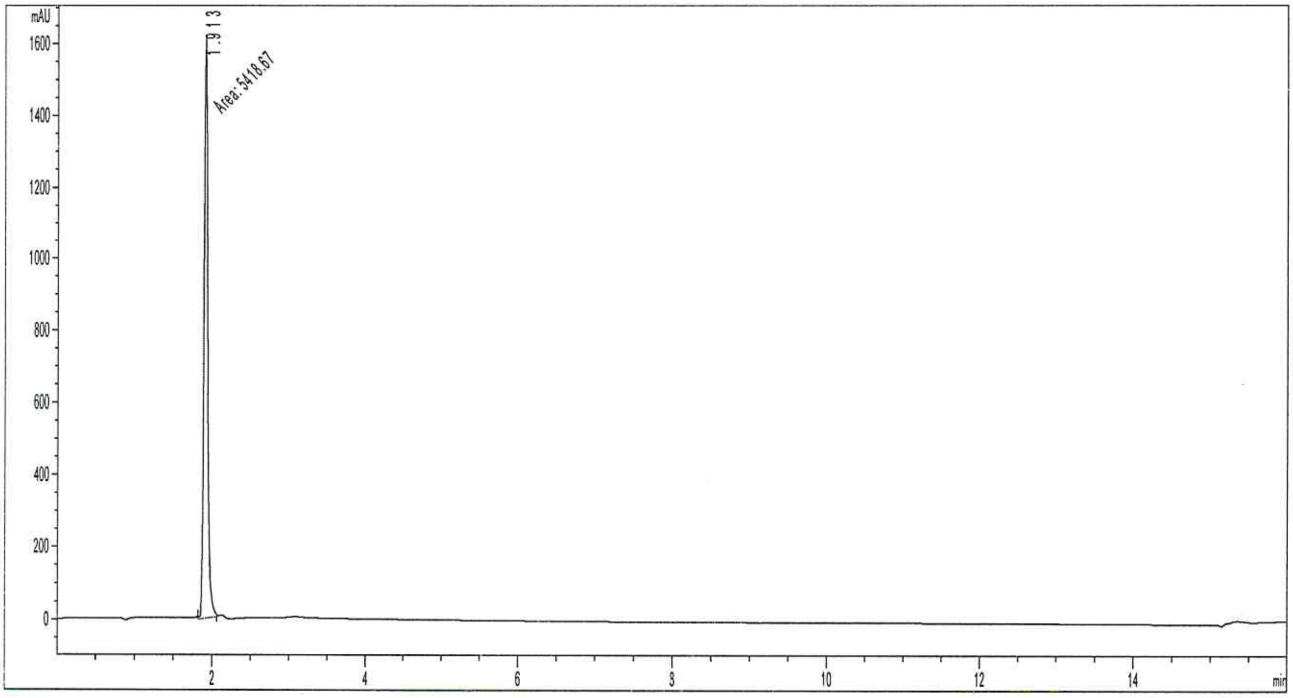

Compound	Retention time	Area%
DM8	1.913	100

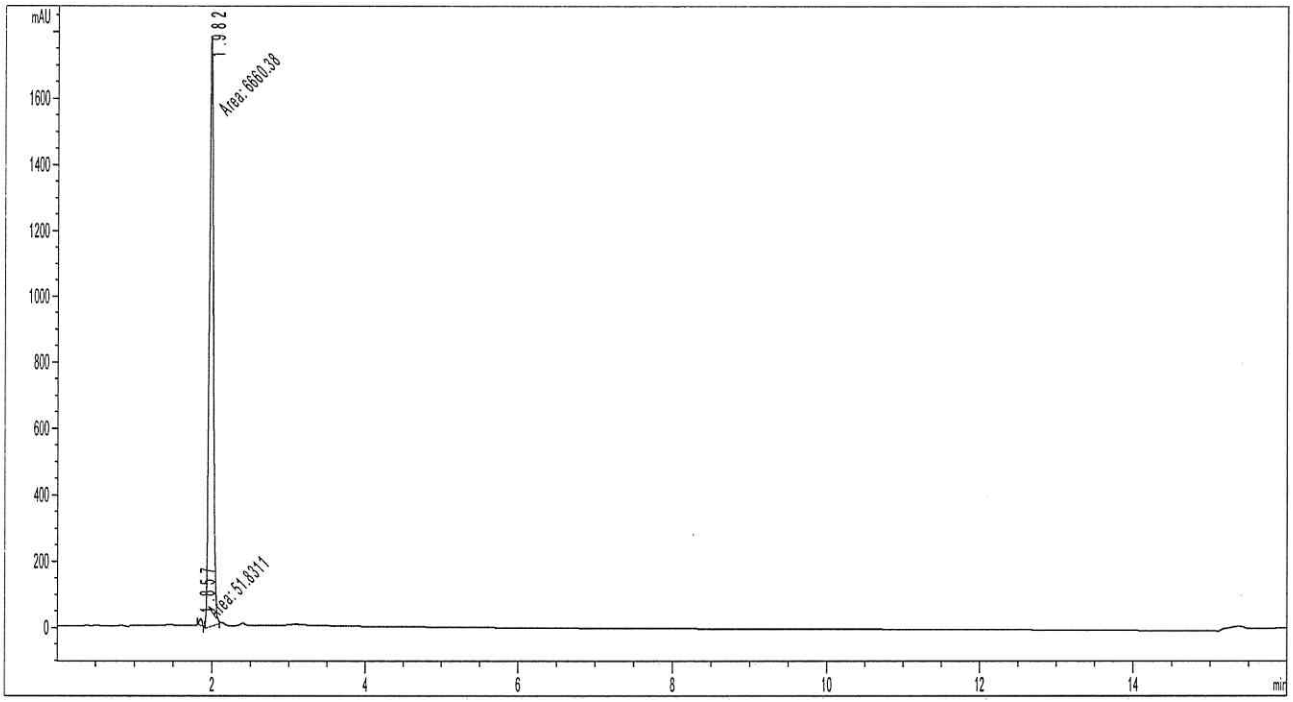

Compound	Retention time	Area%
	1.857	0.772
**DM9**	1.982	99.228

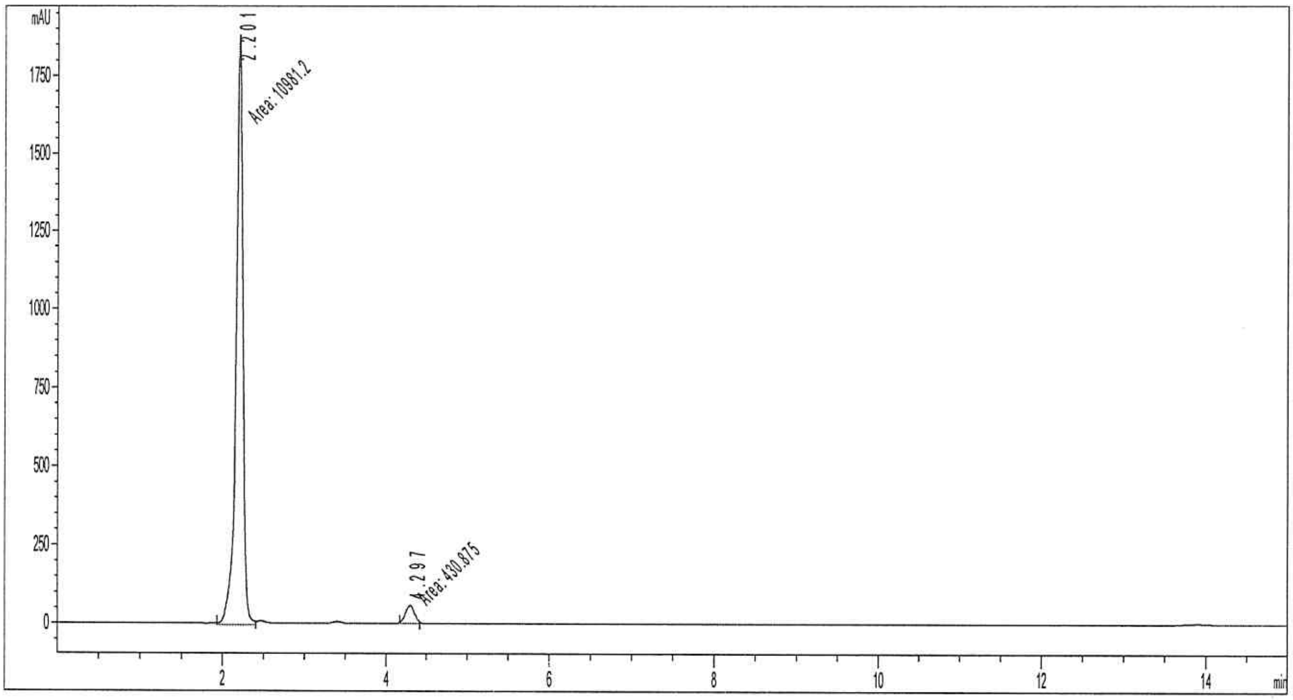

Compound	Retention time	Area%
**DM10**	2.201	96.224
	4.297	3.776

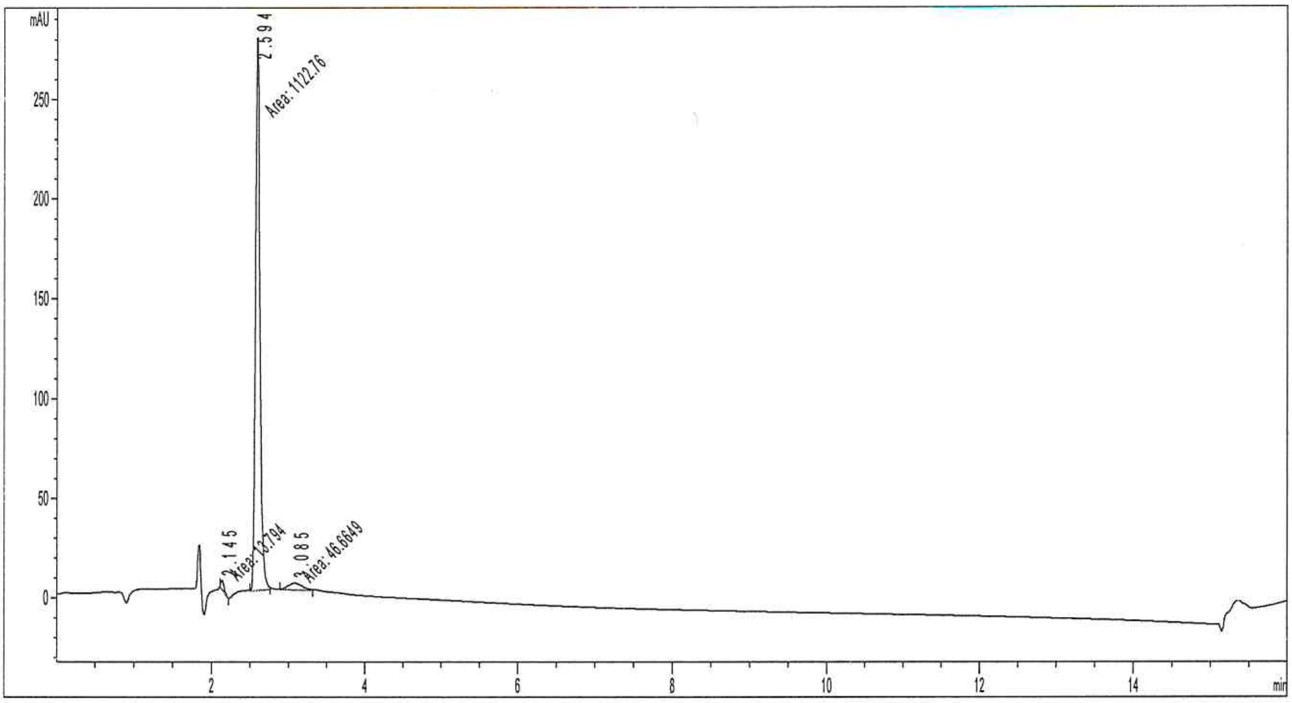

Compound	Retention time	Area%
	2.145	1.166
**DM11**	2.594	94.890
	3.085	3.944

^{1}H- AND ^{13}C-NMR SPECTRA OF **DM1-11**

DH1

Gradient Shimming

Sample Name:

Data Collected on:
m300-mercury300
Archive directory:
/export/home/chempack/vnmrsys/data
Sample directory:

FidFile: PROTON

Pulse Sequence: PROTON (s2pul)
Solvent: cdcl3
Data collected on: Jul 8 2019

Operator: caccia

Relax. delay 1.000 sec
Pulse 45.0 degrees
Acq. time 3.411 sec
Width 4803.1 Hz
16 repetitions
OBSERVE H1, 300.1976543 MHz
DATA PROCESSING
FT size 32768
Total time 1 min 13 sec

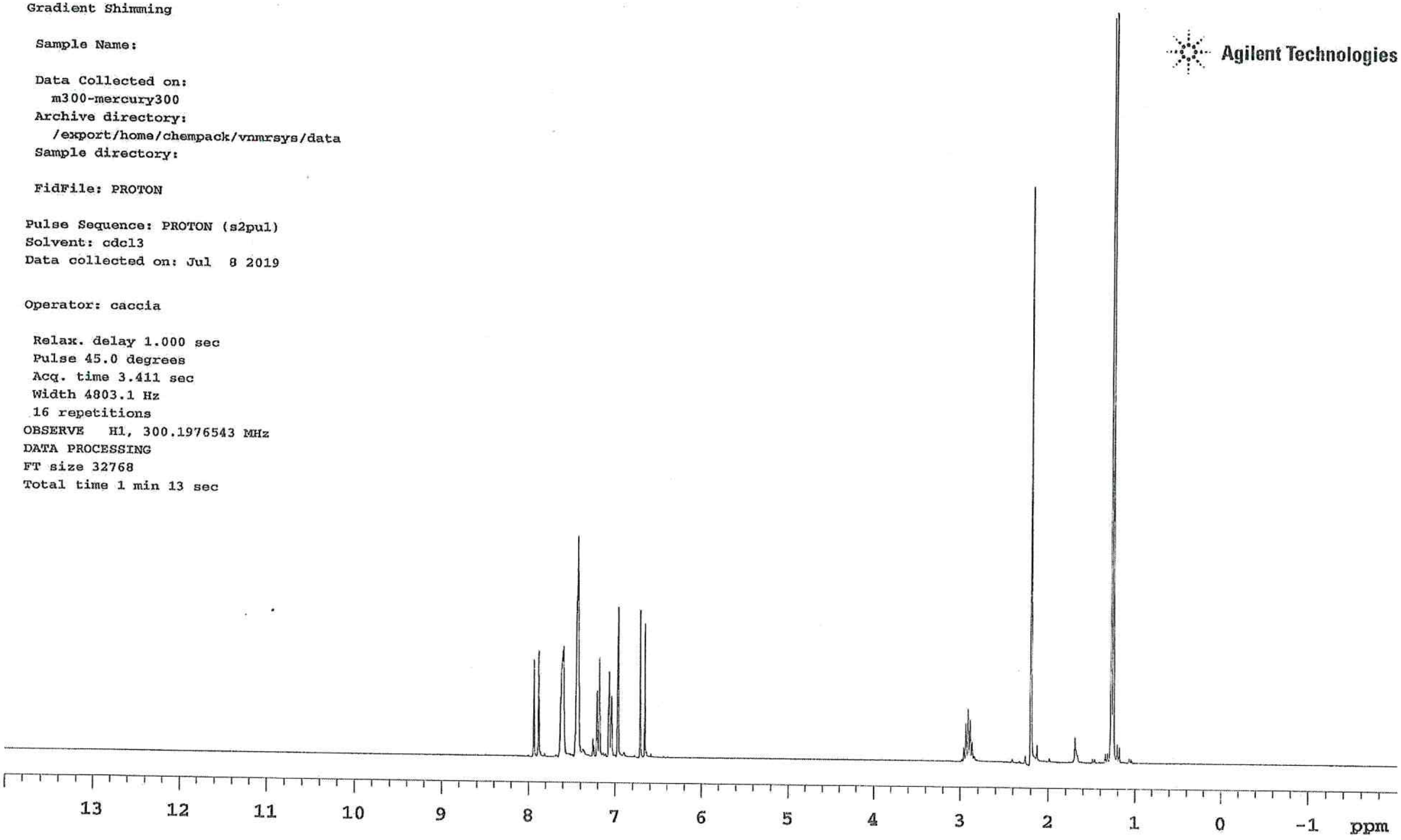

DH 1

Gradient Shimming

Sample Name:

Data Collected on:
m300-mercury300
Archive directory:
/export/home/chempack/vnmrsys/data
Sample directory:

FidFile: CARBON

Pulse Sequence: CARBON (s2pul)
Solvent: cdcl3
Data collected on: Jul 22 2019

Operator: caccia

Relax. delay 1.000 sec
Pulse 45.0 degrees
Acq. time 0.868 sec
Width 18867.9 Hz
2000 repetitions
OBSERVE C13, 75.4847602 MHz
DECOUPLE H1, 300.1991980 MHz
Power 38 dB
continuously on
WALTZ-16 modulated
DATA PROCESSING
Line broadening 0.5 Hz
FT size 32768
Total time 1 hr, 4 min

Agilent Technologies

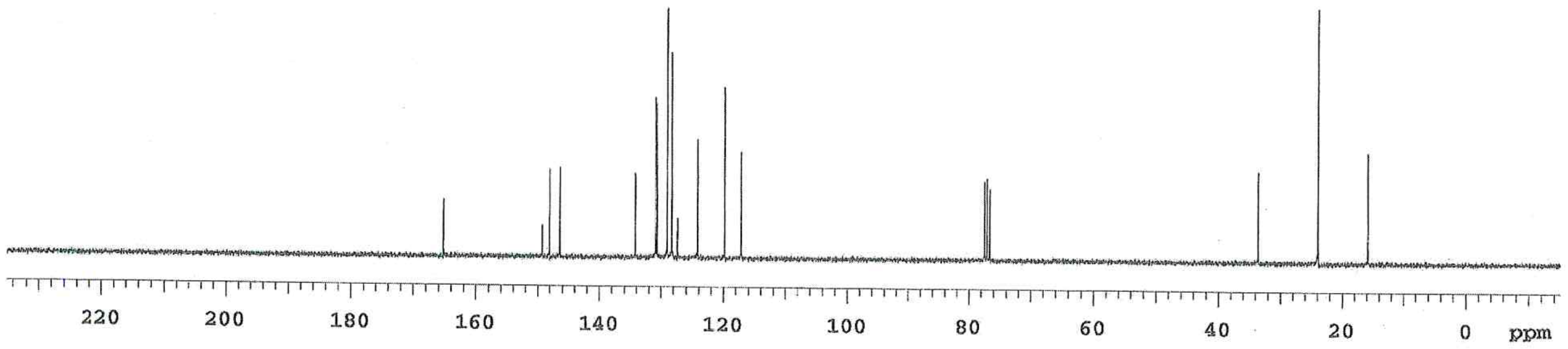

DM2

Gradient Shimming

Sample Name:
DM2
Data Collected on:
m300-mercury300
Archive directory:
/export/home/chempack/vnmrsys/data
Sample directory:

FidFile: PROTON

Pulse Sequence: PROTON (s2pul)
Solvent: dmso
Data collected on: Jun 11 2021

Operator: caccia

Relax. delay 1.000 sec
Pulse 45.0 degrees
Acq. time 1.706 sec
Width 4803.1 Hz
64 repetitions
OBSERVE H1, 300.1990802 MHz
DATA PROCESSING
FT size 16384
Total time 2 min 58 sec

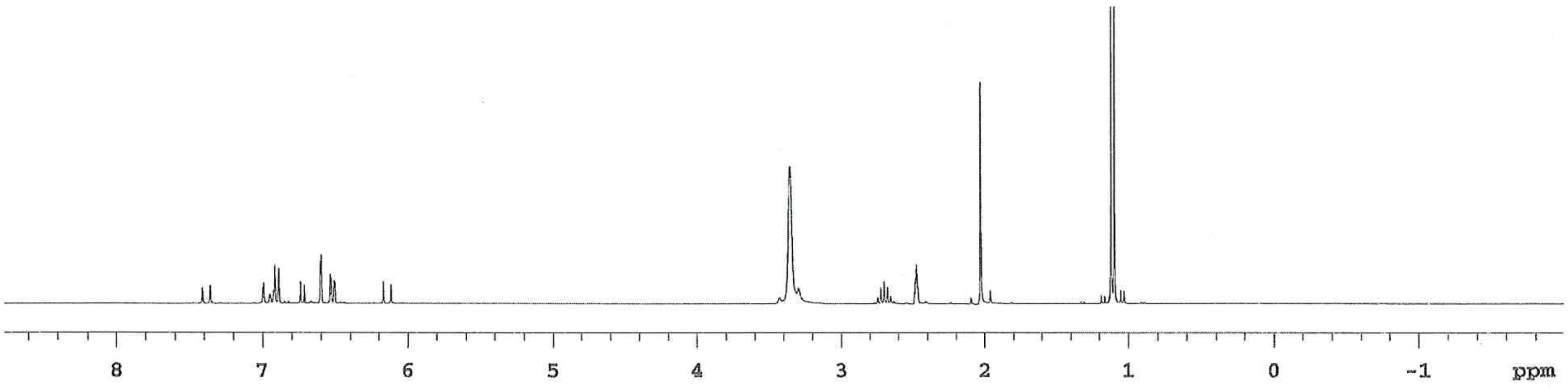

DM2

Gradient Shimming

Sample Name:
DM2
Data Collected on:
m300-mercury300
Archive directory:
/export/home/chempack/vnmrsys/data
Sample directory:

FidFile: CARBON

Pulse Sequence: CARBON (s2pul)
Solvent: dmso
Data collected on: Jun 9 2021

Operator: caccia

Relax. delay 1.000 sec
Pulse 45.0 degrees
Acq. time 0.868 sec
Width 18867.9 Hz
30016 repetitions
OBSERVE C13, 75.4851188 MHz
DECOUPLE H1, 300.2006240 MHz
Power 38 dB
continuously on
WALTZ-16 modulated
DATA PROCESSING
Line broadening 0.5 Hz
FT size 32768
Total time 26 hr, 50 min

Agilent Technologies

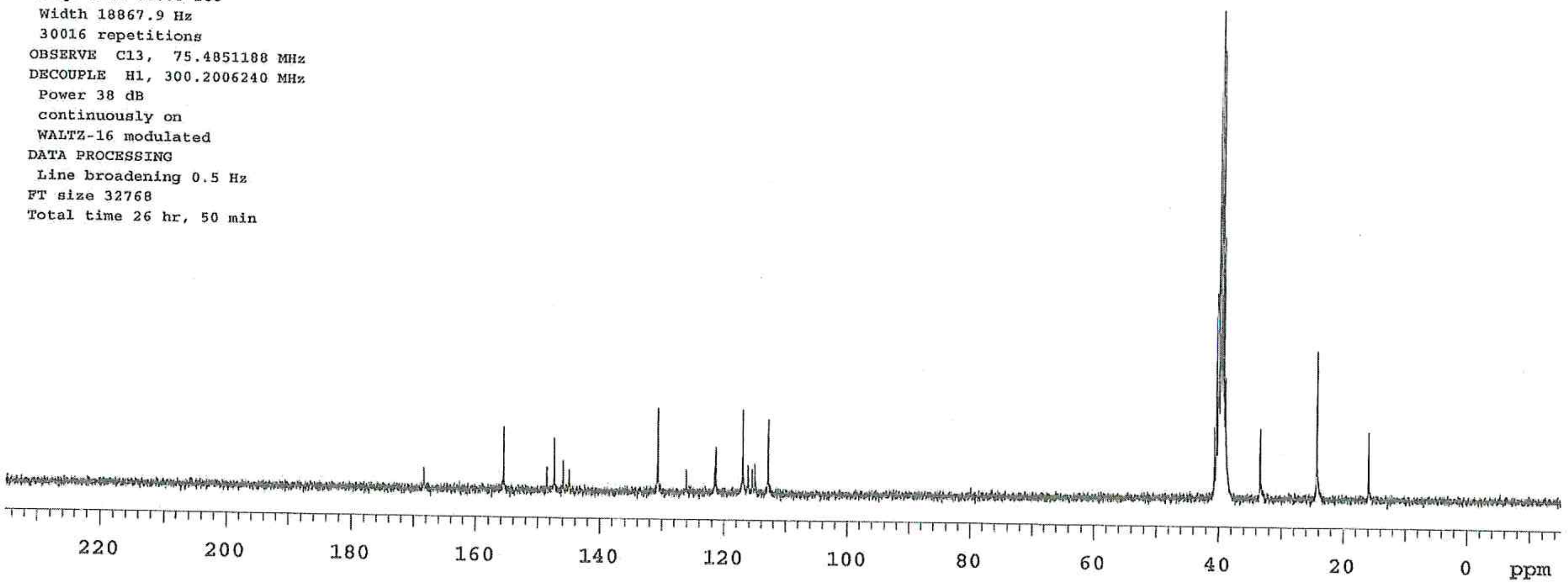

DH3

Gradient Shimming

Sample Name:

Data Collected on:
m300-mercury300
Archive directory:
/export/home/chempack/vnmrsys/data
Sample directory:

FidFile: PROTON

Pulse Sequence: PROTON (s2pul)
Solvent: cdcl3
Data collected on: Nov 26 2019

Operator: caccia

Relax. delay 1.000 sec
Pulse 45.0 degrees
Acq. time 1.706 sec
Width 4803.1 Hz
32 repetitions
OBSERVE H1, 300.1976543 MHz
DATA PROCESSING
FT size 16384
Total time 1 min 29 sec

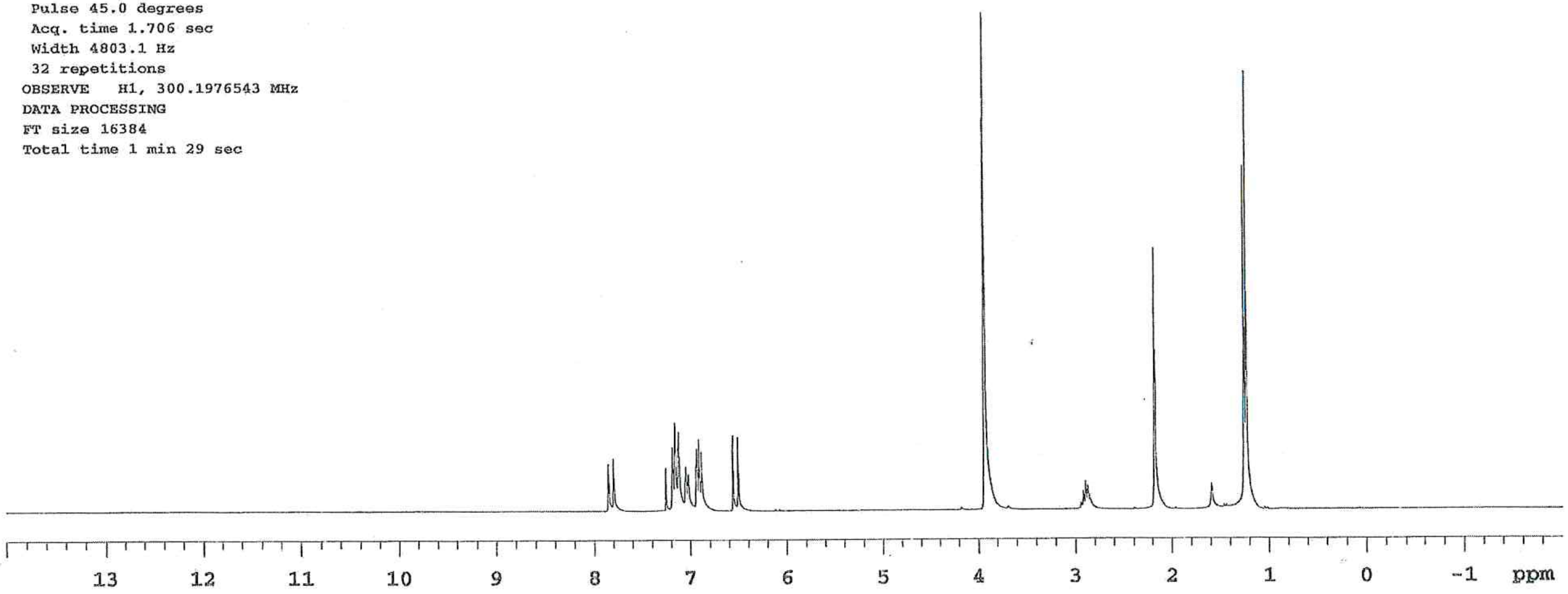

DM3

Gradient Shimming

Sample Name:

Data Collected on:
m300-mercury300
Archive directory:
/export/home/chempack/vnmrsys/data
Sample directory:

FidFile: CARBON

Pulse Sequence: CARBON (s2pul)
Solvent: dmso
Data collected on: Jul 10 2019

Temp. 18.4 C / 291.6 K
Operator: caccia

Relax. delay 1.000 sec
Pulse 45.0 degrees
Acq. time 0.868 sec
Width 18867.9 Hz
27456 repetitions
OBSERVE C13, 75.4851188 MHz
DECOUPLE H1, 300.2006240 MHz
Power 38 dB
continuously on
WALTZ-16 modulated
DATA PROCESSING
Line broadening 0.5 Hz
FT size 32768
Total time 53 hr, 41 min

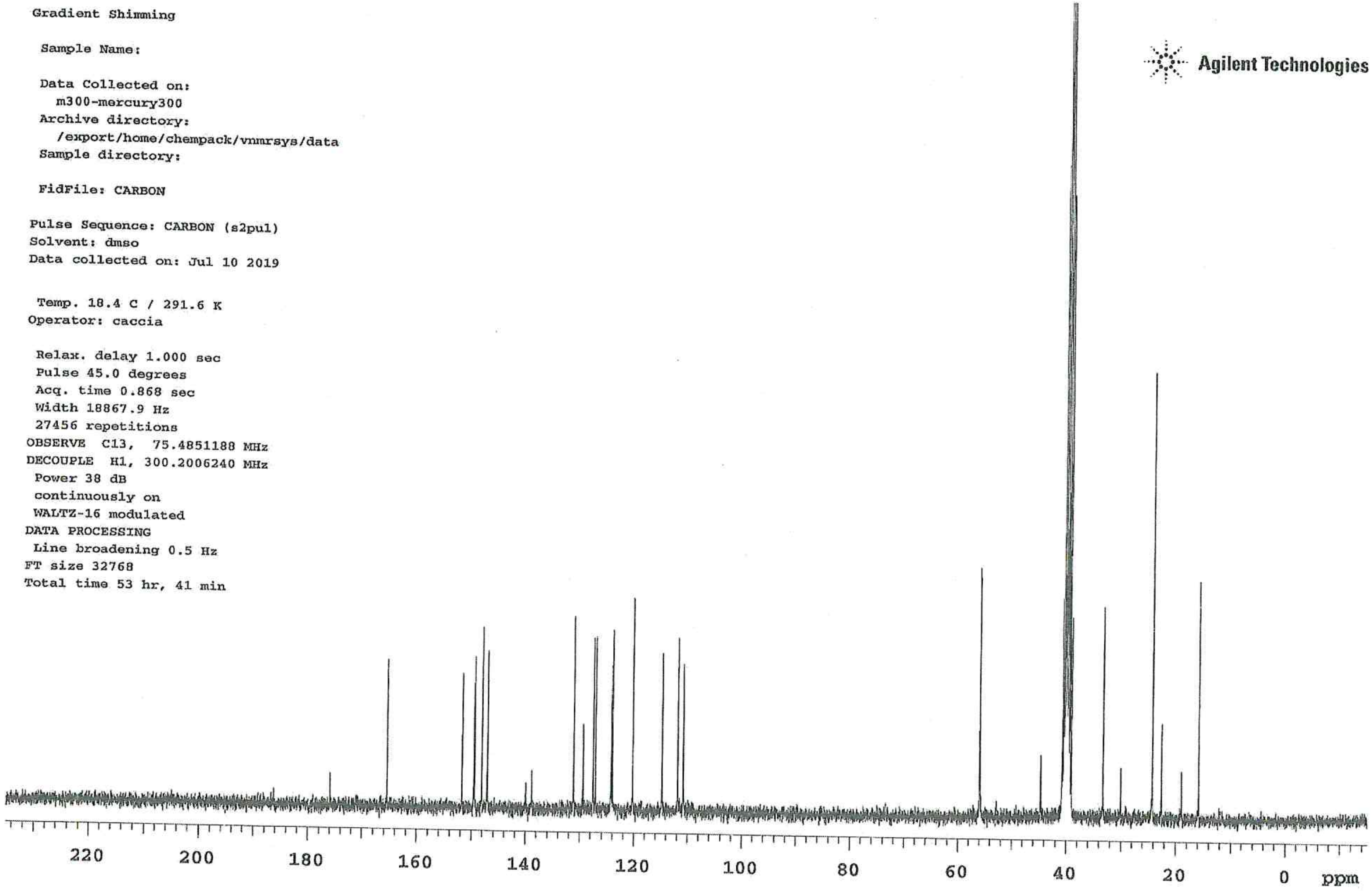

DH4

Gradient Shimming

Sample Name:

Data Collected on:
m300-mercury300
Archive directory:
/export/home/chempack/vnmrsys/data
Sample directory:

FidFile: PROTON

Pulse Sequence: PROTON (s2pul)
Solvent: cdcl3
Data collected on: Jan 15 2020

Operator: caccia

Relax. delay 1.000 sec
Pulse 45.0 degrees
Acq. time 1.706 sec
Width 4803.1 Hz
32 repetitions
OBSERVE H1, 300.1976543 MHz
DATA PROCESSING
FT size 16384
Total time 1 min 29 sec

Agilent Technologies

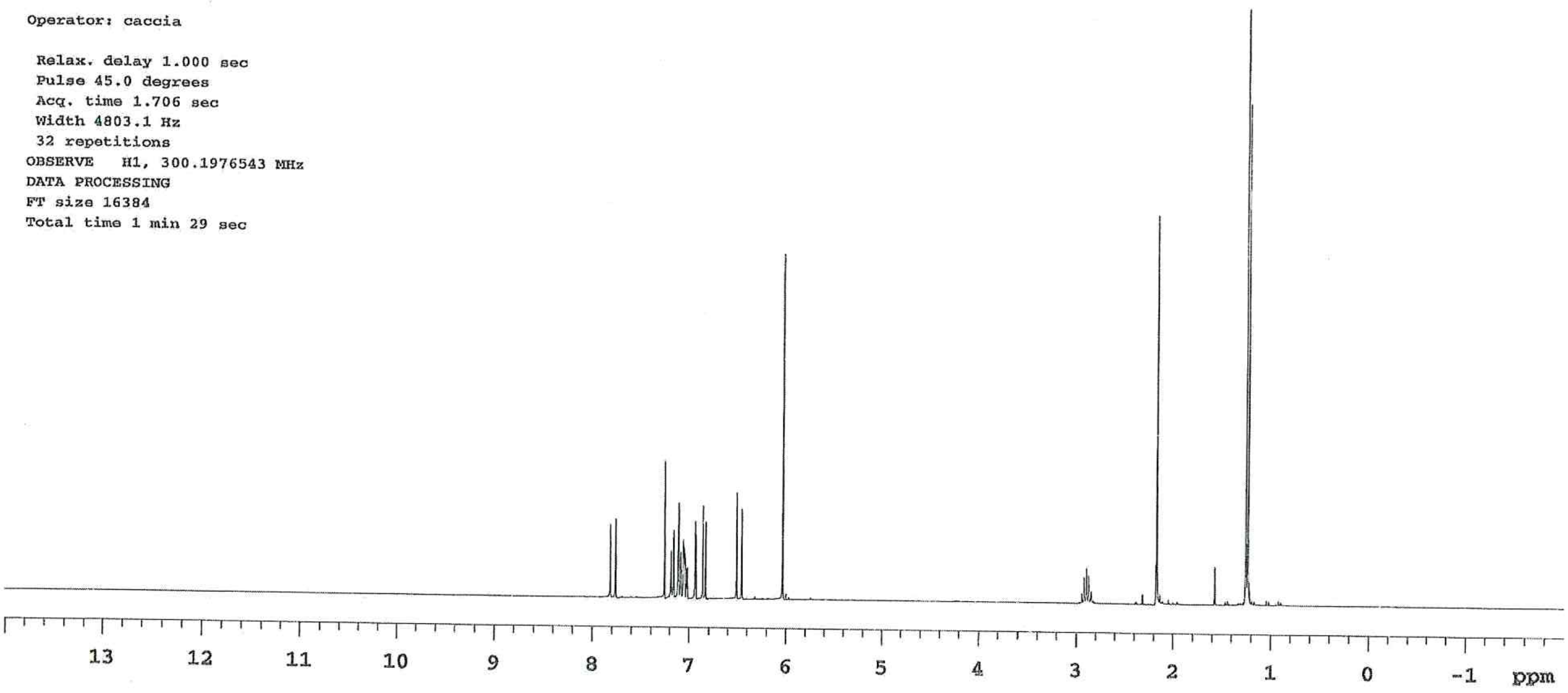

DH4

Gradient Shimming

Sample Name:

Data Collected on:
m300-mercury300
Archive directory:
/export/home/chempack/vnmrsys/data
Sample directory:

FidFile: CARBON

Pulse Sequence: CARBON (s2pul)
Solvent: cdcl3
Data collected on: Jan 15 2020

Operator: caccia

Relax. delay 1.000 sec
Pulse 45.0 degrees
Acq. time 0.868 sec
Width 18867.9 Hz
10000 repetitions
OBSERVE C13, 75.4847602 MHz
DECOUPLE H1, 300.1991980 MHz
Power 38 dB
continuously on
WALTZ-16 modulated
DATA PROCESSING
Line broadening 0.5 Hz
FT size 32768
Total time 5 hr, 22 min

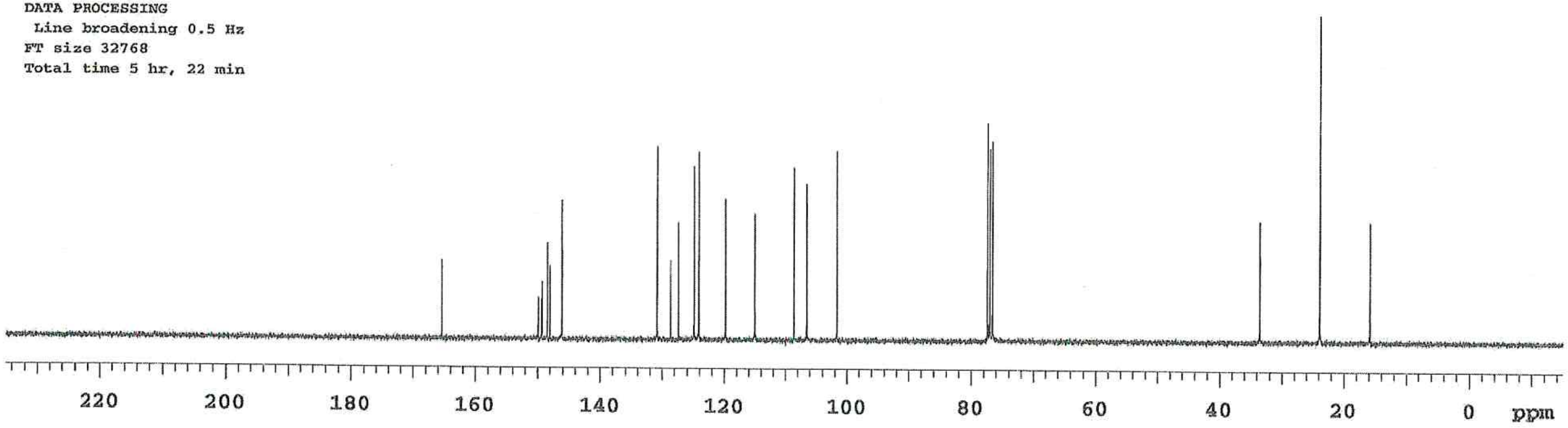

DM5

Gradient Shimming

Sample Name:
DM5
Data Collected on:
m300-mercury300
Archive directory:
/export/home/chempack/vnmrsys/data
Sample directory:

FidFile: PROTON

Pulse Sequence: PROTON (s2pul)
Solvent: dmso
Data collected on: Jun 10 2021

Operator: caccia

Relax. delay 1.000 sec
Pulse 45.0 degrees
Acq. time 1.706 sec
Width 4803.1 Hz
64 repetitions
OBSERVE H1, 300.1990802 MHz
DATA PROCESSING
FT size 16384
Total time 2 min 58 sec

Agilent Technologies

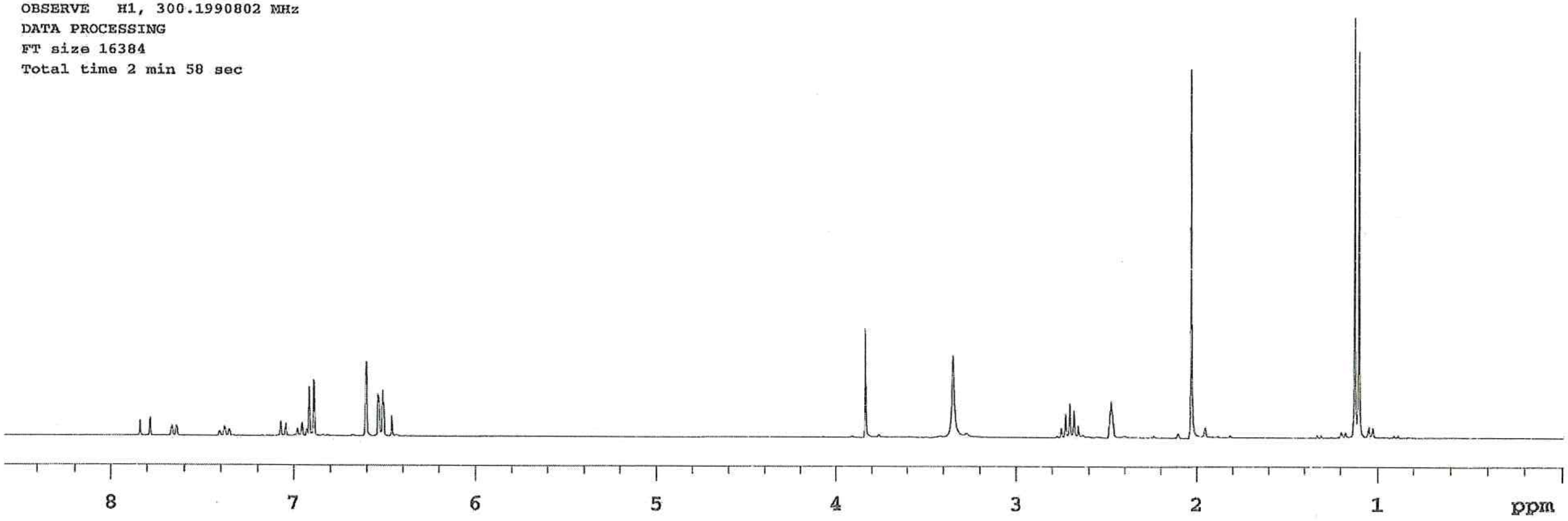

DM5

Gradient Shimming

Sample Name:
DM5
Data Collected on:
m300-mercury300
Archive directory:
/export/home/chempack/vnmrsys/data
Sample directory:

FidFile: CARBON

Pulse Sequence: CARBON (s2pul)
Solvent: dmso
Data collected on: Jun 10 2021

Operator: caccia

Relax. delay 1.000 sec
Pulse 45.0 degrees
Acq. time 0.868 sec
Width 18867.9 Hz
1792 repetitions
OBSERVE C13, 75.4851188 MHz
DECOUPLE H1, 300.2006240 MHz
Power 38 dB
continuously on
WALTZ-16 modulated
DATA PROCESSING
Line broadening 0.5 Hz
FT size 32768
Total time 1 hr, 4 min

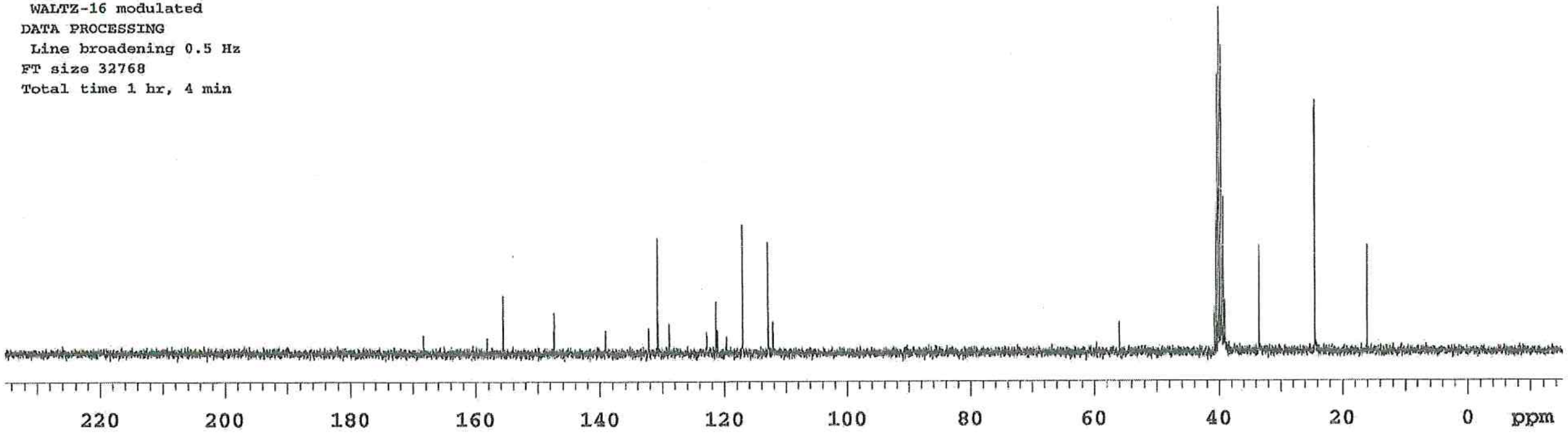

S18

DM6

Gradient Shimming

Sample Name:

Data Collected on:
m300-mercury300
Archive directory:
/export/home/chempack/vnmrsys/data
Sample directory:

FidFile: PROTON

Pulse Sequence: PROTON (s2pul)
Solvent: cdcl3
Data collected on: Dec 12 2019

Operator: caccia

Relax. delay 1.000 sec
Pulse 45.0 degrees
Acq. time 1.706 sec
Width 4803.1 Hz
32 repetitions
OBSERVE H1, 300.1976543 MHz
DATA PROCESSING
FT size 16384
Total time 1 min 29 sec

Agilent Technologies

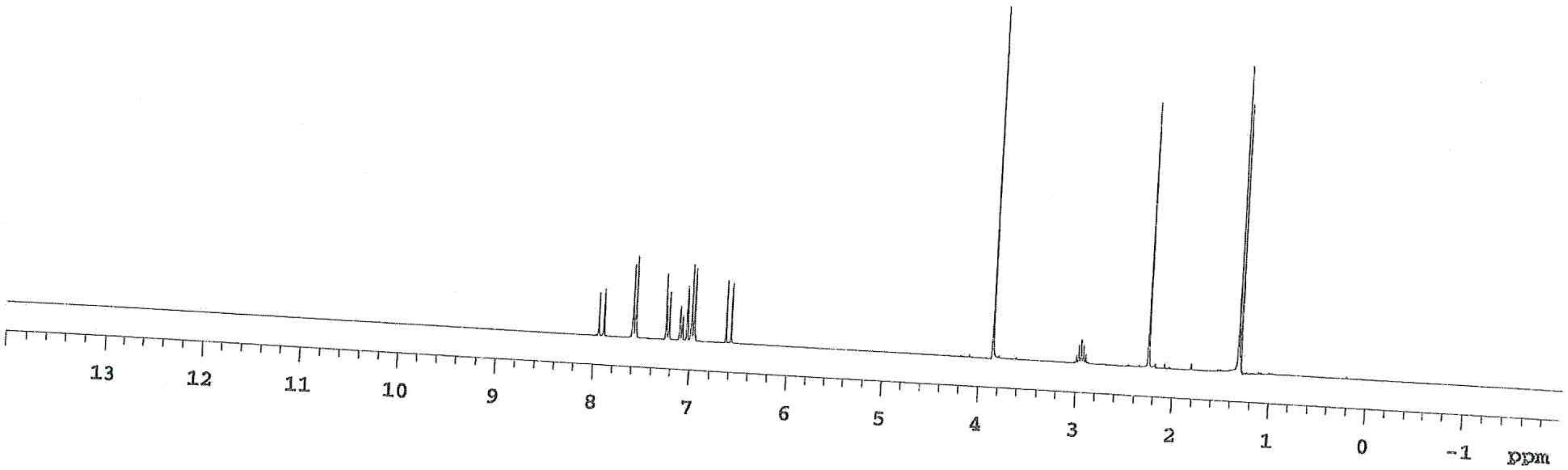

DH6

Gradient Shimming

Sample Name:

Data Collected on:
m300-mercury300
Archive directory:
/export/home/chempack/vnmrsys/data
Sample directory:

FidFile: CARBON

Pulse Sequence: CARBON (s2pul)
Solvent: dmso
Data collected on: Jul 15 2019

Operator: caccia

Relax. delay 1.000 sec
Pulse 45.0 degrees
Acq. time 0.868 sec
Width 18867.9 Hz
2000 repetitions
OBSERVE C13, 75.4851188 MHz
DECOUPLE H1, 300.2006240 MHz
Power 38 dB
continuously on
WALTZ-16 modulated
DATA PROCESSING
Line broadening 0.5 Hz
FT size 32768
Total time 1 hr, 4 min

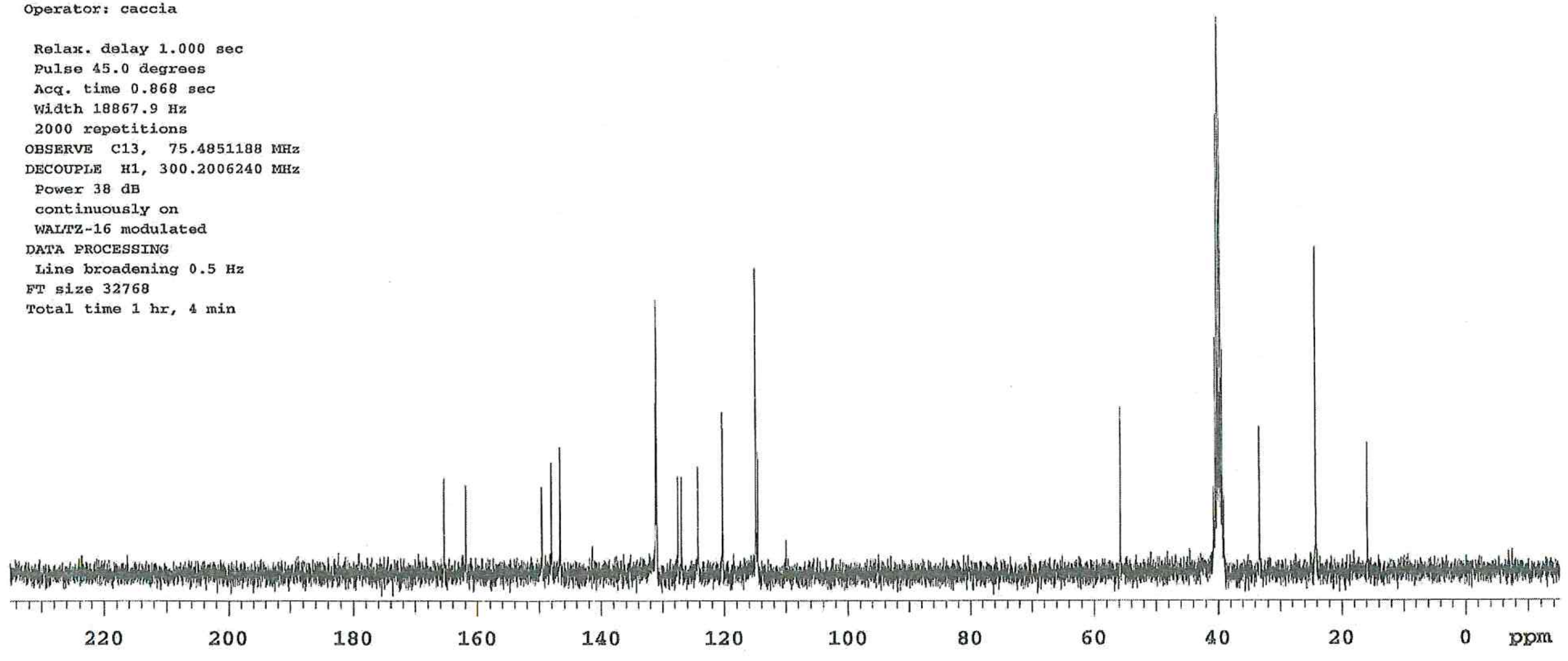

DM7

Gradient Shimming

Sample Name:
DM7
Data Collected on:
m300-mercury300
Archive directory:
/export/home/chempack/vnmrsys/data
Sample directory:

FidFile: PROTON

Pulse Sequence: PROTON (s2pul)
Solvent: dmso
Data collected on: Jun 10 2021

Operator: caccia

Relax. delay 1.000 sec
Pulse 45.0 degrees
Acq. time 1.706 sec
Width 4803.1 Hz
64 repetitions
OBSERVE H1, 300.1990802 MHz
DATA PROCESSING
FT size 16384
Total time 2 min 58 sec

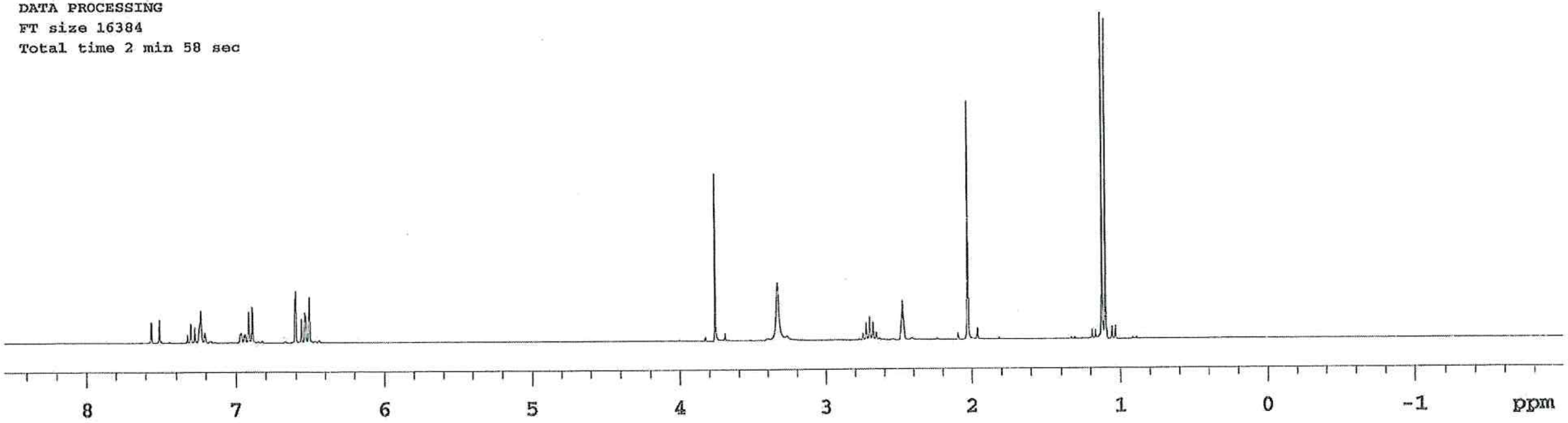

DM7

Gradient Shimming

Sample Name:
DM7
Data Collected on:
m300-mercury300
Archive directory:
/export/home/chempack/vnmrsys/data
Sample directory:

FidFile: CARBON

Pulse Sequence: CARBON (s2pul)
Solvent: dmso
Data collected on: Jun 10 2021

Operator: caccia

Relax. delay 1.000 sec
Pulse 45.0 degrees
Acq. time 0.868 sec
Width 18867.9 Hz
1216 repetitions
OBSERVE C13, 75.4851188 MHz
DECOUPLE H1, 300.2006240 MHz
Power 38 dB
continuously on
WALTZ-16 modulated
DATA PROCESSING
Line broadening 0.5 Hz
FT size 32768
Total time 1 hr, 4 min

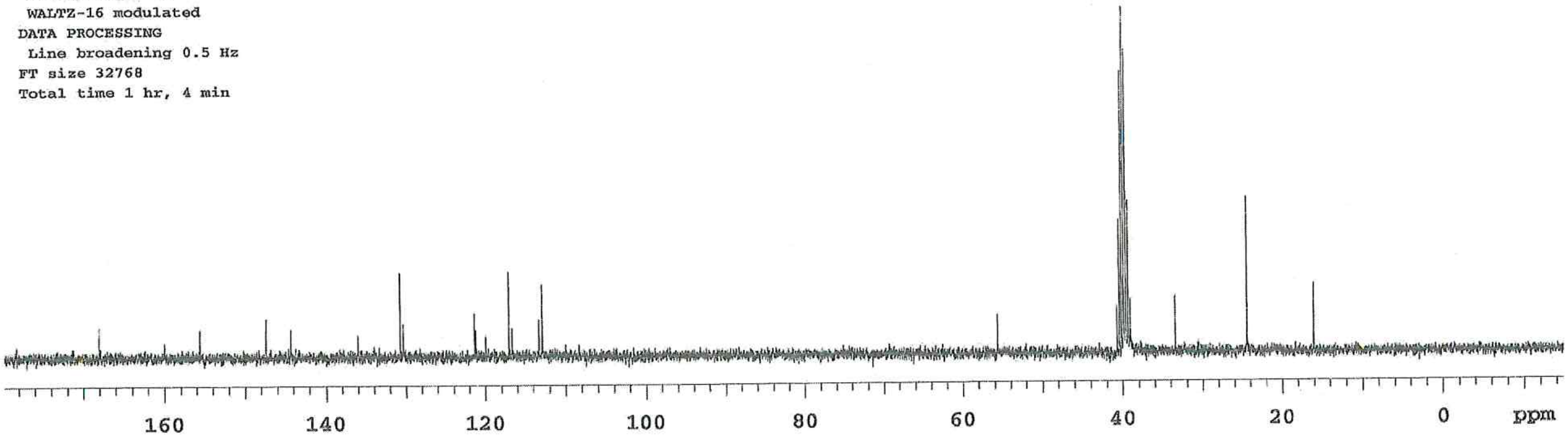

DM8

Gradient Shimming

Sample Name:

Data Collected on:
m300-mercury300
Archive directory:
/export/home/chempack/vnmrsys/data
Sample directory:

FidFile: PROTON

Pulse Sequence: PROTON (s2pul)
Solvent: cdcl3
Data collected on: Nov 29 2019

Operator: caccia

Relax. delay 1.000 sec
Pulse 45.0 degrees
Acq. time 1.706 sec
Width 4803.1 Hz
32 repetitions
OBSERVE H1, 300.1976543 MHz
DATA PROCESSING
FT size 16384
Total time 1 min 29 sec

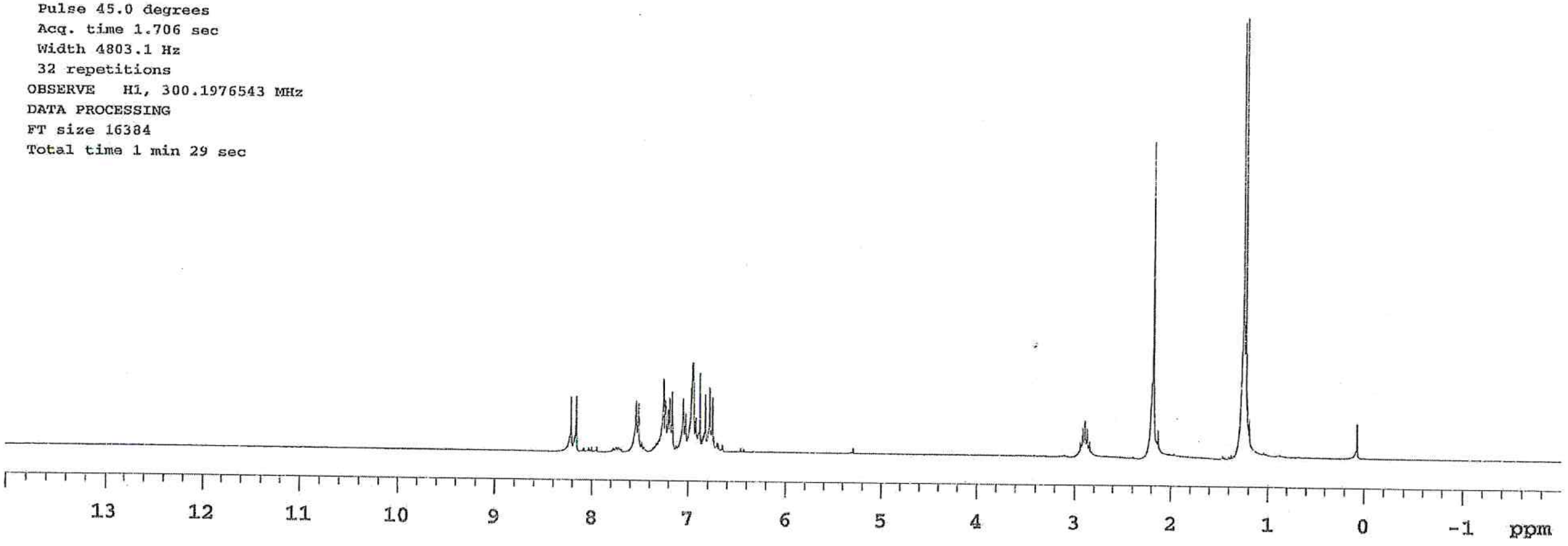

DM8

Gradient Shimming

Sample Name:

Data Collected on:
m300-mercury300
Archive directory:
/export/home/chempack/vnmrsys/data
Sample directory:

FidFile: CARBON

Pulse Sequence: CARBON (s2pul)
Solvent: cdcl3
Data collected on: Dec 9 2019

Operator: caccia

Relax. delay 1.000 sec
Pulse 45.0 degrees
Acq. time 0.868 sec
Width 18867.9 Hz
24000 repetitions
OBSERVE C13, 75.4847602 MHz
DECOUPLE H1, 300.1991980 MHz
Power 38 dB
continuously on
WALTZ-16 modulated
DATA PROCESSING
Line broadening 0.5 Hz
FT size 32768
Total time 12 hr, 53 min

Agilent Technologies

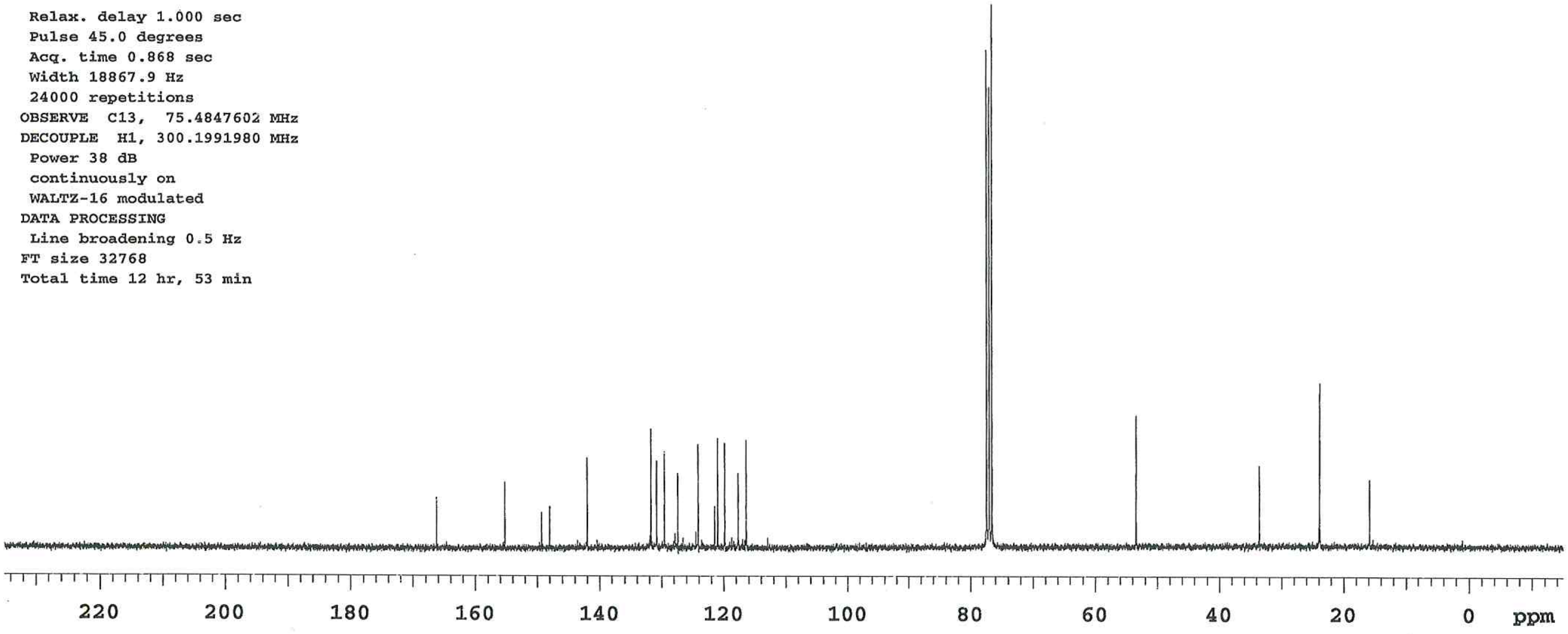

DH9

Gradient Shimming

Sample Name:

Data Collected on:
m300-mercury300
Archive directory:
/export/home/chempack/vnmrsys/data
Sample directory:

FidFile: PROTON

Pulse Sequence: PROTON (s2pul)
Solvent: cdcl3
Data collected on: Jan 28 2020

Operator: caccia

Relax. delay 1.000 sec
Pulse 45.0 degrees
Acq. time 1.706 sec
Width 4803.1 Hz
32 repetitions
OBSERVE H1, 300.1976543 MHz
DATA PROCESSING
FT size 16384
Total time 1 min 29 sec

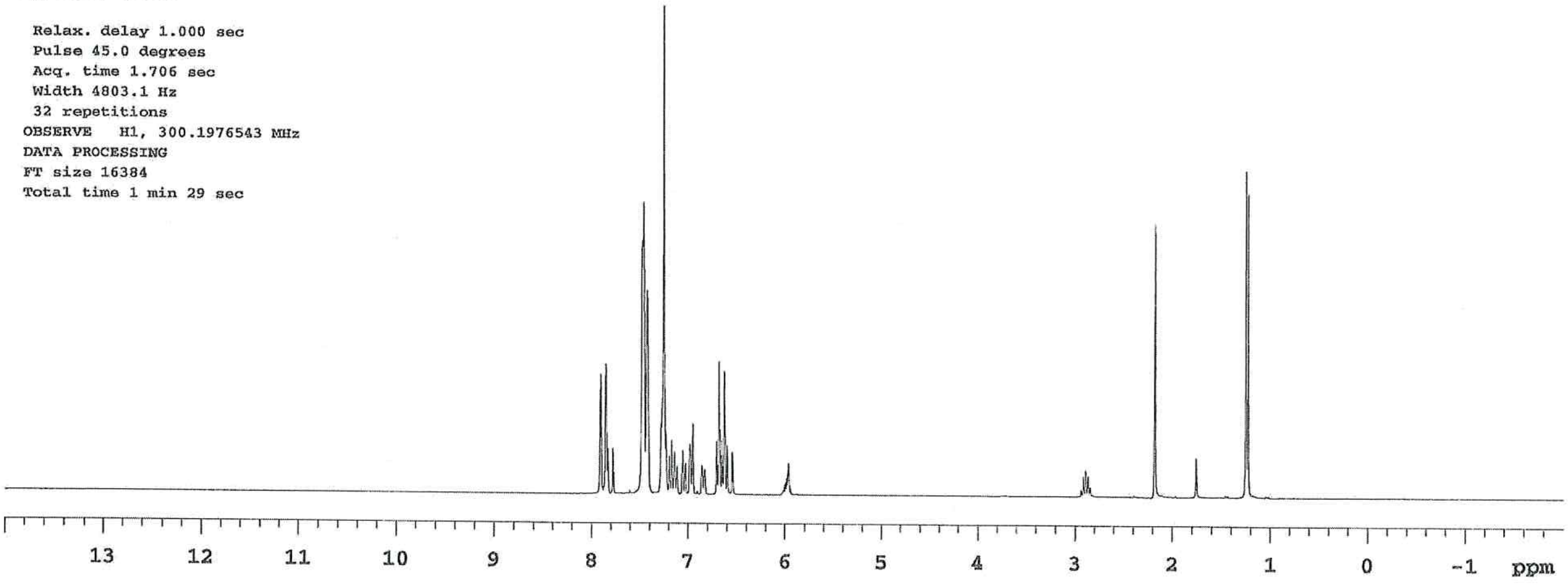

DH9

Gradient Shimming

Sample Name:

Data Collected on:
m300-mercury300
Archive directory:
/export/home/chempack/vnmrsys/data
Sample directory:

FidFile: CARBON

Pulse Sequence: CARBON (s2pul)
Solvent: cdcl3
Data collected on: Jan 28 2020

Operator: caccia

Relax. delay 1.000 sec
Pulse 45.0 degrees
Acq. time 0.868 sec
Width 18867.9 Hz
2000 repetitions
OBSERVE C13, 75.4847602 MHz
DECOUPLE H1, 300.1991980 MHz
Power 38 dB
continuously on
WALTZ-16 modulated
DATA PROCESSING
Line broadening 0.5 Hz
FT size 32768
Total time 1 hr, 4 min

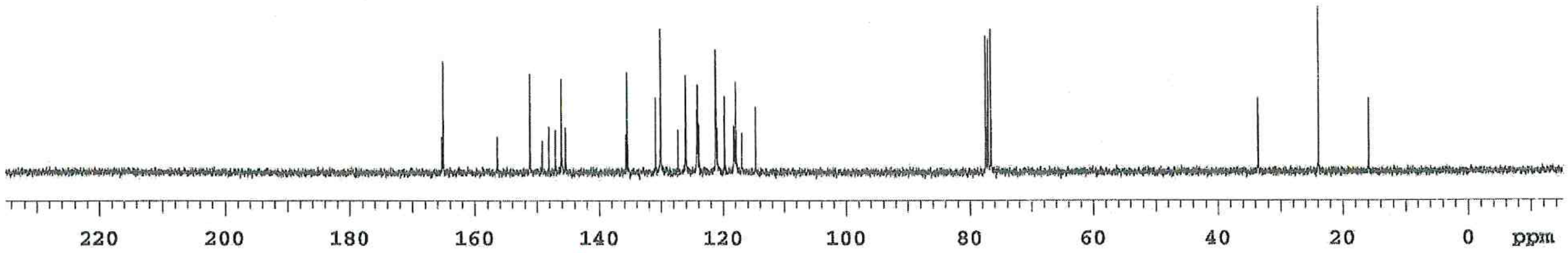

Gradient Shimming

Sample Name:

Data Collected on:
m300-mercury300
Archive directory:
/export/home/chempack/vnmrsys/data
Sample directory:

FidFile: PROTON

Pulse Sequence: PROTON (s2pul)
Solvent: cdcl3
Data collected on: Jan 8 2020

Operator: caccia

Relax. delay 1.000 sec
Pulse 45.0 degrees
Acq. time 1.706 sec
Width 4803.1 Hz
32 repetitions
OBSERVE H1, 300.1976543 MHz
DATA PROCESSING
FT size 16384
Total time 1 min 29 sec

Agilent Technologies

DM10

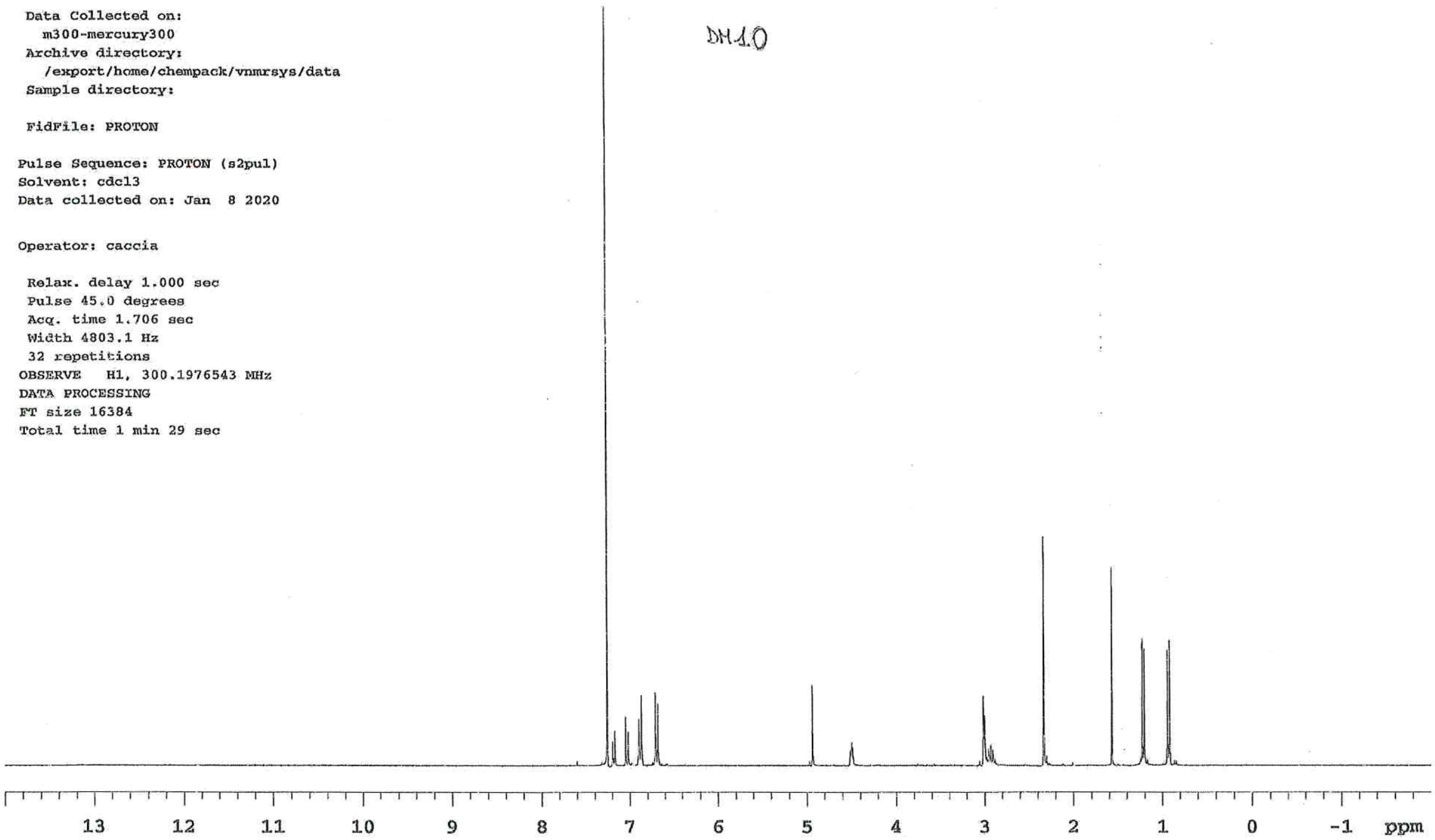

Gradient Shimming

Sample Name:

Data Collected on:
m300-mercury300
Archive directory:
/export/home/chempack/vnmrsys/data
Sample directory:

FidFile: CARBON

Pulse Sequence: CARBON (s2pul)
Solvent: cdcl3
Data collected on: Jan 8 2020

Operator: caccia

Relax. delay 1.000 sec
Pulse 45.0 degrees
Acq. time 0.868 sec
Width 18867.9 Hz
10000 repetitions
OBSERVE C13, 75.4847602 MHz
DECOUPLE H1, 300.1991980 MHz
Power 38 dB
continuously on
WALTZ-16 modulated
DATA PROCESSING
Line broadening 0.5 Hz
FT size 32768
Total time 5 hr, 22 min

Agilent Technologies

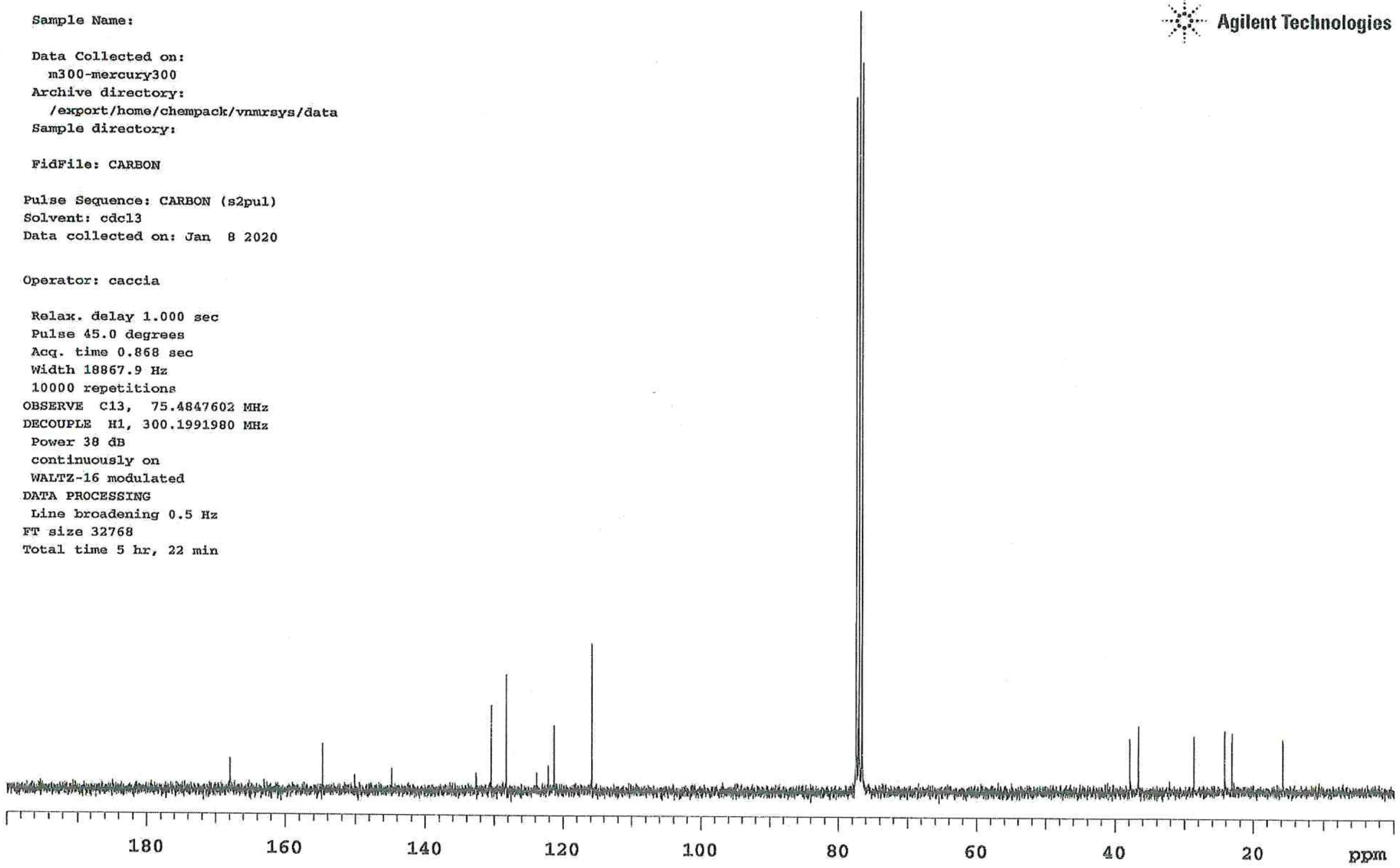

DH11

Gradient Shimming

Sample Name:

Data Collected on:
m300-mercury300
Archive directory:
/export/home/chempack/vnmrsys/data
Sample directory:

FidFile: PROTON

Pulse Sequence: PROTON (s2pul)
Solvent: cdcl3
Data collected on: Jan 15 2020

Operator: caccia

Relax. delay 1.000 sec
Pulse 45.0 degrees
Acq. time 1.706 sec
Width 4803.1 Hz
32 repetitions
OBSERVE H1, 300.1976543 MHz
DATA PROCESSING
FT size 16384
Total time 1 min 29 sec

Agilent Technologies

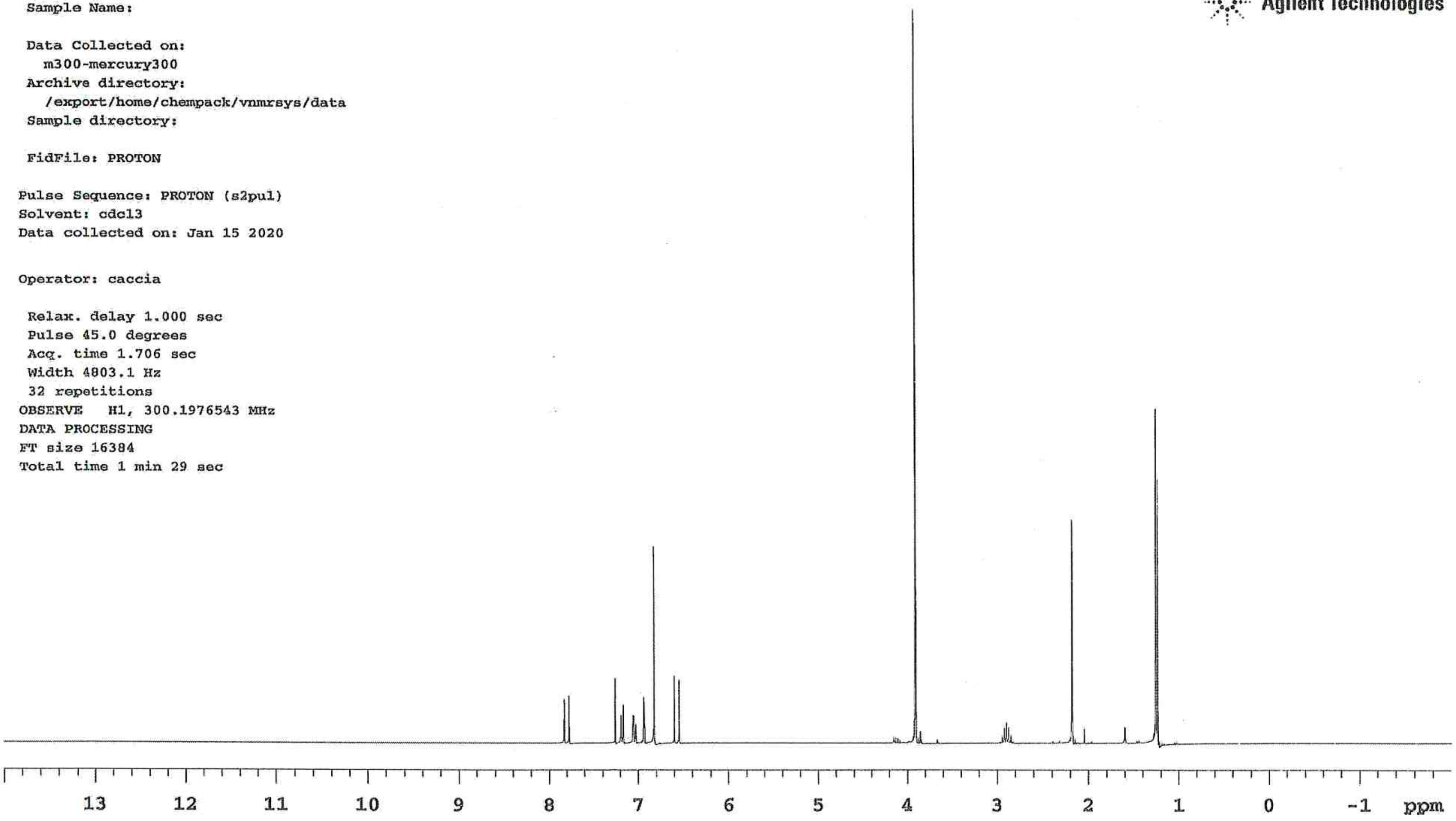

DH11

Gradient Shimming

Sample Name:

Data Collected on:
m300-mercury300
Archive directory:
/export/home/chempack/vnmrsys/data
Sample directory:

FidFile: CARBON

Pulse Sequence: CARBON (s2pul)
Solvent: cdcl3
Data collected on: Nov 6 2019

Operator: caccia

Relax. delay 1.000 sec
Pulse 45.0 degrees
Acq. time 0.868 sec
Width 18867.9 Hz
2000 repetitions
OBSERVE C13, 75.4847602 MHz
DECOUPLE H1, 300.1991980 MHz
Power 38 dB
continuously on
WALTZ-16 modulated
DATA PROCESSING
Line broadening 0.5 Hz
FT size 32768
Total time 1 hr, 4 min

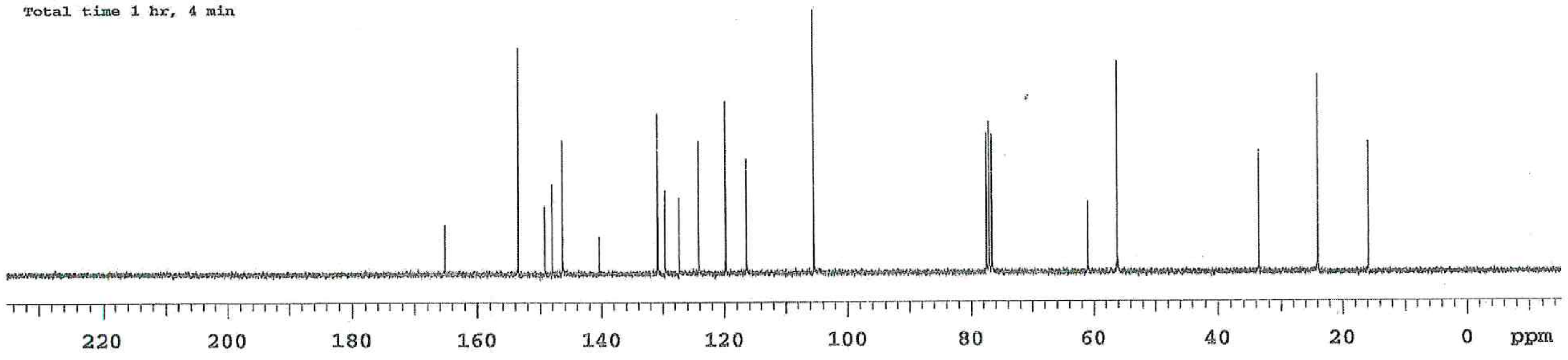

HR-MS SPECTRA OF DM1-11

High Resolution Mass Spectrometry Analysis
Each investigated compound was dissolved in ACN/H_2O 80/20 with 0.1% of formic acid at 10 µg/mL and injected into the mass spectrometer through a syringe pump at a flow rate of 5 µl/min. The mass spectrometer used was a Thermo Fischer Orbitrap Fusion^{TM} Tribrid^{TM} operating in MS scan in the m/z range of 80 to 500, equipped with the Orbitrap as detector type at 240,000 of mass resolution (FWHM). Except for the **DM2** all compounds were acquired in positive ion mode.

DM1 (280.15)

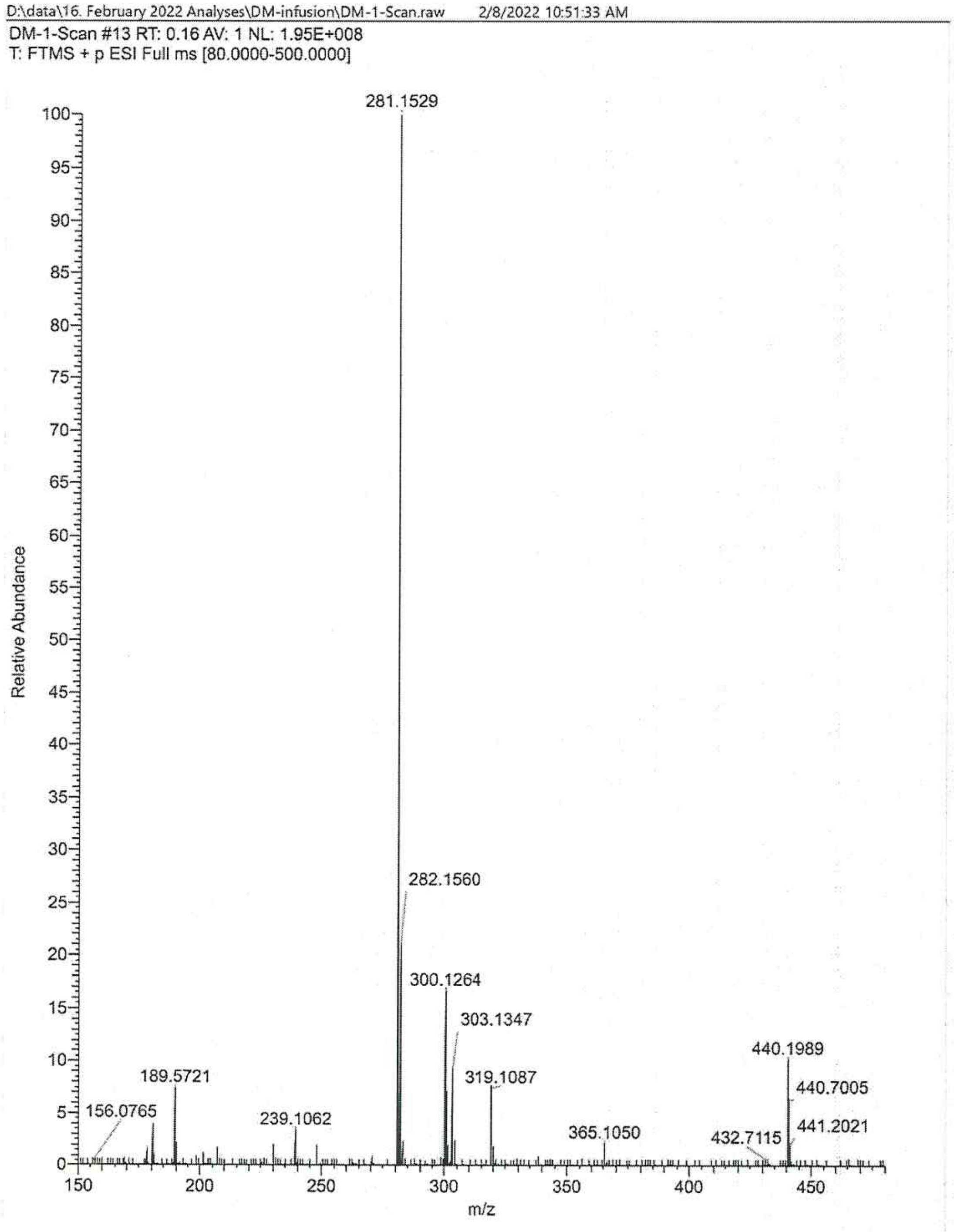

DM2 (312.16)

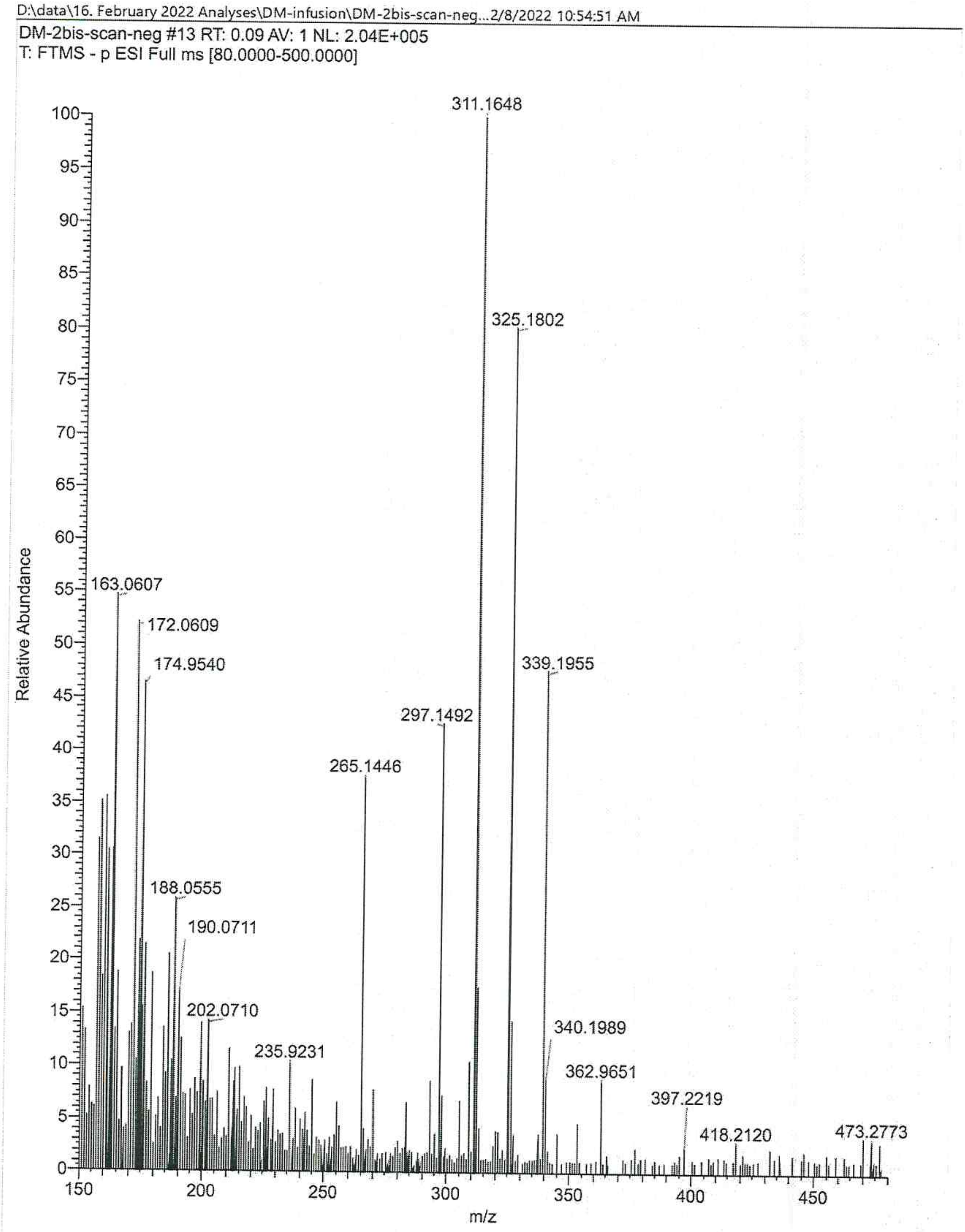
D:\data\16. February 2022 Analyses\DM-infusion\DM-2bis-scan-neg...2/8/2022 10:54:51 AM
DM-2bis-scan-neg #13 RT: 0.09 AV: 1 NL: 2.04E+005
T: FTMS - p ESI Full ms [80.0000-500.0000]
311.1648
325.1802
163.0607
172.0609
174.9540
339.1955
297.1492
265.1446
188.0555
190.0711
202.0710
340.1989
235.9231
362.9651
397.2219
418.2120
473.2773
Relative Abundance
m/z

DM3 (340.17)

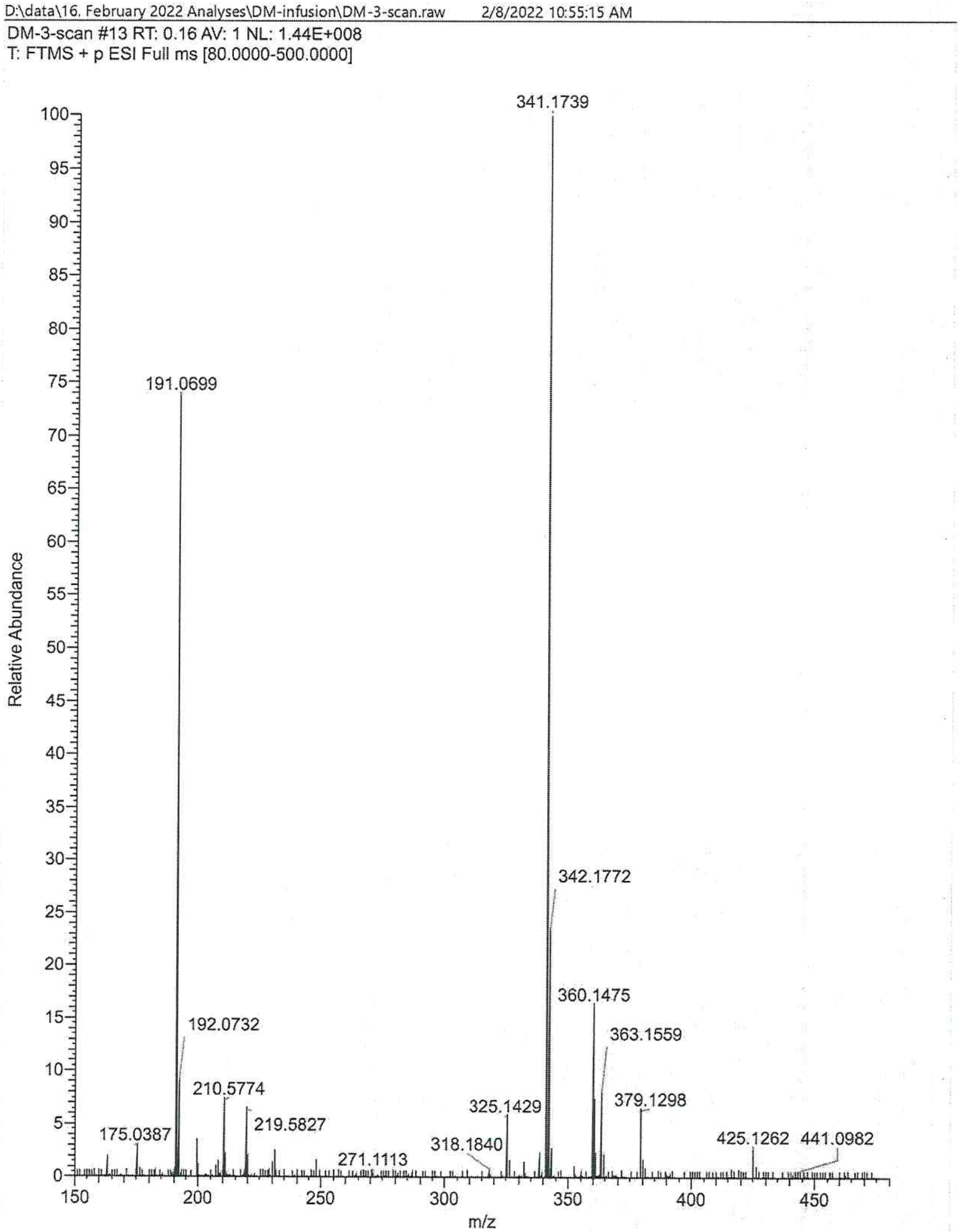
D:\data\16. February 2022 Analyses\DM-infusion\DM-3-scan.raw 2/8/2022 10:55:15 AM
DM-3-scan #13 RT: 0.16 AV: 1 NL: 1.44E+008
T: FTMS + p ESI Full ms [80.0000-500.0000]
341.1739
191.0699
342.1772
360.1475
363.1559
192.0732
210.5774
219.5827
379.1298
325.1429
175.0387
318.1840
271.1113
425.1262
441.0982
Relative Abundance
m/z

DM4 (324.14)

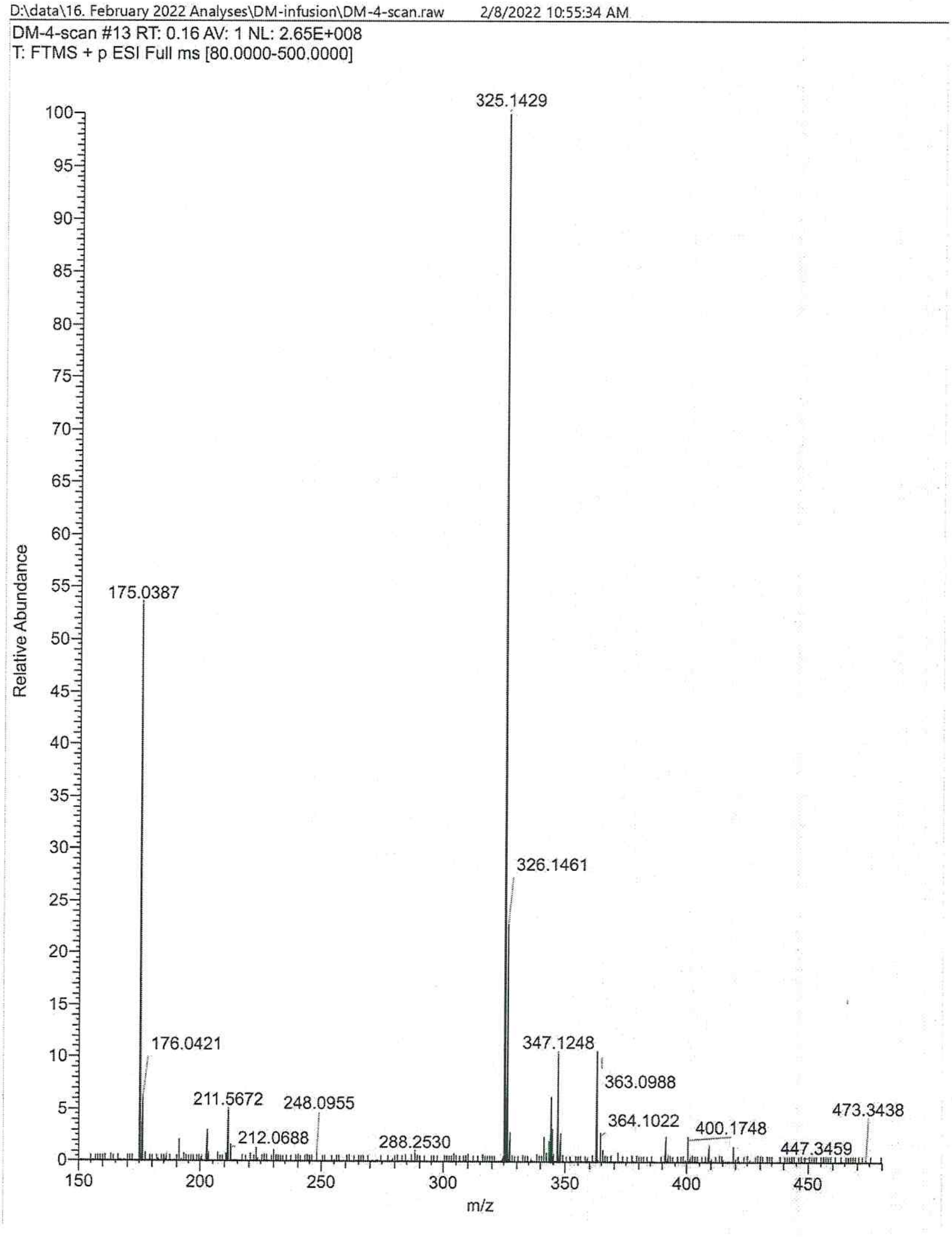

D:\data\16. February 2022 Analyses\DM-infusion\DM-4-scan.raw 2/8/2022 10:55:34 AM
DM-4-scan #13 RT: 0.16 AV: 1 NL: 2.65E+008
T: FTMS + p ESI Full ms [80.0000-500.0000]
325.1429
175.0387
326.1461
176.0421
347.1248
363.0988
211.5672
248.0955
473.3438
364.1022
400.1748
212.0688
288.2530
447.3459
Relative Abundance
m/z

DM5 (310.16)

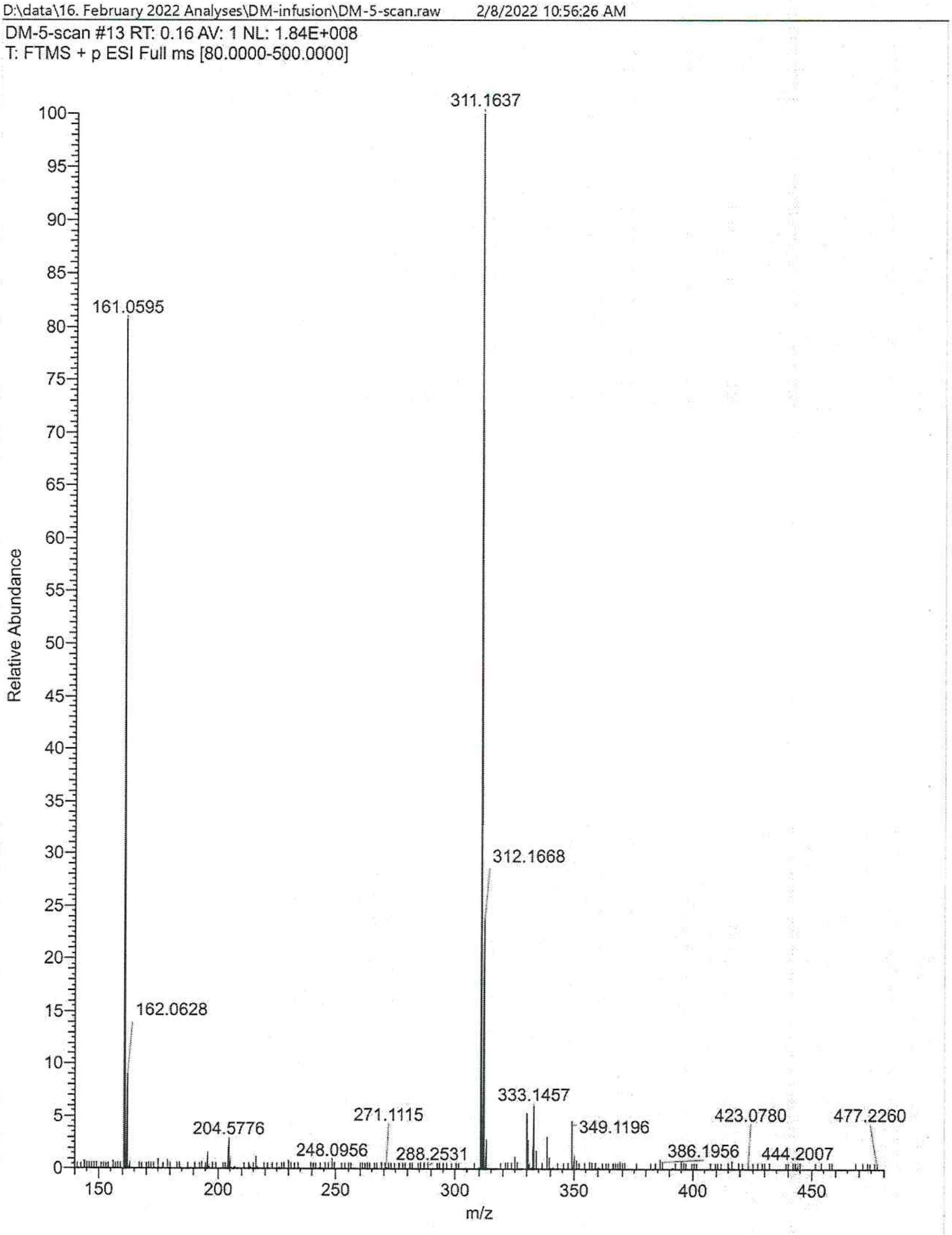
D:\data\16. February 2022 Analyses\DM-infusion\DM-5-scan.raw 2/8/2022 10:56:26 AM
DM-5-scan #13 RT: 0.16 AV: 1 NL: 1.84E+008
T: FTMS + p ESI Full ms [80.0000-500.0000]
311.1637
161.0595
312.1668
162.0628
333.1457
349.1196
204.5776
271.1115
248.0956
288.2531
386.1956
423.0780
444.2007
477.2260
Relative Abundance
m/z

DM6 (310.16)

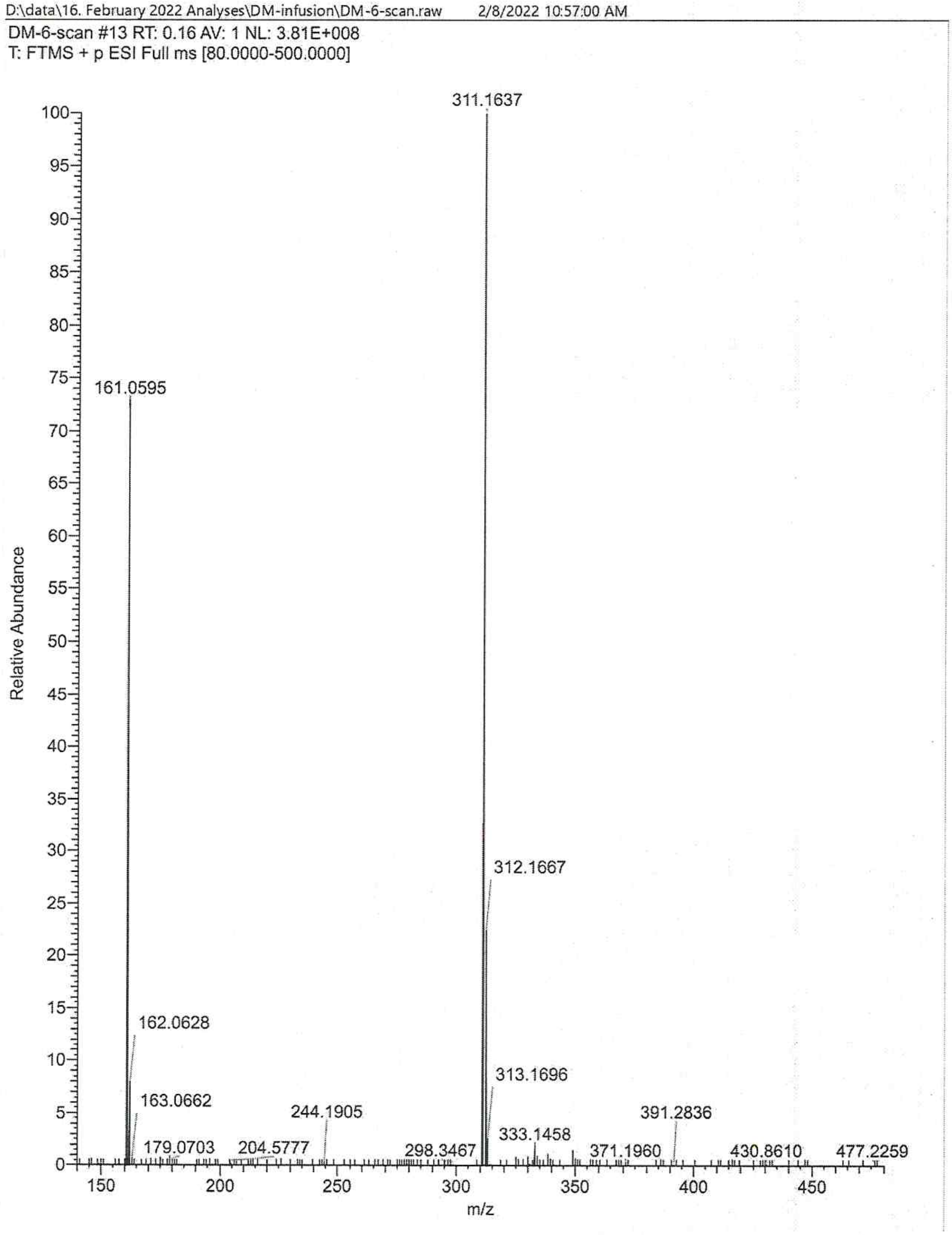

D:\data\16. February 2022 Analyses\DM-infusion\DM-6-scan.raw 2/8/2022 10:57:00 AM
DM-6-scan #13 RT: 0.16 AV: 1 NL: 3.81E+008
T: FTMS + p ESI Full ms [80.0000-500.0000]
Relative Abundance
m/z
311.1637
161.0595
312.1667
162.0628
313.1696
163.0662
244.1905
391.2836
333.1458
179.0703
204.5777
298.3467
371.1960
430.8610
477.2259

DM7 (310.16)

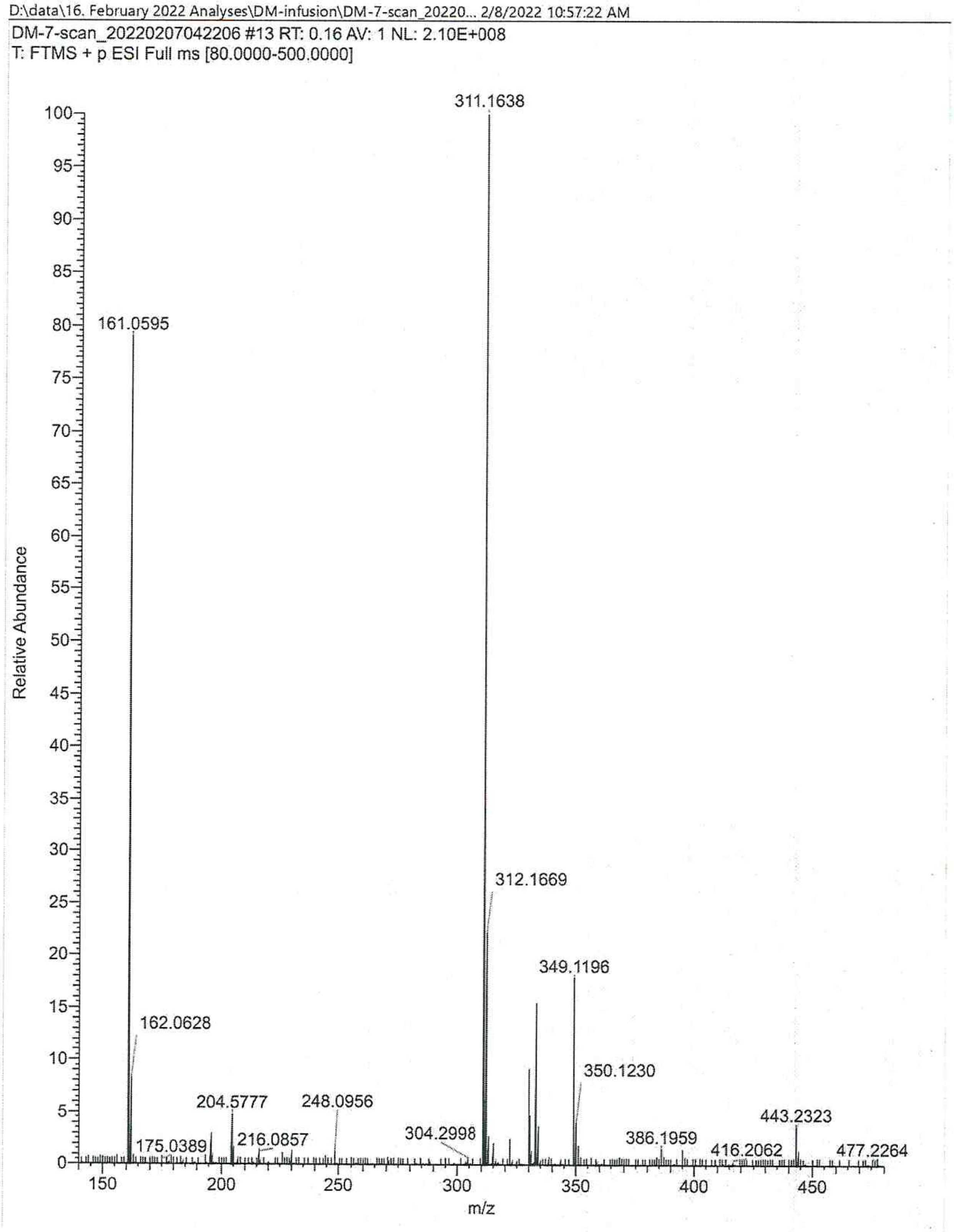

D:\data\16. February 2022 Analyses\DM-infusion\DM-7-scan_20220... 2/8/2022 10:57:22 AM
DM-7-scan_20220207042206 #13 RT: 0.16 AV: 1 NL: 2.10E+008
T: FTMS + p ESI Full ms [80.0000-500.0000]
311.1638
161.0595
312.1669
349.1196
162.0628
350.1230
204.5777
248.0956
443.2323
304.2998
216.0857
386.1959
175.0389
416.2062
477.2264
Relative Abundance
m/z

DM8 (296.14)

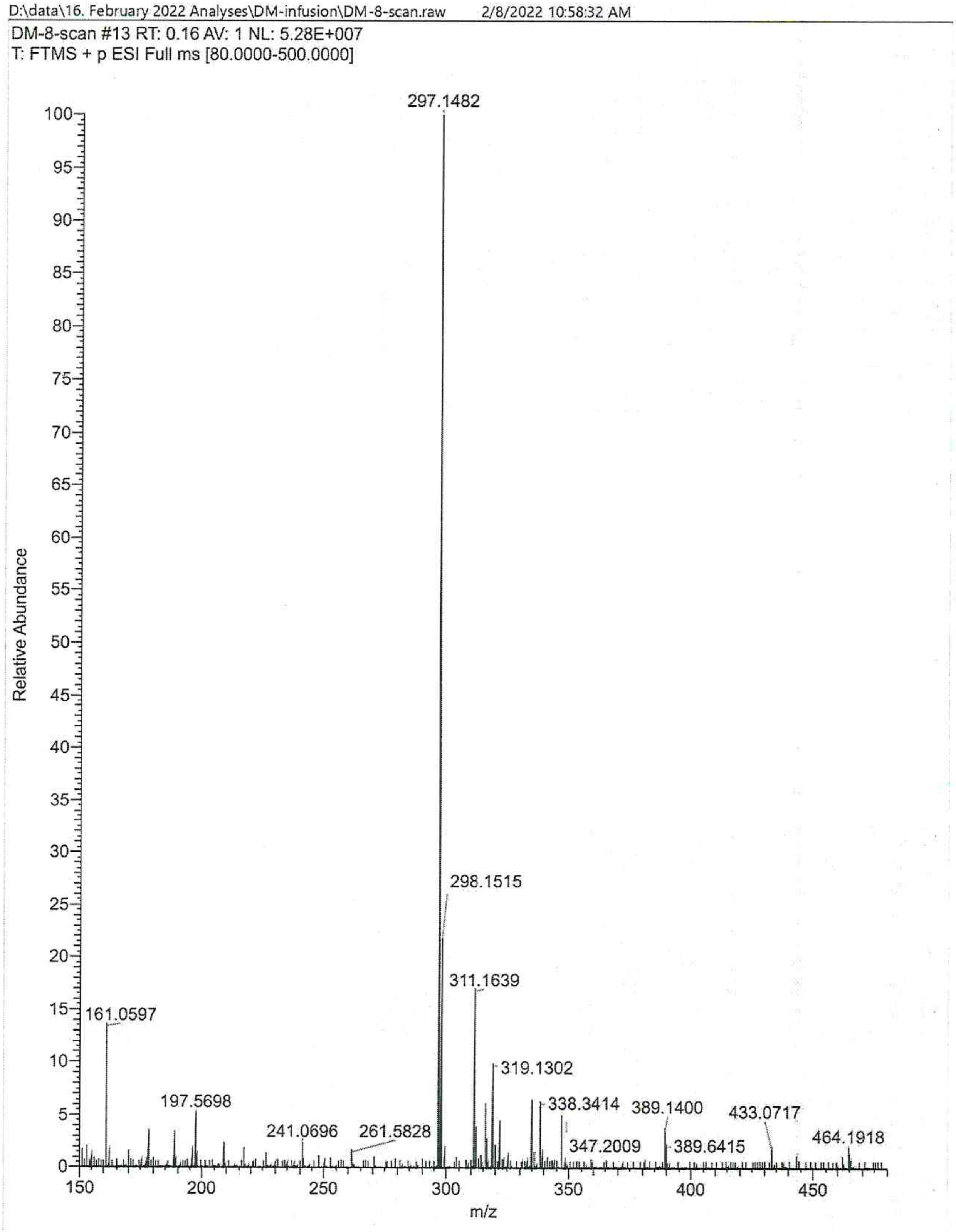

D:\data\16. February 2022 Analyses\DM-infusion\DM-8-scan.raw 2/8/2022 10:58:32 AM
DM-8-scan #13 RT: 0.16 AV: 1 NL: 5.28E+007
T: FTMS + p ESI Full ms [80.0000-500.0000]
297.1482
298.1515
311.1639
319.1302
161.0597
197.5698
241.0696
261.5828
338.3414
389.1400
433.0717
347.2009
389.6415
464.1918
Relative Abundance
m/z

DM9 (296.14)

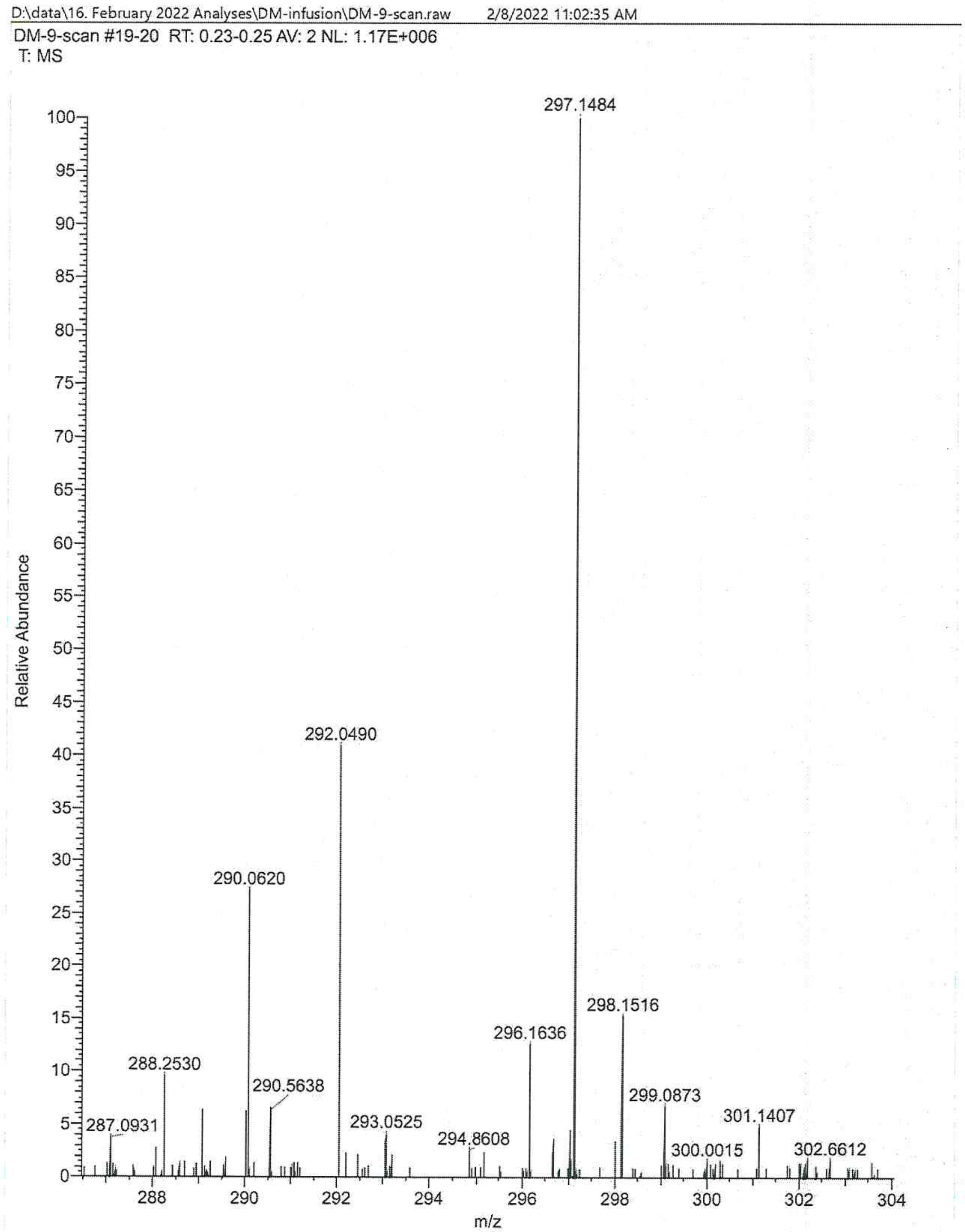
D:\data\16. February 2022 Analyses\DM-infusion\DM-9-scan.raw 2/8/2022 11:02:35 AM
DM-9-scan #19-20 RT: 0.23-0.25 AV: 2 NL: 1.17E+006
T: MS
297.1484
292.0490
290.0620
298.1516
296.1636
288.2530
290.5638
299.0873
301.1407
287.0931
293.0525
294.8608
300.0015
302.6612
Relative Abundance
m/z
100
95
90
85
80
75
70
65
60
55
50
45
40
35
30
25
20
15
10
5
0
288
290
292
294
296
298
300
302
304

DM10 (296.14)

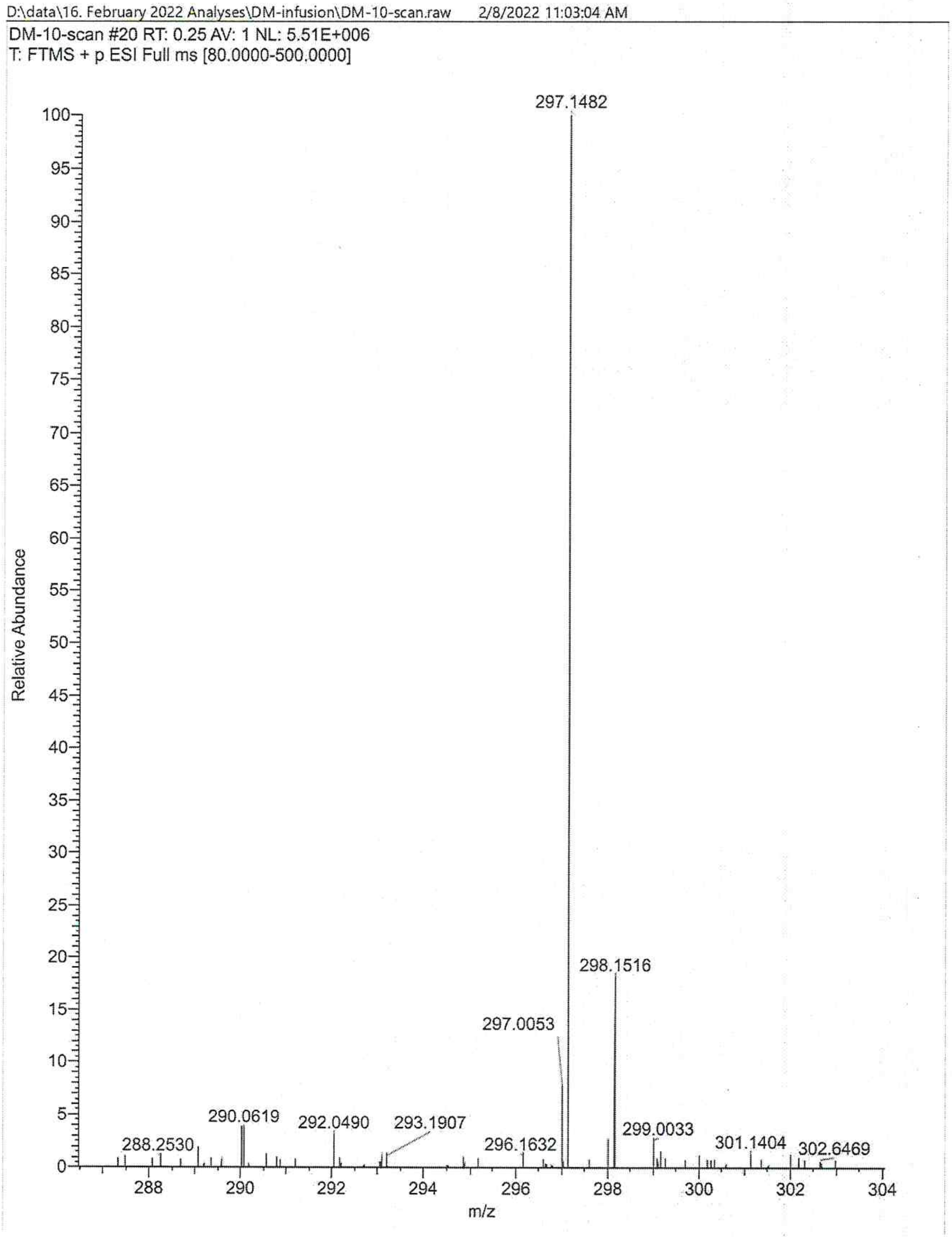
D:\data\16. February 2022 Analyses\DM-infusion\DM-10-scan.raw 2/8/2022 11:03:04 AM
DM-10-scan #20 RT: 0.25 AV: 1 NL: 5.51E+006
T: FTMS + p ESI Full ms [80.0000-500.0000]
297.1482
298.1516
297.0053
290.0619
292.0490
293.1907
288.2530
296.1632
299.0033
301.1404
302.6469
Relative Abundance
m/z

DM11 (370.18)

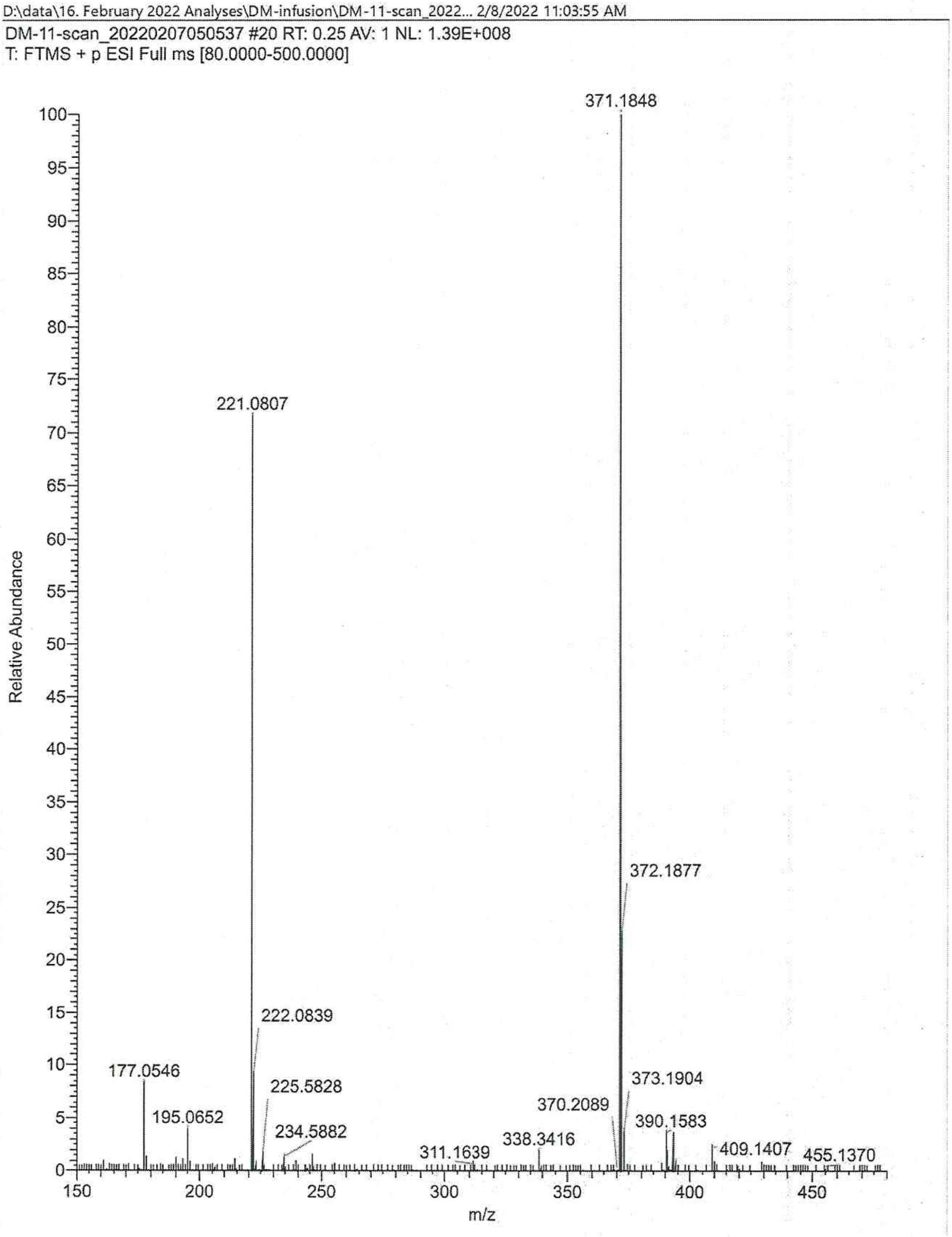

D:\data\16. February 2022 Analyses\DM-infusion\DM-11-scan_2022... 2/8/2022 11:03:55 AM
DM-11-scan_20220207050537 #20 RT: 0.25 AV: 1 NL: 1.39E+008
T: FTMS + p ESI Full ms [80.0000-500.0000]
371.1848
221.0807
372.1877
222.0839
177.0546
225.5828
373.1904
370.2089
195.0652
390.1583
234.5882
338.3416
311.1639
409.1407
455.1370
Relative Abundance
m/z
